# (+)-/(−)-Ormohenins A and B, two pairs of ormosanine-type enantiomers and their derivatives with neuroprotective activity from *Ormosia henryi* Prain

**DOI:** 10.1007/s13659-025-00539-2

**Published:** 2025-08-25

**Authors:** Ming Cheng, Xian-Si Zeng, Zhao-Yun Yin, Xiao-Yan Xie, Jia-Wen Zhu, Jian-Feng Wang, Ying-Kun Sheng, Jin-Biao Xu

**Affiliations:** 1 Xingzhi College, Zhejiang Normal University, Jinhua, 321004 China; 2 College of Medicine, Jiaxing University, Jiaxing, 314001 China

**Keywords:** *Ormosia henryi*, Ormosanine-type alkaloids, Specific rotation calculations, AChE inhibitory, Neuroprotective activity

## Abstract

Two pairs of undescribed alkaloid enantiomers, (+)-/(−)-ormohenins A (**1**) and B (**2**), were isolated from the seeds of *Ormosia henryi* Prain, along with four undescribed alkaloids (**3**, **4**,** 7** and **8**) and seven known ones (**5**, **6**, **9**–**13**). Compounds **1**–**6** belong to the ormosanine-type alkaloids, compounds **7**, **9**, and **11** are of the lupinine-type, compounds **8** and **10** are classified as anagyrine-type alkaloids, **12** and **13** are cytisine-type alkaloids. The chemical structures of **1**–**13** were elucidated through comprehensive NMR and MS data analyses. Furthermore, the racemates (±)-**1** and (±)-**2** were successfully resolved into their respective optically pure enantiomers using a chiral HPLC system. The absolute configurations of compounds **1–3** were determined using single-crystal X-ray diffraction and corroborated by DFT calculations of specific rotations. The absolute configurations of **4**, **7**, and **8** were assigned by the experimental electronic circular dichroism (ECD) with those predicted using TDDFT calculations. Compound **12** exhibited significant acetylcholinesterase (AChE) inhibitory activity with the IC_50_ value of 6.581 ± 1.203 μM. The neuroprotective effects of these compounds against A*β*_25-35_ induced cell damage in PC12 cells were investigated, and compounds** 3**, **9**, and **12** exhibited significant neuroprotective effects against A*β*_25-35_ induced PC12 cell damage, with the EC_50_ values of 7.99–15.49 μM, respectively.

**Graphical Abstract:**

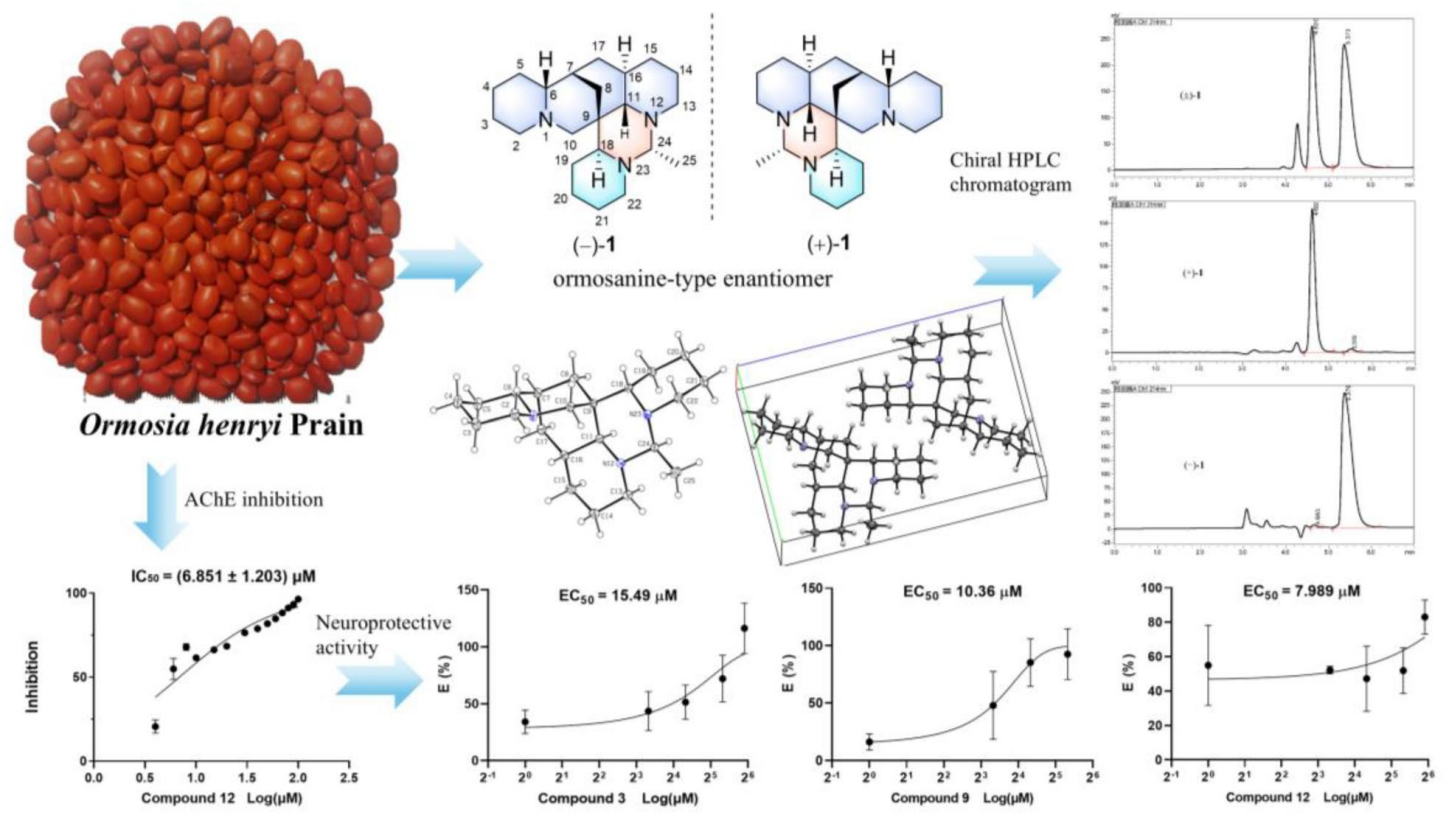

**Supplementary Information:**

The online version contains supplementary material available at 10.1007/s13659-025-00539-2.

## Introduction

Alzheimer’s disease (AD), the most common neurodegenerative disorder, is typified by the extracellular buildup of *β*-amyloid (A*β*) plaques and the intracellular formation of neurofibrillary tangles (NFTs), which are composed of hyperphosphorylated tau protein. Additionally, AD is marked by synaptic dysfunction, neuronal loss, and the activation of glial cells [1]. Current pharmacological interventions for Alzheimer’s disease (AD) predominantly focus on alleviating symptoms. The arsenal includes acetylcholinesterase (AChE) inhibitors such as donepezil, rivastigmine, and galantamine, which aim to boost acetylcholine levels and thereby enhance cognitive function. Additionally, the *N*-methyl-D-aspartate (NMDA) receptor antagonist memantine is employed to modulate glutamate activity and protect neurons from overstimulation. More recently, anti-A*β* monoclonal antibodies like lecanemab and aducanumab have been introduced to target and clear A*β* plaques [2, 3]. While these medications can enhance cognitive abilities and daily functioning, or decelerate the pace of functional and cognitive deterioration, they are not capable of arresting the disease’s progression. Given these limitations, there is an urgent need to identify and develop innovative therapeutic agents that can effectively address the underlying pathophysiology of AD and potentially alter its course.

**Fig. 1:**
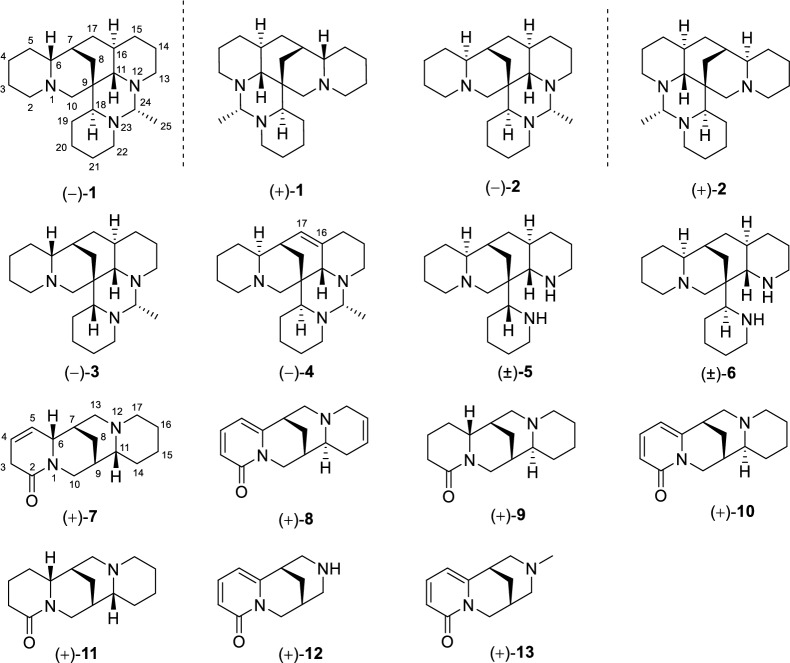
Chemical structures of **1–13**

Natural products, with their rich chemical diversity and unique structural features, have garnered significant attention for their potential to prevent neurodegeneration and enhance cognitive functions. Natural compounds often possess a wide array of pharmacological actions that can modulate multiple pathways implicated in neurodegenerative diseases. Their inherent complexity and multifunctionality enable them to interact with various biological targets, offering a holistic approach to combating neurodegeneration [4]. *Ormosia henryi* Prain, a member of the *Ormosia* genus, is a perennial evergreen tree commonly found in the southern regions of China [5]. This plant has a long history of use in traditional Chinese folk medicine, where its roots, leaves and stem bark have been applied to treat swallowing disorders, pain, and inflammation [6]. Clinical observations suggest that the leaves of *O. henryi* Prain have the effects of refreshing, invigorating, and antidepressant, hinting at its potential in the treatment of depression [7, 8]. Despite these promising traditional applications, there has been a dearth of scientific research into the plant’s chemical constituents and pharmacological properties. Preliminary studies have identified the presence of flavonoids [9] and alkaloids [10, 11] in *O. henryi* Prain. As a potential renewable resource, a more in-depth investigation into the chemical composition and pharmacological activities of *O. henryi* Prain is warranted. In our recent research, we have successfully isolated two pairs of new alkaloid enantiomers, namely (+)-/(−)-ormohenins A (**1**) and B (**2**), along with four new alkaloids, (−)-ormohenin C (**3**), (−)-ormohenin D (**4**), (6*R*,7*S*,9*S*,11*R*)-4,5-dehydro-*α*-isolupanine (**7**), and (7*S*,9*S*,11*S*)-15,16-dehydroanagyrine (**8**). We also identified seven known alkaloids, namely (±)-18-epiormosanine (**5**), (±)-ormosanine (**6**), (+)-lupanine (**9**), (+)-anagyrine (**10**), (+)-*α*-isolupanine (**11**), (+)-cytisine (**12**), and (+)-*N*-methylcytisine (**13**) (Fig. 1).

## Results and discussion

**Fig. 2:**
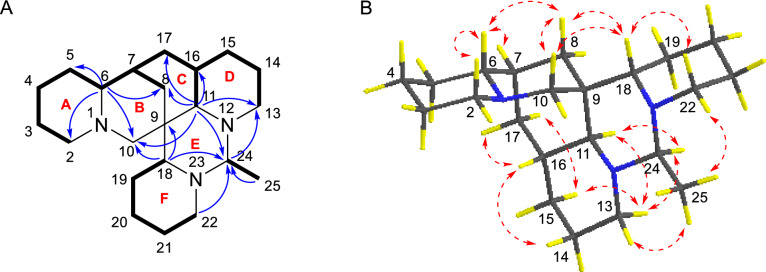
**A** ^1^H-^1^H COSYs (—) and selected HMBCs (→) of** 1**. **B** Key ROESY correlations (↔) of **1**

Compound **1** was isolated as colorless crystals. Its molecular formula was determined to be C_22_H_37_N_3_, with six double bond equivalents (DBEs), based on the HRESIMS ion [M + H]^+^ at *m/z* 344.3061 (calcd for 344.3065). The ^1^H NMR and ^13^C NMR spectrum (Tables 1 and 3) revealed 22 carbon signals for compound **1**, consisting of a doublet methyl group [*δ*_H-25_ 1.23 (d, *J* = 5.5 Hz),* δ*_C-25_ 17.0], 14 methylenes, six methines, and a nonprotonated carbon (*δ*_C-9_ 37.3). The ^1^H-^1^H COSY spectrum displayed three spin–spin systems: H-2/H-3/H-4/H-5/H-6/H-7/H-17(H-8)/H-16/H-15(H-11)/H-14/H-13, H-18/H-19/H-20/H-21/H-22, and H-24/H-25 (Fig. 2A). HMBC correlations (Fig. 2A) from H-6 to C-2, C-5, C-8 and C-10 confirmed that rings A and B were fused via N-1 and C-6. Further HMBCs from H-11 to C-8, C-10, C-13, C-16, and C-17 indicated rings B and D were connected through ring C. Ring E was positioned adjacent to rings C and D, as evidenced by HMBC correlations of H-11/C-8, C-10, C-13, C-24, and H-24/C-13. The connectivity of ring F to ring E was corroborated by the HMBC cross-peaks of H-18/C-9, C-24, and H-22/C-24. The planar structure of **1** was closely related to the known ormosia-type quinolizidine alkaloid, homoormosanine [10]. The primary difference in their 1D NMR spectra between them was the presence of an additional doublet methyl (C-25) signal in **1**, which was assignable to C-24 supported by the HMBC correlations from C*H*_3_-25 to C-24.

**Table 1:** ^1^H NMR Spectroscopic Data (600 MHz) for **1**–**4** in CD_3_OD

No.	*δ*_H_ (*J* in Hz)			
	1	2	3	4
2	*α* 2.80 br d (11.0)	*α* 2.08 td (11.4, 4.1)	*α* 2.80 br d (10.5)	*α* 2.11 td (11.5, 3.4)
	*β* 1.71 m	*β* 2.87 br d (11.4)	*β* 1.72 m	*β* 2.79 br d (11.5)
3	1.54 m	1.66 m	1.57 m	a 1.60 m b 1.66 m
4	a 1.66 m b 1.77 m	a 1.34 m b 1.75 m	1.55 m	a 1.36 m b 1.77 m
5	a 1.36 br d (12.6)	a 1.33 m	a 1.36	a 1.36 m
	b 1.52 overlapped	b 1.57 m	b 1.50 m	b 1.47 m
6	1.88 m	1.79 m	1.86 m	1.70 m
7	1.55 m	1.59 m	1.55 m	1.89 m
8	*α* 1.12 br d (12.2)	*α* 0.95 dd (13.4, 3.6)	*α* 1.11 m	*α* 1.11 dd (11.9, 3.7)
	*β* 1.47 br d (12.2)	*β* 1.59 m	*β* 1.65 m	*β* 1.56 m
10	*α* 3.81 d (11.6)	*α* 2.60 d (13.0)	*α* 3.23 d (11.3)	*α* 2.51 d (12.3)
	*β* 1.71 overlapped	*β* 2.71 d (13.0)	*β* 2.03 d (11.3)	*β* 2.82 d (12.3)
11	2.00 d (10.1)	1.78 m	1.54 m	2.69 m
13	*α* 3.26 br d (11.0)	*α* 3.21 br d (11.1)	*α* 3.27 br d (9.8)	*α* 3.16 br d (10.0)
	*β* 1.89 m	*β* 1.83 m	*β* 1.70 m	*β* 1.87 m
14	a 1.66 m	1.64 m	1.63 m	*α* 1.45 m
	b 1.77 m			*β* 1.66 m
15	*α* 0.81 m	*α* 1.02 m	*α* 0.81 m	*α* 2.22 br d (14.4)
	*β* 1.62 m	*β* 1.57 m	*β* 1.60 m	*β* 1.99 m
16	2.91 m	1.78 m	2.93 m	
17	*α* 1.08 m	*α* 1.47 m	*α* 1.08 m	5.49 m
	*β* 1.86 m	*β* 1.12 m	*β* 1.85 m	
18	2.47 br d (12.0)	2.71 m	1.63 m	2.90 dd (12.2, 2.4)
19	*α* 1.26 m *β* 1.86 m	*α* 1.25 m *β* 1.90 m	a 1.18 m b 1.76 m	*α* 1.28 m *β* 1.87 m
20	*α* 1.54 m *β* 1.93 m	*α* 1.54 m *β* 1.93 m	a 1.28 m b 1.63 m	*α* 1.54 m *β* 1.93 m
21	*α* 1.60 m *β* 1.06 m	*α* 1.59 m *β* 1.10 m	a 1.27 m b 1.68 m	*α* 1.56 m *β* 1.14 m
22	*α* 2.59 m *β* 3.38 m	*α* 2.62 m *β* 3.42 m	*α* 1.61 m *β* 3.21 m	*α* 2.65 m *β* 3.47 m
24	3.51 q (5.4)	3.45 q (5.6)	2.27 q (5.1)	3.52 q (5.8)
25	1.23 d (5.4)	1.22 d (5.6)	1.31 d (5.1)	1.23 d (5.8)

The relative configuration of **1** was elucidated through analysis of its ROESY spectrum (Fig. 2B). Key cross-peaks of H-6/H-7, H-6/H-8*β*, H-8*β*/H-10*β*, H-15*β*/H-13*β*, H-13*β*/H-11, H-11/H-24, H-15*β*/H-17*β* suggested that these hydrogens, especially H-6, H-7, H-11, and H-24, were cofacial and arbitrarily assigned as *β*-orientation. The correlations of H-18/H-10*β*, H-18/H-8*β*, were observed, which suggested that these hydrogens were spatially nearby, especially inferred the *α*-orientation of H-18. The ROESY crosspeaks of H-18/H-22*α*, H-16/H-14*α*, H-16/H-17*α*, and H-13*α*/C*H*_3_-25 suggested that these hydrogens were *α*-orientated.

Compound **2** was obtained as colorless crystals. Its HRESIMS data exhibited the [M + H]^+^ ion peak at *m/z* 344.3062 (calcd for 344.3065), consistent with a molecular formula of C_22_H_37_N_3_. The molecular formula of compound **2**, along with its hydrogen and carbon chemical shifts and the types of carbon atoms (Tables 1 and 3), are essentially the same as those of compound **1**, which allows us to infer that the gross structure of compound **2** is similar to that of compound **1**. The variations in some chemical shifts observed between them may be attributed to their differing stereoconfigurations.

**Fig. 3:**
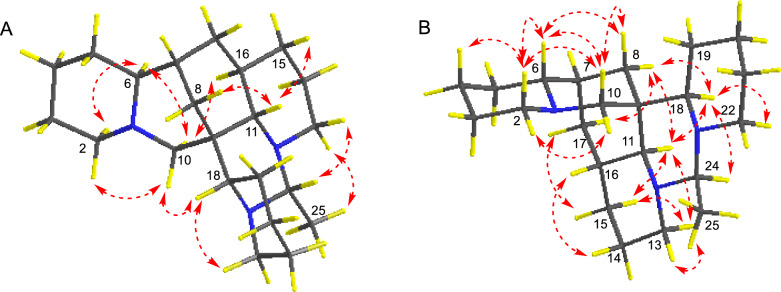
Key ROESY correlations (↔) of **2** (**A**) and **3** (**B**)

**Fig. 4:**
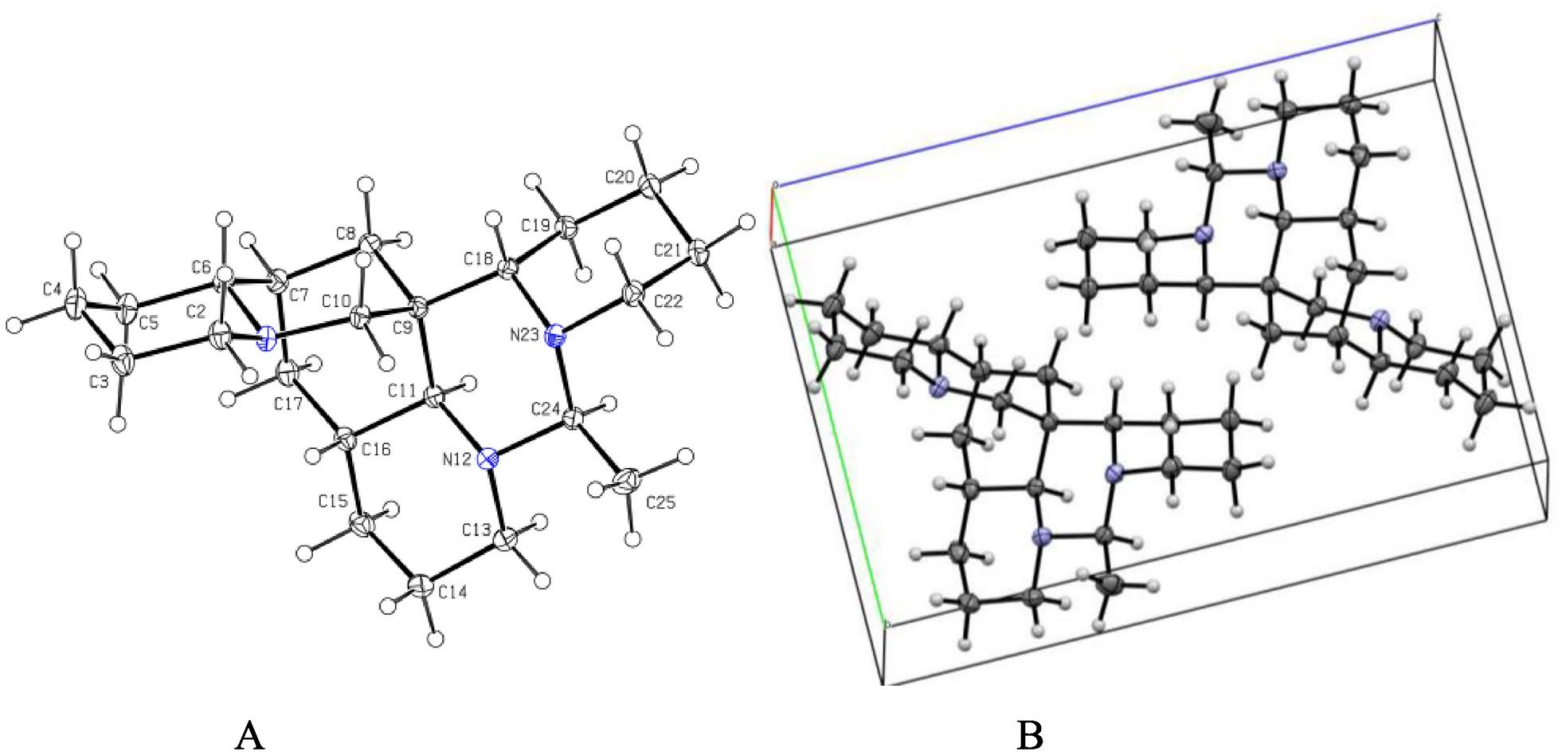
**A** X-ray crystal structure of** 1**. **B** Assembly of molecules of **1** in the crystals

**Fig. 5:**
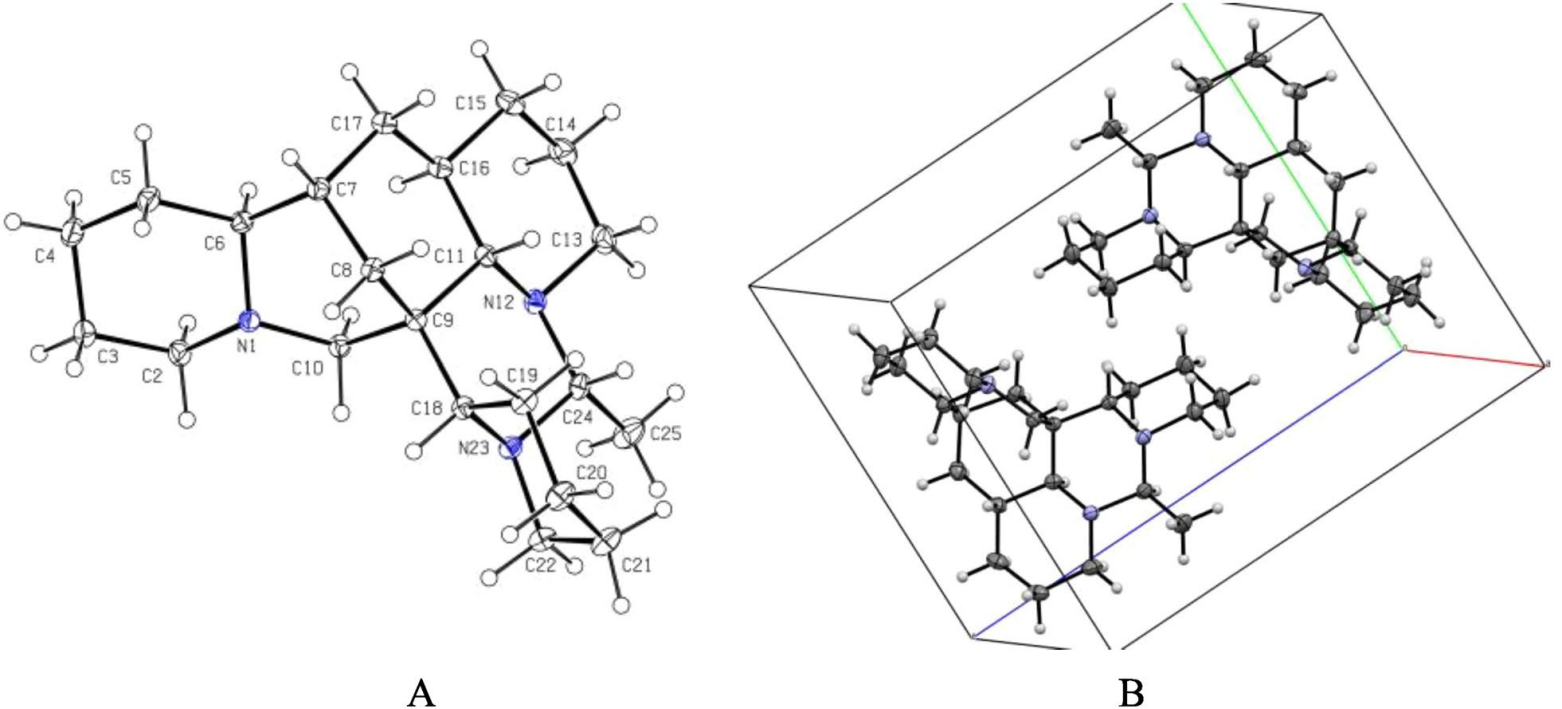
**A** X-ray crystal structure of **2**. **B** Assembly of molecules of **2** in the crystals

**Fig. 6:**
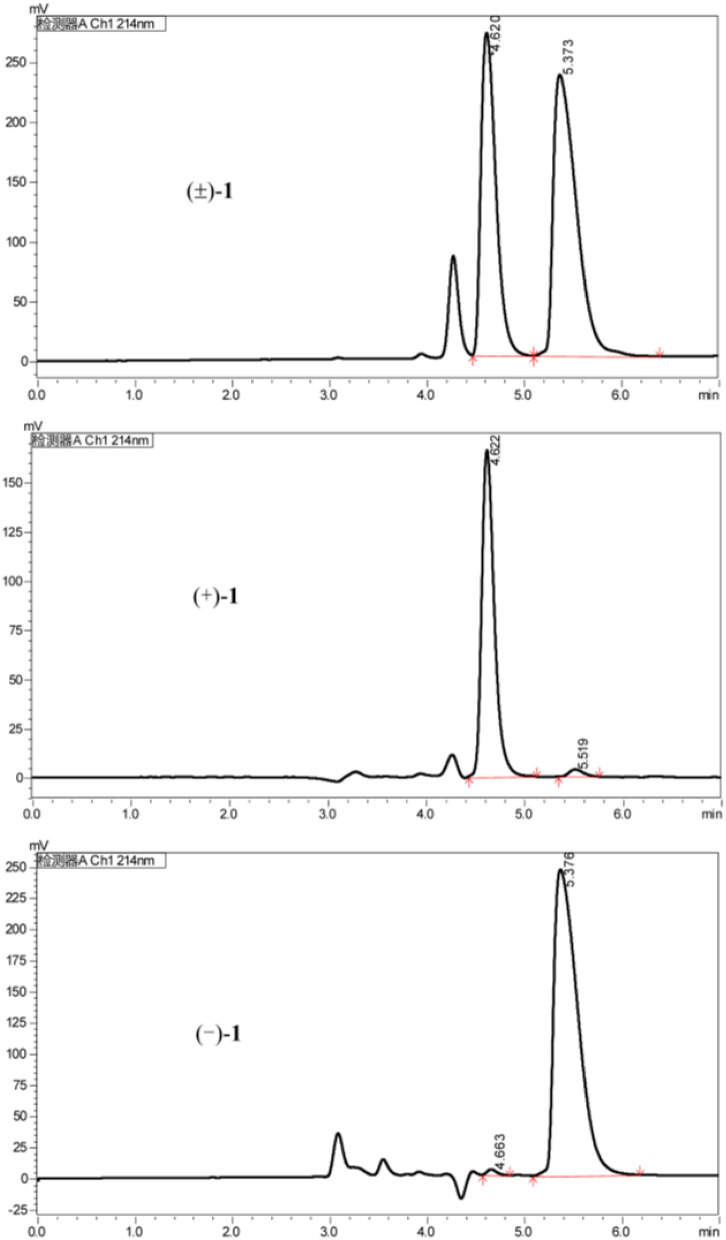
Chiral HPLC chromatogram of racemic **1** using a Chiralpak IE (IE00CE-SB022, 0.46 × 25 cm); isocratic elution with hexane/EtOH/DEA (80/20/0.1, v/v/v), flow rate 1 mL/min, UV detection at 214 nm

**Fig. 7:**
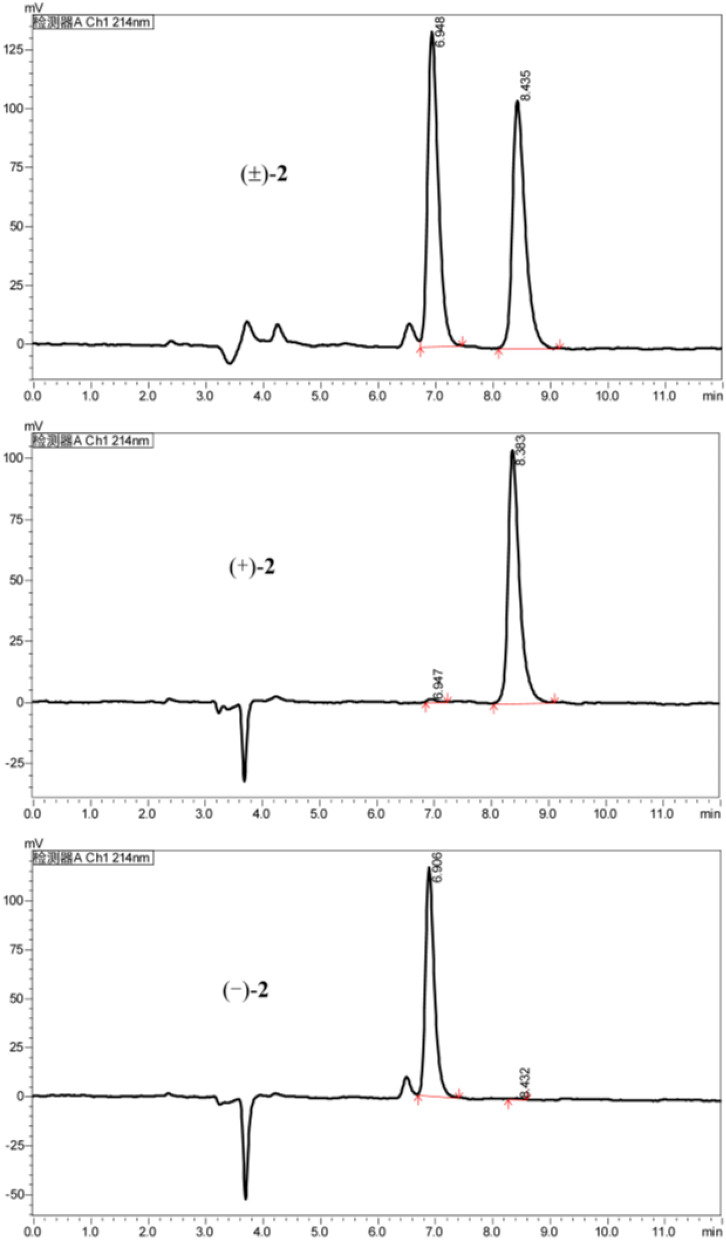
Chiral HPLC chromatogram of racemic **2** using a Chiralpak IF (IF00CE-RL017, 0.46 × 25 cm); isocratic elution with MeOH/DEA (100/0.1, v/v), flow rate 1 mL/min, UV detection at 214 nm

The analysis of the relative configuration of compound **2** was hindered due to the overlapping proton signals of H-6, H-11, H-16, and other critical hydrogens in its ROESY spectrum. This overlap can complicate the determination of the relative configuration based solely on ROESY data (Fig. 3A). However, the configuration for compounds **1** and **2** were successfully determined through single-crystal X-ray diffraction experiments. Consequently, the structures with relative configurations of **1** and **2** were unambiguously determined as depicted in Figs. 4A and 5B. Further analysis of the X-ray data revealed that both **1** and **2** possessed a centrosymmetric space group *P-1*, which is indicative of their racemic nature (Figs. 4A, 5B, and Tables S1, S2). The optical values of zero for compounds **1** and **2** also implied that they were racemic mixtures. Fortunately, chiral separations of compounds **1** and **2** were successfully performed using Chiralpak columns, resulting in the isolation of their optically pure enantiomers (Figs. 6 and 7).

Although the separation of racemates **1** and **2** was successfully achieved, pinpointing their absolute configurations remained a challenge owing to their lack of qualified crystals and chromophores in the molecules. It is well-documented that the calculation of specific rotation is a valuable technique for elucidating the absolute structure of chiral compounds [12]. Consequently, we proceeded to determine the specific rotations of the resulting chiral HPLC fractions. For **1**, chiral HPLC fraction I at a retention time (*t*_R_) of 4.62 min exhibited a specific rotation of $[\alpha]_\text{D}^{20}$ + 55.0 (*c* 0.10, MeOH), whereas fraction II at *t*_R_ = 5.37 min showed $[\alpha]_\text{D}^{20}$ − 55.0 (*c* 0.10, MeOH). Similarly, for **2**, chiral HPLC fraction I at *t*_R_ = 6.95 min had a specific rotation of $[\alpha]_\text{D}^{17}$ + 11.3 (*c* 0.10, MeOH), and fraction II at *t*_R_ = 8.43 min exhibited $[\alpha]_\text{D}^{17}$ − 11.6 (*c* 0.10, MeOH). Density functional theory (DFT) calculations were conducted on the presumed absolute configurations of structures **1** and **2** on the left side as depicted in Fig. 1. These calculations yielded specific rotation values that were negative (for details, refer to the Supporting Information). The agreement between the observed and calculated specific rotation values confirmed the absolute configurations of the separated chiral HPLC fractions for compounds **1** and **2** (Figs. 1, 6, and 7), respectively.

**Fig. 8:**
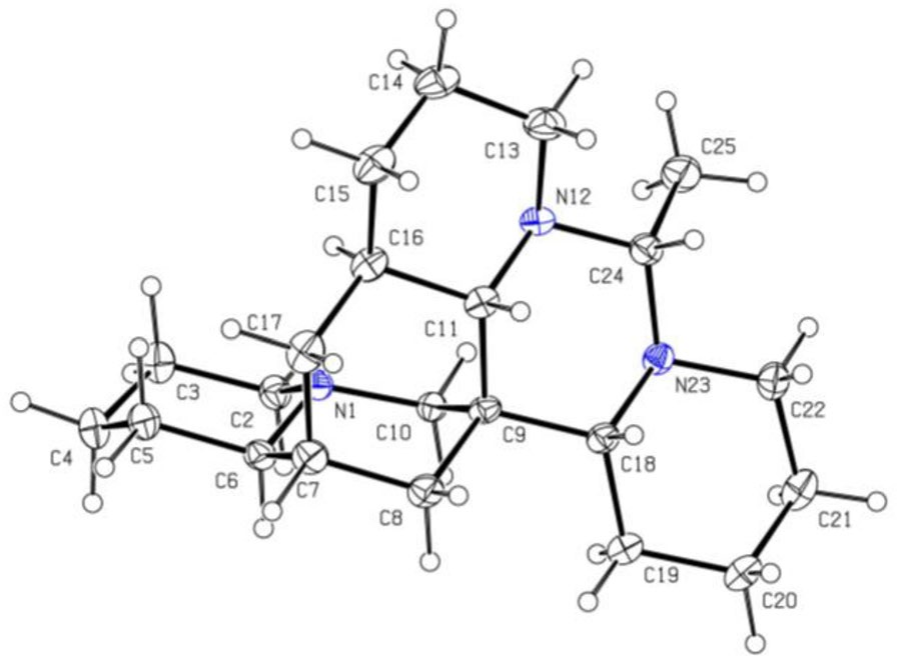
X-ray crystal structure of **3**

Compound **3** was isolated as colorless crystals, and its molecular formula was determined to be C_22_H_37_N_3_ based on the HRESIMS data, which showed an [M + H]^+^ ion peak at *m/z* 344.3060 (calcd for 344.3065). The 1D NMR spectroscopic data (Tables 1 and 3) for **3** were consistent with those of **1**, suggesting that **3** also shared the same planar structure as **1**. The relative configuration of **3** was established as shown in Fig. 3B by ROESY correlations of H-2*β*/H-4, H-6/H-2*β*, H-6/H-10*β*, H-6/H-8*β*, H-10*β*/H-2*β*, H-10*β*/H-8*β*, H-8*α*/H-11, H-2*α*/H-10*α*, H-8*α*/H-18, H-8*α*/H-17*β*, H-18/H-11, H-18/H-24, H-18/H-22*β*, H-11/H-13*β*, H-13*β*/H-15*β*, H-11/H-15*β*, H-16/H-14*α*, H-17*α*/H-15*α*, C*H*_3_-25/H-13*α*. The absolute configuration of **3** was finally determined by single crystal X-ray diffraction analysis with CuK*α* radiation (Fig. 8). The Flack parameter of 0.04 (6) [13] established unambiguously the absolute configuration of **3** as 6*R*,7*R*,9*S*,11*S*,16*R*,18*R*,24*R*, and the compound was named ormohenin C.

**Fig. 9:**
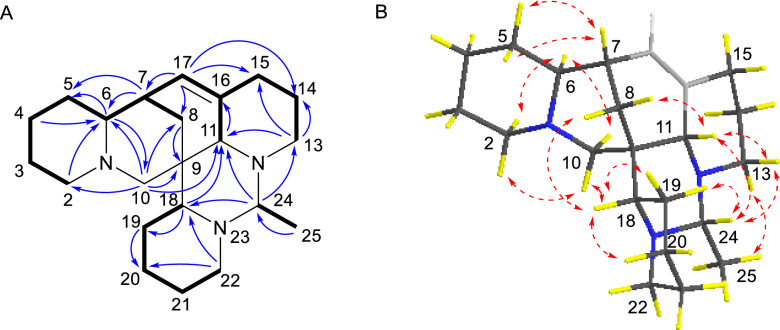
**A** ^1^H-^1^H COSYs (—) and selected HMBC correlations (→) of **4**. **B** Key ROESY correlations (↔) of** 4**

**Fig. 10:**
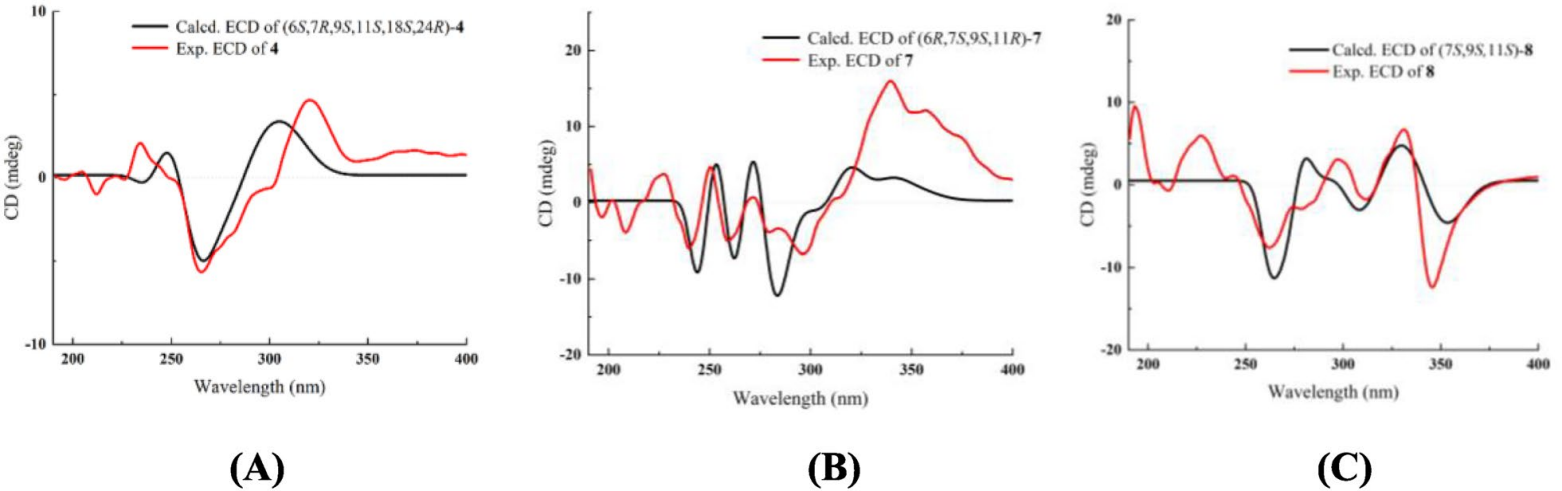
Experimental ECD and calculated ECD spectra of compounds **4**, **7**, and** 8**

Compound **4** was isolated as a yellowish oil. Its molecular formula was determined to be C_22_H_35_N_3_ with seven DBEs, based on the HRESIMS ion [M + H]^+^ at *m/z* 342.2898 (calcd for 342.2909). The NMR data (Tables 1 and 3) revealed 22 carbon signals, including four *N*-methylenes, four *N*-methines, a doublet methyl [*δ*_H-25_ 1.23 (d, *J* = 5.8 Hz),* δ*_C-25_ 16.1], and a nonprotonated carbon (*δ*_C-9_ 38.5). These features suggested that compound **4** is a structural congener of compounds **1–3**. The primary distinction lies in the presence of an olefinic proton at *δ*_H-17_ 5.48, corresponding to a double bond (*δ*_C_ 129.0 and 134.5) in **4**. The HMBC correlations (Fig. 9A) from H-17 to C-7, C-8, C-11, and C-15 assigned the Δ^16,17^ double bond. The ^1^H-^1^H COSY spectrum (Fig. 9A) also confirmed the Δ^16,17^ double bond and displayed three spin–spin systems: H-2/H-3/H-4/H-5/H-6/H-7/H-17(H-8)/H-16/H-15(H-11)/H-14/H-13, H-18/H-19/H-20/H-21/H-22, and H-24/H-25. The other HMBCs of H-7/C-5, H-6/C-5, H-6/C-10, H-4/C-6, H-2/C-6, H-10/C-2, H-10/C-6, H-10/C-9, H-10/C-8, H-10/C-11, H-13/C-11, H-13/C-14, H-18/C-11, H-18/C-19, H-22/C-18, H-24/C-11, H-24/C-18, H-24/C-13, and H-25/C-24 further determined the structure of **4** as shown. The relative configuration of **4** was established by key ROESY correlations (Fig. 9B) of H-8*β*/H-18*α*, H-8*α*/H-11*β*, H-6/H-2*α*, H-6/H-10*α*, H-7/C*H*_2_-5, H-10*β*/H-2*β*, H-10*β*/H-18, H-18/H-19*α*, H-18/H-20*α*, H-11/H-13*β*, H-19*β*/H-24, H-24/H-11*β*, H-24/H-13*β*, C*H*_3_-25/H-13*α*. The theoretical ECD spectrum of (6*S*,7*R*,9*S*,11*S*,18*S*,24*R*)-**4** was in good accordance with its experimental ECD spectrum (Fig. 10A), which allowed its absolute configuration as 6*S*,7*R*,9*S*,11*S*,18*S*,24*R*. Consequently, compound **4** was designated as ormohenin D.

Compound **7** was isolated as a yellowish oil, with a molecular formula of C_15_H_22_N_2_O, as determined by HRESIMS from a quasi-molecular ion [M + H]^+^ at *m/z* 247.1799 (calcd for 247.1820), corresponding to six DBEs. The ^1^H NMR and ^13^C NMR spectra (Tables 2 and 3) revealed the presence of 15 carbon signals, consisting of eight methylenes, six methines [including two olefinic methines (*δ*_H-4_ 5.82 br d, *J* = 10.2 Hz, *δ*_C-4_ 123.3; *δ*_H-5_ 5.54 br d, *J* = 10.2 Hz, *δ*_C-5_ 125.6)], and an amide carbonyl at *δ*_C-2_ 170.0. The presence of these functional groups, along with the remaining four DBEs, suggested that **7** was a tetracyclic alkaloid [14].

**Table 2:** ^1^H NMR Spectroscopic Data (600 MHz) for **7** and** 8** in CD_3_OD

No.	*δ*_H_ (*J* in Hz)	
	7	8
3	2.89–3.03 m	6.47 d (8.7)
4	5.82 br d (10.2)	7.31 dd (8.7, 6.2)
5	5.54 br d (10.2)	6.02 d (6.2)
6	4.07 brs	
7	2.23 m	2.96 br s
8	*α* 2.19 m	a 1.74 m
	*β* 1.49 d (12.6)	b 2.10 br d (14.6)
9	1.67 m	2.24 m
10	*α* 4.58 br d (13.0)	*α* 4.07 br d (15.2)
	*β* 2.64 br d (13.0)	*β* 4.00 m
11	1.69 m	3.21 m
13	*α* 1.86 br d (11.2)	*α* 2.52 br d (11.0)
	*β* 2.84 dd (12.3, 2.7)	*β* 3.19 br d (11.0)
14	*α* 1.58 m	*α* 1.74 m
	*β* 1.42 m	*β* 2.47 m
15	a 1.30 m	5.79 m
	b 1.74 d (12.8)	
16	1.56 m	5.63 m
17	*α* 1.94 m	a 2.73 d (18.1)
	*β* 2.78 br d (12.8)	b 3.50 d (18.1)

**Table 3:** ^13^C NMR Spectroscopic Data (150 MHz) for **1–4**, **7** and **8** in CD_3_OD

No.	*δ* in ppm					
	1	2	3	4	7	8
2	58.0 (CH_2_)	56.8 (CH_2_)	58.2 (CH_2_)	57.2 (CH_2_)	170.0 (C)	163.7 (C)
3	27.2 (CH_2_)	26.1 (CH_2_)	26.2 (CH_2_)	26.5 (CH_2_)	32.4 (CH_2_)	116.6 (CH)
4	26.2 (CH_2_)	25.9 (CH_2_)	27.1 (CH_2_)	26.2 (CH_2_)	123.3 (CH)	138.8 (CH)
5	31.5 (CH_2_)	34.9 (CH_2_)	31.5 (CH_2_)	33.6 (CH_2_)	125.6 (CH)	104.8 (CH)
6	67.5 (CH)	66.5 (CH)	67.7 (CH)	67.2 (CH)	63.0 (CH)	152.1 (C)
7	36.1 (CH)	36.3 (CH)	35.8 (CH)	37.3 (CH)	33.8 (CH)	35.9 (CH)
8	40.3 (CH_2_)	31.4 (CH_2_)	39.4 (CH_2_)	27.7 (CH_2_)	27.1 (CH_2_)	20.9 (CH_2_)
9	37.3 (C)	37.3 (C)	36.8 (C)	38.5 (C)	36.5 (CH)	31.3 (CH)
10	66.1 (CH_2_)	63.5 (CH_2_)	58.1 (CH_2_)	61.8 (CH_2_)	47.5 (CH_2_)	51.5 (CH_2_)
11	70.5 (CH)	69.9 (CH)	76.9 (CH)	65.8 (CH)	64.5 (CH)	58.3 (CH)
13	53.7 (CH_2_)	52.7 (CH_2_)	55.0 (CH_2_)	50.5 (CH_2_)	52.9 (CH_2_)	54.2 (CH_2_)
14	26.3 (CH_2_)	26.2 (CH_2_)	26.3 (CH_2_)	26.0 (CH_2_)	34.1 (CH_2_)	21.5 (CH_2_)
15	34.3 (CH_2_)	32.9 (CH_2_)	34.2 (CH_2_)	32.8 (CH_2_)	25.7 (CH_2_)	124.0 (CH)
16	36.3 (CH)	33.4 (CH)	36.3 (CH)	134.5 (C)	25.7 (CH_2_)	125.2 (CH)
17	35.4 (CH_2_)	39.5 (CH_2_)	34.7 (CH_2_)	129.0 (CH)	56.7 (CH_2_)	53.1 (CH_2_)
18	67.5 (CH)	69.2 (CH)	71.5 (CH)	66.6 (CH)		
19	18.4 (CH_2_)	18.5 (CH_2_)	25.6 (CH_2_)	19.6 (CH_2_)		
20	26.9 (CH_2_)	26.7 (CH_2_)	26.4 (CH_2_)	26.9 (CH_2_)		
21	19.5 (CH_2_)	19.8 (CH_2_)	26.1 (CH_2_)	20.5 (CH_2_)		
22	52.4 (CH_2_)	52.6 (CH_2_)	54.4 (CH_2_)	52.3 (CH_2_)		
24	72.7 (CH)	71.7 (CH)	84.2 (CH)	73.7 (CH)		
25	17.0 (CH_3_)	16.7 (CH_3_)	19.7 (CH_3_)	16.1 (CH_3_)		

**Fig. 11:**
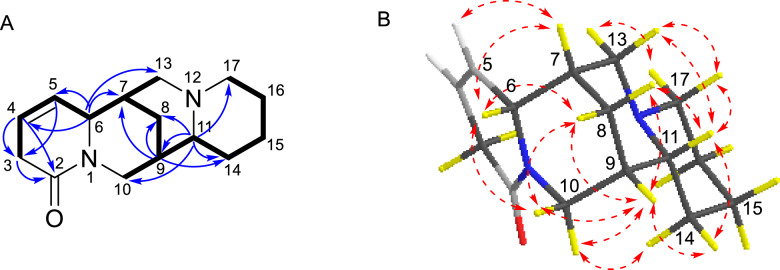
**A** ^1^H-^1^H COSY (—) and selected HMBC correlations (→) of **7**. **B** Key ROESY correlations (↔) of **7**

Compound **7** shared the same tetracyclic framework as *α*-isolupanine (**11**) [11], with the primary difference being the presence of olefinic signals in **7**. The ^1^H-^1^H COSY spectrum confirmed the Δ^4,5^ double bond and displayed a spin–spin system of H-3/H-4/H-5/H-6/H-7/H-8(H-13)/H-9/H-11(H-10)/H-14/H-15/H-16/H-17 (Fig. 11A). HMBC correlations from olefinic proton at *δ*_H-4_ 5.82 to C-2 (*δ*_C_ 170.0) and C-3 (*δ*_C_ 32.4), and from the olefinic proton at *δ*_H-5_ 5.54 to C-3 (*δ*_C_ 32.4) and C-6 (*δ*_C_ 63.0), also indicated that this double bond was located between C-4 and C-5 (Fig. 11A). The relative configuration of **7** was established through analysis of its ROESY spectrum (Fig. 11B). Key ROESY correlations of H-5/H-7, H-6/H-7, H-6/H-8*β*, H-6/H-10*β*, C*H*_2_-10/H-9 suggested that H-6, H-7, H-8*β*, H-9, and H-10*β* were cofacial and arbitrarily assigned *β*-orientation. Additionally, correlations of H-10*α*/H-14*α*, H-11/H-8*α*, H-11/H-14*β*, H-11/H-13*β*, H-11/H-17*β* were observed, suggesting that H-11 were *β*-oriented. The absolute configuration of **7** was determined by the experimental ECD and calculated ECD spectra. The calculated ECD spectrum of (6*R*,7*S*,9*S*,11*R*)-**7** matched very well with the experimental ECD spectrum (Fig. 10B). Therefore, compound **7** was identified as (6*R*,7*S*,9*S*,11*R*)-4,5-dehydro-*α*-isolupanine.

Compound **8** emerged as a new natural constituent, isolated as yellowish oil. Its molecular formula was deduced to be C_15_H_18_N_2_O, supported by HR-ESI-MS data exhibiting a quasi-molecular ion [M + H]^+^ at *m/z* 243.1491 (calcd 243.1492). The 1D and 2D NMR spectroscopic data of **8** (Tables 2 and 3), including ROESY data, were found to be identical to those of a synthetic intermediate **S5** [15], suggesting that both compounds share the same planar structure and relative configurations. The specific rotation of **8** was determined to be $[\alpha]_\text{D}^{20}$ + 57.5, which was in opposition to the synthetic intermediate S5 with a specific rotation of $[\alpha]_\text{D}^{25}$ − 100.8. This disparity in optical activity values indicated that **8** was the enantiomer of **S5**. In addition, comparison of the experimental ECD and calculated ECD spectra of **8** also supported the 7*S*,9*S*,11*S*-configuration (Fig. 10C). Consequently, compound **8** was named (7*S*, 9*S*, 11*S*)-15,16-dehydro-anagyrine.

Seven known alkaloids were also isolated. Compounds **5** and **6** were obtained as crystals and identified as enantiomers, as evidenced by their specific rotations of zero. By comparing their spectroscopic data and optical rotations with the reported data in the literatures, the structures of known ones were unambiguously identified as (±)-18-epiormosanine (**5**) [16–18], (±)-ormosanine (**6**) [10, 18], (+)-lupanine (**9**) [10], (+)-anagyrine (**10**) [19–21], (+)-*α*-isolupanine (**11**) [11], (+)-cytisine (**12**) [22], and (+)-*N*-methylcytisine (**13**) [22].

The first and the main class of drugs currently used for AD are the AChE inhibitors and are widely used for the symptomatic treatment of mild-moderate AD [2]. All alkaloids were screened toward AChE via improved Ellman’s method (Supporting Information). Compound **12** displayed significant AChE inhibitory activity, with an IC_50_ value of 6.851 ± 1.203 μM. Huperzine A was used as a positive control, with an IC_50_ value of 1.173 ± 0.223 μM.

Meanwhile, all isolated compounds were evaluated for their neuroprotective potency against the A*β*_25-35_ induced PC12 cell death by MTT assay. The result (Table 4 and Supporting Information) showed that compounds **3**, **9**, and **12** exhibited neuroprotective effects against A*β*_25-35_ induced PC12 cell damage. Among them, Compound **12** displayed the most neuroprotective activity, with an EC_50_ value of 7.99 μM. Resveratrol was used as a positive control [23], with an EC_50_ value of 5.99 μM.

**Table 4:** The neuroprotective effects of compounds **1**–**12** against A*β*_25-35_-induced cell damage in PC12 cells

Compound	Cell viability	EC_50_ (μM)	Compound	Cell viability	EC_50_ (μM)
1	0.641 ± 0.009	–	**9**	0.974 ± 0.065***	10.36
2	0.693 ± 0.024	–	**10**	0.645 ± 0.011	–
3	0.899 ± 0.061***	15.49	**11**	0.653 ± 0.027	–
4	0.629 ± 0.013	–	**12**	0.934 ± 0.031***	7.99
5	0.698 ± 0.032	–	Control	1.000 ± 0.008***	–
6	0.704 ± 0.038	–	Model (A*β*_25-35_)	0.633 ± 0.021	–
7	0.650 ± 0.009		Resveratrol	0.82 ± 0.039****	5.99
8	0.662 ± 0.041	–			

^***^p < 0.001, ****p < 0.0001 compared with the model group

## Experimental section

### General experimental procedures

Optical rotations were measured using a Rudolph Research Analytical Autopol VI automatic polarimeter (Rudolph Research Analytical, NJ, USA). Melting points were determined with a YRT-3 melting point apparatus (Tianjin, China). UV spectra were recorded on an Evolution 300 PC UV–visible spectrophotometer (Thermo, USA). IR spectra were obtained using a Thermo Fisher IS50 spectrometer (Nicolet, USA) and a Bruker VERTEX 70 spectrometer (Germany), with samples prepared as KBr disks. ECD spectra were recorded on a Bio-Logic Science MOS-500 spectrometer. 1D and 2D NMR spectra were acquired on Bruker AV4600 NMR spectrometers, with TMS serving as the internal standard. HR-ESI–MS analyses were performed on Agilent 6545 and G6230A mass spectrometers. Single-crystal-X-ray diffractions were conducted using a Bruker APEX-II CCD detector with CuK*α* and MoK*α* radiations. Semi-preparative HPLC was carried out on a Waters 1525 pump equipped with a Waters 2489 detector and a YMC-Pack ODS-A column (250 × 10 mm, S-5 μm, 12 nm, Japan). Chiral HPLC separations were performed on a Shimadzu LC-20AD system using CHIRALPAK IE (IE00CE-SB022, 0.46 × 25 cm) and CHIRALPAK IF (IF00CE-RL017, 0.46 × 25 cm) columns. Normal phase column chromatography was conducted using silica gel (100–200 mesh). Reversed-phase column chromatography employed C_18_ silica gel (150–200 mesh, Merck) and Sephadex LH-20 (Amersham Biosciences). Precoated silica gel GF_254_ plates (Qingdao Marine Chemical Plant, Qingdao, China) were utilized for TLC analysis.

### Plant material

The seeds of *O. henryi* Prain were collected from Lishui of Zhejiang Province, China. A voucher sample (HLM-2020511s) was deposited in College of Xingzhi, Zhejiang Normal University.

### Extraction and isolation

The air-dried powders of the seeds of *O. henryi* Prain (2.0 kg) were extracted three times with 95% EtOH at ambient temperature. The crude extract (144.1 g) was dissolved in water, adjusted to pH 2 with 2% H_2_SO_4_, and then adjusted to pH 11 with 2 mol/L NaOH. Subsequent extraction with a CHCl_3_/H_2_O soluble system provided a crude CHCl_3_ extract (87.6 g). The crude extract was subjected to silica gel column chromatography (CC) and eluted with a gradient of CHCl_3_/MeOH (50: 1–1: 1) to yield three fractions (Fr. A–C).

Fr. A (25.8 g) was further separated using C18 reversed-phase silica and eluted with a gradient of H_2_O/MeOH (20–90%), resulting in subfractions A1–A4. Fr. A1 (1.8 g) was separated using silica gel CC with a CHCl_3_/MeOH gradient (100: 1–1: 1) to yield A1a and A1b. Fr. A1a (0.8 g) was further separated by passage over an Sephadex LH-20 CC (EtOH) and silica gel CC (CHCl_3_/MeOH, 100: 1–10: 1) to give subfractions A1a2a and A1a2b. Compounds **7** (*t*_R_ = 28 min, 39.8 mg), **8** (*t*_R_ = 34 min, 6.5 mg), **9** (*t*_R_ = 30 min, 78.8 mg) and **10** (*t*_R_ = 52 min, 5.4 mg) were purified from fr. A1a2a by semipreparative HPLC with an eluent of 20% ACN/H_2_O. Fr. A2 (7.8 g) was processed through silica gel CC, C_18_ reversed-phase silica, and LH-20 CC to yield four subfractions, A2b1a–A2b1d. Compound **5** (8.1 mg) was obtained from the fr. A2b1a (0.4 g) by silica gel CC and crystallization in MeOH. Fr. A2b1c (0.5 g) was separated into two subfractions, A2b1c1A and A2b1c1B, using silica gel CC and C_18_ reversed-phase silica.** 2** (78.1 mg) was obtained by crystallization of fr. A2b1c1B in MeOH. Fr. A4 (5.1 g) was separated using silica gel CC with a CHCl_3_/MeOH gradient (100: 1–10: 1) to yield four subfractions, A4a–A4d. Fr. A4a (0.9 g) was further separated over an Sephadex LH-20 CC and C_18_ reversed-phase CC to give three subfractions, A4a1-A4a3. Semi-preparative HPLC of fr. A4a3 (0.16 g) with 90% ACN/H_2_O yielded compounds **1** (*t*_R_ = 55 min, 52.0 mg) and **3** (*t*_R_ = 59 min, 42.9 mg). Semi-preparative HPLC of fr. A4a2 (0.28 g) with 87.5% MeOH/H_2_O yielded **4** (*t*_R_ = 45.5 min, 46.0 mg). Compound **6** (26.1 mg) was obtained by crystallization of fr. A4b in MeOH. Compound **11** (38 mg) was obtained from fr. A4c by silica gel CC and Sephadex LH-20 CC, respectively.

Compound **12** (154 mg) was obtained from fr. C (18.5 g) by silica gel CC and Semi-preparative HPLC (50% MeOH/H_2_O, *t*_R_ = 9.0 min). Fr. B (2.25 g) was further separated using C18 reversed-phase silica (H_2_O/MeOH 20–90%) and then silica gel CC (CH_2_Cl_2_/MeOH) to yield **13** (123 mg).

Subsequently separation of **1** by semi-preparative HPLC using a normal phase chiral column (Chiralpak IE) and eluting with n-hexane/EtOH/DEA (80: 20: 0.1) obtained (+)-**1** (flow rate 1.0 mL/min, *t*_R_ = 4.62 min) and (−)-**1** (*t*_R_ = 5.37 min), respectively. Separation of **2** by semi-preparative HPLC using a reverse phase chiral column (Chiralpak IF) and eluting with EtOH/DEA (100: 0.1) obtained (+)-**2** (flow rate 1.0 mL/min, *t*_R_ = 6.91 min) and (−)-**2** (*t*_R_ = 8.38 min), respectively.

### Spectroscopic data of compounds

#### (±)-Ormohenin A (1)

Colorless needles (MeOH); m.p. 143.9 °C; $[\alpha]_\text{D}^{20}$ ± 0 (*c* 0.05, MeOH); UV (MeOH) *λ*_max_ (log *ε*) 213 (2.16) nm; IR(KBr) *ν*_max_ 3437, 2933, 2830, 1597, 1383, 1354, 775 cm^−1^; ^1^H and ^13^C NMR date (see Tables 1 and 3); HR-ESI-MS *m/z* 344.3061 [M + H]^+^ (cacld for 344.3065).

(−)-**1**: colorless oil; $[\alpha]_\text{D}^{20}$ -55.0 (*c* 0.10, MeOH); ^1^H and ^13^C NMR date (see Tables 1 and 3); HR-ESI-MS *m/z* 344.3061 [M + H]^+^ (cacld for 344.3065).

(+)-**1**: colorless oil; $[\alpha]_\text{D}^{20}$ + 55.0 (*c* 0.10, MeOH); NMR data are the same as those of (−)-**1**.

#### (±)-Ormohenin B (2)

Colorless needles (MeOH); m.p. 145.1 °C $[\alpha]_\text{D}^{17}$ ± 0 (*c* 0.28, MeOH); UV (MeOH) *λ*_max_ (log *ε*) 215 (1.98) nm; IR(KBr) *ν*_max_ 3436, 2832, 1596, 1383, 1355, 775 cm^−1^; ^1^H and ^13^C NMR date (see Tables 1 and 3); HR-ESI-MS *m/z* 344.3062 [M + H]^+^ (cacld for 344.3065).

(−)-**2**: colorless oil; $[\alpha]_\text{D}^{17}$ − 11.6 (*c* 0.10, MeOH); ^1^H and ^13^C NMR date (see Tables 1 and 3); HR-ESI-MS *m/z* 344.3062 [M + H]^+^ (cacld for 344.3065).

(+)-**2**: colorless oil; $[\alpha]_\text{D}^{17}$ + 11.3 (*c* 0.10, MeOH); NMR data are the same as those of (-)-**2**.

#### Ormohenin C (3)

Colorless needles (MeOH); m.p. 140.0 °C; $[\alpha]_\text{D}^{20}$ − 28.8 (*c* 0.10, MeOH); UV (MeOH) *λ*_max_ (log *ε*) 209 (2.57) nm; IR(KBr) *ν*_max_ 3437, 2933, 2830, 1597, 1383, 1354, 775 cm^−1^; ^1^H and ^13^C NMR data (see Tables 1 and 3); HR-ESI-MS *m/z* 344.3060 [M + H]^+^ (cacld for 344.3065).

#### Ormohenin (4)

Yellowish oil; $[\alpha]_\text{D}^{20}$ − 130.0 (*c* 0.05, MeOH); UV (MeOH) *λ*_max_ (log *ε*) 206 (2.46) nm; IR(KBr) *ν*_max_ 2926, 2851, 1653, 1444, 1371, 1339, 1174, 1129, 1051 cm^−1^; ^1^H and ^13^C NMR data (see Tables 1 and 3); HR-ESI-MS *m/z* 342.2898 [M + H]^+^ (cacld for 342.2909).

#### (6*R*,7*S*,9*S*,11*R*)-4,5-Dehydro-*α*-isolupanine (7)

Yellowish oil; $[\alpha]_\text{D}^{20}$ + 99.0 (*c* 0.10, MeOH); UV (MeOH) *λ*_max_ (log *ε*) 303 (1.83), 211(2.53) nm; IR(KBr) *ν*_max_ 3432, 2828, 2730, 1597, 1383, 1353, 773, 558 cm^−1^; ^1^H and ^13^C NMR date (see Tables 2 and 3); HR-ESI-MS *m/z* 247.1799 [M + H]^+^ (cacld for 247.1820).

#### (7*S*,9*S*,11*S*)-15,16-Dehydroanagyrine (8)

Yellowish oil; $[\alpha]_\text{D}^{20}$ + 57.5 (*c* 0.04, MeOH); UV (MeOH) *λ*_max_ (log *ε*) 207 (2.8), 309 (3.82) nm; IR(KBr) *ν*_max_ 3362, 2923, 2853, 1646, 1600, 1550, 1459, 1251, 1137, 1047, 877, 532 cm^−1^; ^1^H and ^13^C NMR date (see Tables 2 and 3); HR-ESI-MS *m/z* 243.1491 [M + H]^+^ (cacld for 243.1492).

#### The optical rotations of 5, 6, 9–13

*18-Epiormosanine* (**5**): colorless needles (MeOH); m.p. 220.0 ~ 221.0 °C; $[\alpha]_\text{D}^{20}$ 0 (*c* 0.10, CH_2_Cl_2_); *Ormosanine* (**6**): colorless needles (MeOH); m.p. 179.0 ~ 180.0 °C; $[\alpha]_\text{D}^{20}$ 0 (*c* 0.10, CH_2_Cl_2_); The optical rotation of **9**: $[\alpha]_\text{D}^{20}$ + 71.3 (*c* 0.10, MeOH); The optical rotation of **10**: $[\alpha]_\text{D}^{20}$ + 47.7 (*c* 0.04, MeOH); The optical rotation of **11**: $[\alpha]_\text{D}^{20}$ + 56.0 (*c* 0.40, MeOH); The optical rotation of **12**: $[\alpha]_\text{D}^{20}$ + 109.2 (*c* 0.13, MeOH); The optical rotation of **13**: $[\alpha]_\text{D}^{23}$ + 68.0 (*c* 0.10, MeOH).

### Crystallographic data

Compounds **1**–**3** were recrystallized from MeOH to give colorless needles at room temperature. Crystal data was obtained on a Bruker APEX-II CCD diffractometer employing graphite monochromated Cu-K*α* radiation (*λ* = 1.54178 Å) or Mo-K*α* radiation (*λ* = 0.71073 Å) at 170.0 K. Using Olex2 [25], the structure was solved with the ShelXT [26] structure solution program using Intrinsic Phasing and refined with the ShelXL refinement package using Least Squares minimization. Crystallographic data (excluding structure factor tables) for **1**–**3** have been deposited at the Cambridge Crystallographic Data Center (CCDC number 2457561 for **1**, deposition number: 2457563 for **2**, 2,457,567 for **3**. Copies of the data can be obtained free of charge via the internet at www.ccdc.cam.ac.uk/conts or upon application to CCDC, 12, Union Road, Cambridge CB21EZ, UK [tel: (+ 44) 1223-336-408; Fax: (+ 44) 1223-336-033; e-mail: deposit@ccdc.cam.ac.uk].

Ormohenin A (**1**): m.p. 143.9 °C. Crystal data for C_22_H_37_N_3_ (*M* = 343.54 g/mol): triclinic, space group P-1 (no. 2), *a* = 6.8445(4) Å, *b* = 9.8999(5) Å, *c* = 14.9406(8) Å, *α* = 88.184(2)°, *β* = 83.162(2)°, *γ* = 69.941(2)°, *V* = 944.15(9) Å^3^, *Z* = 2, *T* = 170.0 K, μ(CuKα) = 0.534 mm^−1^, *Dcalc* = 1.208 g/cm^3^, 26,269 reflections measured (5.958° ≤ 2Θ ≤ 137.378°), 3461 unique (*R*_int_ = 0.0299, *R*_sigma_ = 0.0223) which were used in all calculations. The final *R*_1_ was 0.0389 (I > 2σ(I)) and *wR*_2_ was 0.1026 (all data).

Ormohenin B (**2**): m.p. 145.1 °C. Crystal data for C_22_H_37_N_3_ (*M* = 343.54 g/mol): triclinic, space group P-1 (no. 2), *a* = 6.8085(2) Å, *b* = 10.6845(4) Å, *c* = 13.8387(5) Å, *α* = 90.243(2)°, *β* = 98.8520(10)°, *γ* = 106.3930(10)°, *V* = 953.07(6) Å^3^, *Z* = 2, *T* = 170.0 K, μ(MoKα) = 0.070 mm^−1^, *Dcalc* = 1.197 g/cm^3^, 24,182 reflections measured (4.852° ≤ 2Θ ≤ 67.15°), 6459 unique (*R*_int_ = 0.0308, *R*_sigma_ = 0.0337) which were used in all calculations. The final *R*_1_ was 0.0533 (I > 2σ(I)) and *wR*_2_ was 0.1453 (all data).

Ormohenin C (**3**): m.p. 143.9 °C. Crystal data for C_22_H_37_N_3_ (*M* = 343.54 g/mol): orthorhombic, space group P2_1_2_1_2_1_ (no. 19), *a* = 9.3517(2) Å, *b* = 14.1492(3) Å, *c* = 14.8653(3) Å, *V* = 1966.96(7) Å^3^, *Z* = 4, *T* = 170.0 K, μ(CuKα) = 0.513 mm^−1^, *Dcalc* = 1.160 g/cm^3^, 20,384 reflections measured (8.628° ≤ 2Θ ≤ 136.442°), 3585 unique (*R*_int_ = 0.0243, *R*_sigma_ = 0.0174) which were used in all calculations. The final *R*_1_ was 0.0317 (I > 2σ(I)) and *wR*_2_ was 0.0838 (all data). Flack parameter = 0.04(6).

### Specific rotation and ECD calculations

Density functional theory (DFT) calculations of specific rotation and ECD are described in the Supporting Information.

### AChE inhibitory activity assay

The AChE inhibitory activity of isolated compounds was assessed with a modified spectrophotometric method [27, 28], using huperzine A as the positive controls (see details in the Supporting Information).

### Neuroprotective activity assay

A cell viability assay was conducted to test the cytotoxic effects of the compounds on PC12 cells. Meanwhile, the neuroprotective activity of all compounds against the A*β*_25-35_ induced PC12 cell death by MTT assay. PC12 cells were divided into four groups: normal control, model, positive control (Resveratrol), and isolates intervention groups (1, 10, 20, 40, 60, 80, 100 μmol/L). Detailed experimental procedures and experimental data can be found in the Supporting Information.

## Conclusion

*Ormosia* plants are rich in alkaloids, which can be classified into ormosanine-type, lupinine-type, anagyrine-type, sparteine-type, cytisine-type, and cytisine-like-type [17, 24]. Investigation of the seeds of *O. henryi* Prain led to the isolation of 13 alkaloids. Compounds **1**–**4** are the first example of ormosanine-type alkaloids with a methylpyrimidine ring that are abundant in the seeds. They have no polar groups and have little polarity. Compounds **1**, **2**,** 5** and **6** exist in the form of racemates. Pharmacological activity studies showed that cytisine (**12**) exhibited the most potent AChE inhibitory activity with an IC_50_ value of 6.851 ± 1.203 μM and the most excellent neuroprotective effects against A*β*_25-35_ induced PC12 cell damage, with an EC_50_ values of 7.99 μM. The new ormosanine-type alkaloid **3** exhibited moderate AChE inhibitory activity with an IC_50_ value of 141.5 ± 19.55 μM and significant neuroprotective effects against A*β*_25-35_ induced PC12 cell damage, with an EC_50_ values of 15.49 μM. This research enriched the structural diversity of alkaloids from the plants of the *Ormosia* genus and presented potent natural AChE inhibitors and neuroprotective compounds for further investigation.

## Supplementary Information

Additional file 1 (Details of AChE inhibitory and neuroprotective activity assays for the compounds, the measurement and calculation of the specific rotation for compounds **1** and **2**, and the spectroscopic data for compounds **1**−**4**, **7** and **8** are provided and available from the corresponding author upon reasonable request.)

## Data Availability

All data generated and analyzed during this study are included in this published article and its Additional file 1.
