# Supplementary material for: (+)-/(−)-Ormohenins A and B, two pairs of ormosanine-type enantiomers and their derivatives with neuroprotective activity from Ormosia henryi Prain
Source: Nat Prod Bioprospect. 2025 Aug 25;15(1):58. doi: 10.1007/s13659-025-00539-2 (PMC12378240; doi:10.1007/s13659-025-00539-2)
Supplement: Supplementary file 1 — Additional file 1 (Details of AChE inhibitory and neuroprotective activity assays for the compounds, the measurement and calculation of the specific rotation for compounds 1 and 2, and the spectroscopic data for compounds 1−4, 7 and 8 are provided and available from the corresponding author upon reasonable request.) [file 13659_2025_539_MOESM1_ESM.docx]

**Supporting Information for**

**(+)-/(-)-Ormohenins A and B, two pairs of ormosanine-type enantiomers and their derivatives with neuroprotective activity from *Ormosia henryi* Prain**

**Supporting Information List**

**Measurement and calculation of the specific rotation of ormohenin A (chiral HPLC fraction II) in methanol.**……..…………………………………………………………4

**Measurement and calculation of the specific rotation of ormohenin B (chiral HPLC fraction II) in methanol.**……..…………………………………………………………6

**ECD calculations of 4, 7, and 8**…………………………………………………………8

**AChE Inhibitory activity Assay.**……..………………………………………………13

**Neuroprotective activity Assay.**……..………………………………………………14

**Figure S1**. ^1^H NMR spectrum of **1** in methanol-*d*_4_……..…………………………17

**Figure S2**. ^13^C NMR spectrum of **1** in methanol-*d*_4_……………...……………….17

**Figure S3**. HSQC spectrum of **1** in methanol-*d*_4_…………………………………18

**Figure S4**. HMBC spectrum of **1** in methanol-*d*_4_…………….…………………….18

**Figure S5**. ^1^H-^1^H COSY spectrum of **1** in methanol-*d*_4_……….…………………….19

**Figure S6**. ROESY spectrum of **1** in methanol-*d*_4_…………………………………20

**Figure S7**. HRESIMS spectrum of **1** ………………………………….…………21

**Figure S8**. UV spectrum of **1** …………………………………………….…………22

**Figure S9**. IR spectrum of **1** …………………………………………….…………22

**Figure S10**. ^1^H NMR spectrum of **2** in methanol-*d*_4_……..…………………………23

**Figure S11**. ^13^C NMR spectrum of **2** in methanol-*d*_4_……………...……………….23

**Figure S12**. HSQC spectrum of **2** in methanol-*d*_4_…………………………………24

**Figure S13**. HMBC spectrum of **2** in methanol-*d*_4_…………….…………………….24

**Figure S14**. ^1^H-^1^H COSY spectrum of **2** in methanol-*d*_4_……….…………………25

**Figure S15**. ROESY spectrum of **2** in methanol-*d*_4_…………………………………26

**Figure S16**. HRESIMS spectrum of **2** ………………………………….…………27

**Figure S17**. UV spectrum of **2** ………………………………………….…………28

**Figure S18**. IR spectrum of **2** …………………………………………….…………28

**Figure S19**. ^1^H NMR spectrum of **3** in methanol-*d*_4_……..…………………………29

**Figure S20**. ^13^C NMR spectrum of **3** in methanol-*d*_4_……………...……………….29

**Figure S21**. HSQC spectrum of **3** in methanol-*d*_4_…………………………………30

**Figure S22**. HMBC spectrum of **3** in methanol-*d*_4_…………….…………………….30

**Figure S23**. ^1^H-^1^H COSY spectrum of **3** in methanol-*d*_4_……….…………………31

**Figure S24**. ROESY spectrum of **3** in methanol-*d*_4_…………………………………32

**Figure S25**. HRESIMS spectrum of **3** ………………………………….…………33

**Figure S26**. UV spectrum of **3** ………………………………………….…………34

**Figure S27**. IR spectrum of **3** ………………………………………….…………34

**Figure S28**. ^1^H NMR spectrum of **4** in methanol-*d*_4_……..…………………………35

**Figure S29**. ^13^C NMR spectrum of **4** in methanol-*d*_4_……………...……………….35

**Figure S30**. HSQC spectrum of **4** in methanol-*d*_4_…………………………………36

**Figure S31**. HMBC spectrum of **4** in methanol-*d*_4_…………….…………………….36

**Figure S32**. ^1^H-^1^H COSY spectrum of **4** in methanol-*d*_4_……….…………………37

**Figure S33**. ROESY spectrum of **4** in methanol-*d*_4_…………………………………38

**Figure S34**. HRESIMS spectrum of **4** ………………………………….…………39

**Figure S35**. UV spectrum of **4** ………………………………………….…………39

**Figure S36**. IR spectrum of **4** ………………………………………….…………40

**Figure S37**. ^1^H NMR spectrum of **7** in methanol-*d*_4_……..…………………………40

**Figure S38**. ^13^C NMR spectrum of **7** in methanol-*d*_4_……………...……………….41

**Figure S39**. HSQC spectrum of **7** in methanol-*d*_4_…………………………………41

**Figure S40**. HMBC spectrum of **7** in methanol-*d*_4_…………….…………………….42

**Figure S41**. ^1^H-^1^H COSY spectrum of **7** in methanol-*d*_4_………………………….42

**Figure S42**. ROESY spectrum of **7** in methanol-*d*_4_…………………………………43

**Figure S43**. HRESIMS spectrum of **7** ………………………………….…………44

**Figure S44**. UV spectrum of **7** ………………………………………….…………44

**Figure S45**. IR spectrum of **7** ………………………………………….…………45

**Figure S46**. ^1^H NMR spectrum of **8** in CDCl_3_….…………………………………45

**Figure S47**. ^13^C NMR spectrum of **8** in CDCl_3_…………………...……………….46

**Figure S48**. HSQC spectrum of **8** in CDCl_3_…………………………………………46

**Figure S49**. HMBC spectrum of **8** in CDCl_3_………………….…………………….47

**Figure S50**. ^1^H-^1^H COSY spectrum of **8** in CDCl_3_………….…………………….47

**Figure S51**. ROESY spectrum of **8** in CDCl_3_………………………………………48

**Figure S52**. HRESIMS spectrum of **8** ………………………………….…………48

**Figure S53**. IR spectrum of **8** …………………………………………….…………49

**Figure S54**. X-ray structure of **1** ………………………………………….…………50

**Table S9**. Crystal data and structure refinement for **1** …………………………51

**Figure S55**. X-ray structure of **2** ………………………………………….…………52

**Table S10**. Crystal data and structure refinement for **2** …………………………53

**Figure S56**. X-ray structure of **3** ………………………………………….…………54

**Table S11**. Crystal data and structure refinement for **3** …………………………55

**Measurement and** **calculation of the specific rotation of ormohenin A (chiral HPLC fraction II) in methanol.**

Optical rotation measurements were made with an Autopol VI automatic polarimeter (Rudolph Research Analytical). The sample of ormohenin A (chiral HPLC fraction II in Fig. 4) was run in CH_3_OH at a concentration of 1.09 mg/ml at 589 nm and 20ºC, giving an average observed rotation of −0.060 ± 0.001º (n = 20 replicated measurements), equal to a specific rotation of −55º.

To estimate the calculated specific rotation of ormohenin A, the absolute configuration of **1** on the left side shown in Fig. 1 was submitted to conformational analysis using Spartan´08 (Shao et al. 2006) and MMFF94. Four relevant conformers were found in an energy window of 12.5 KJ/mol. The four structures were optimized in *Gaussian´09, Revision A.02* (Schlegel et al. 2016) using B3LYP and a solvent model (IEFPCM) for methanol. The obtained total energies are:

1. −1025.157813 hartree
2. −1025.148575 hartree
3. −1025.13476 hartree
4. −1025.142348 hartree

At 20 °C this translates to a Boltzmann-population of:

1. 99.9944%
2. 0.0056%
3. < 0.0001%
4. < 0.0001%

The optical rotation values at 589 nm were calculated at the B3LYP/6-311++G(2d,p) level of theory. The predicted values are:

1. −48.31
2. −17.31
3. −50.31
4. −49.33

Using the calculated Boltzmann-populations the predicted averaged optical rotation of structure (on left side structure of **1** in Fig. 1) is −48.3º.

Conformer 1


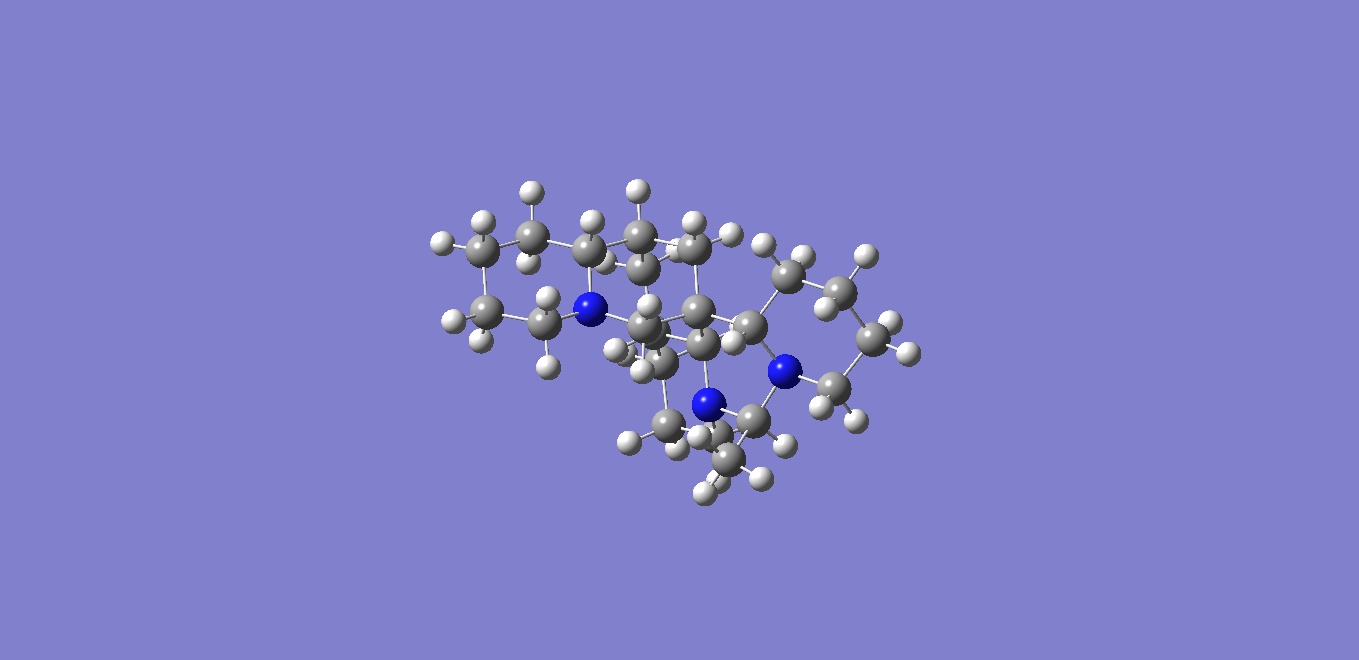


Conformer 2


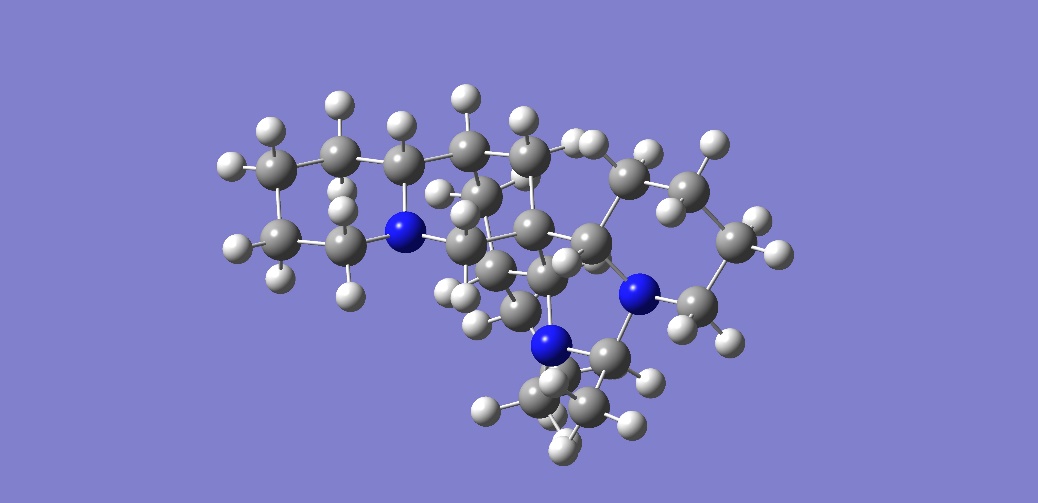


Conformer 3


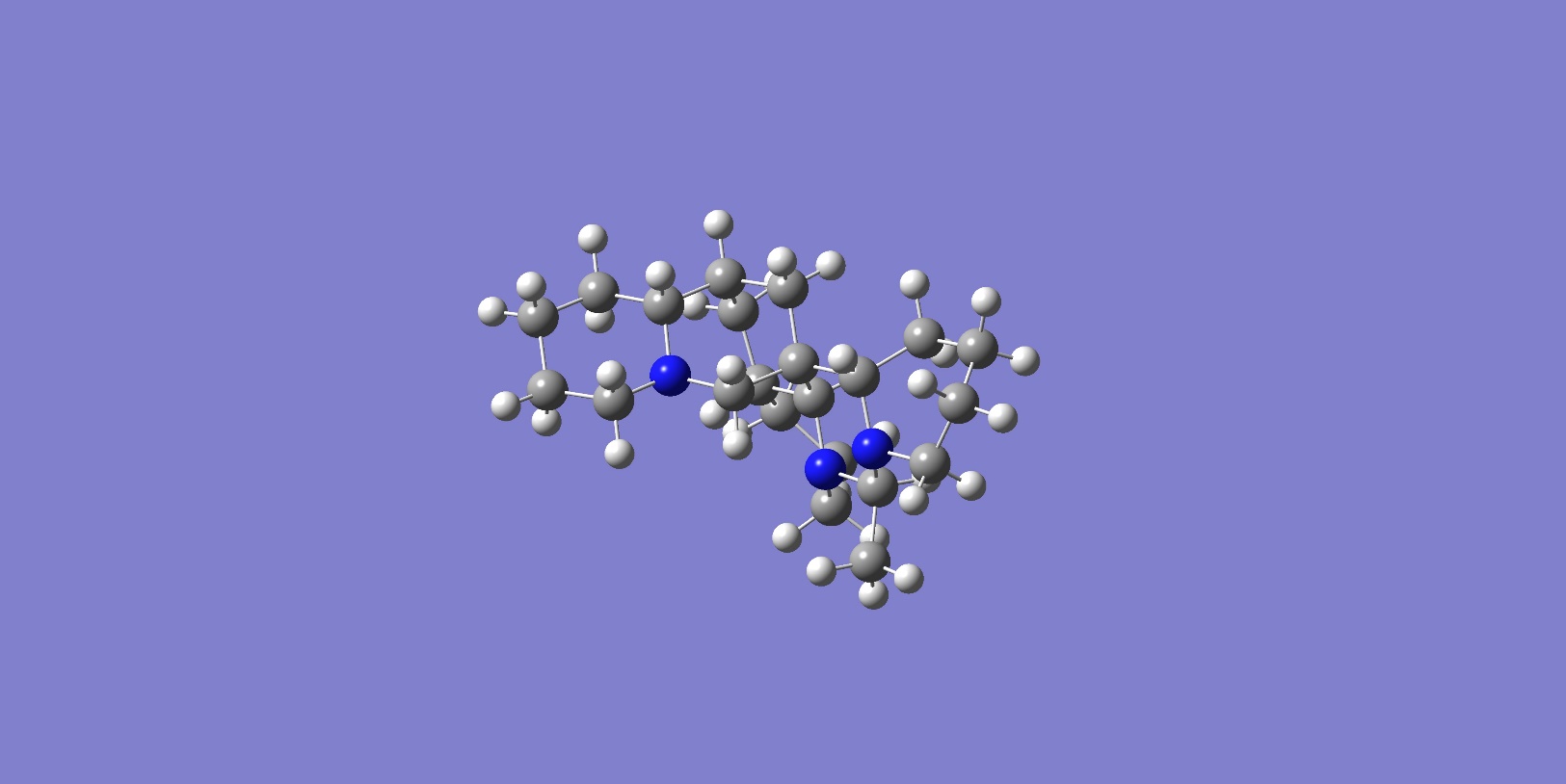


Conformer 4


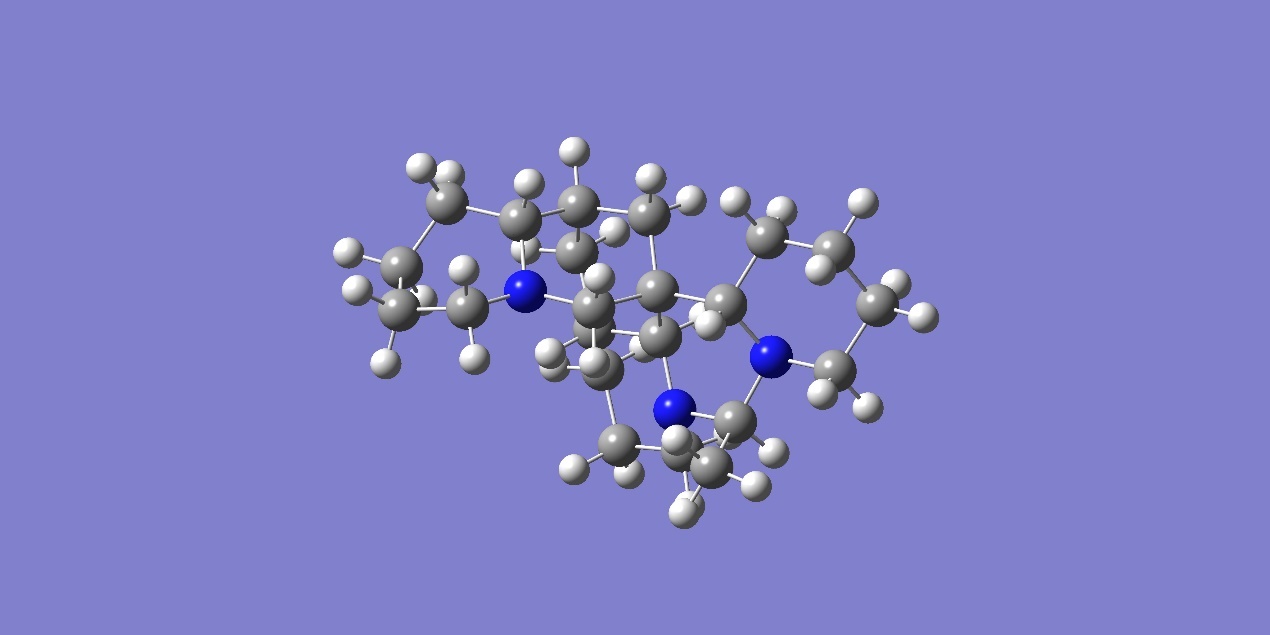


**Measurement and calculation of the specific rotation of ormohenin B (chiral HPLC fraction II) in methanol.**

Optical rotation measurements were made with an Autopol VI automatic polarimeter (Rudolph Research Analytical). The sample of ormohenin B (chiral HPLC fraction II in Fig. 5) was run in CH_3_OH at a concentration of 1.03 mg/ml at 589 nm and 17ºC, giving an average observed rotation of −0.012 ± 0.001º (n = 20 replicated measurements), equal to a specific rotation of −11.6º.

To estimate the calculated specific rotation of ormohenin B, the absolute configuration of **2** on the left side shown in Fig. 1 was submitted to conformational analysis using Spartan´08 (Shao et al. 2006) and MMFF94. Four relevant conformers were found in an energy window of 12.5 KJ/mol. The four structures were optimized in *Gaussian´09, Revision A.02* (Schlegel et al. 2016) using B3LYP and a solvent model (IEFPCM) for methanol. The obtained total energies are:

1. − 1025.133735 hartree
2. − 1025.142346 hartree
3. − 1025.125411 hartree
4. − 1025.127042 hartree

At 20 °C this translates to a Boltzmann-population of:

1. 0.0109%
2. 99.989%
3. < 0.0001%
4. < 0.0001%

The optical rotation values at 589 nm were calculated at the B3LYP/6-311++G(2d,p) level of theory. The predicted values are:

1. +2.68
2. −24.84
3. −68.66
4. +5.94

Using the calculated Boltzmann-populations the predicted averaged optical rotation of structure (on left side structure of **1** in Fig. 1) is −24.8º.

Conformer 1


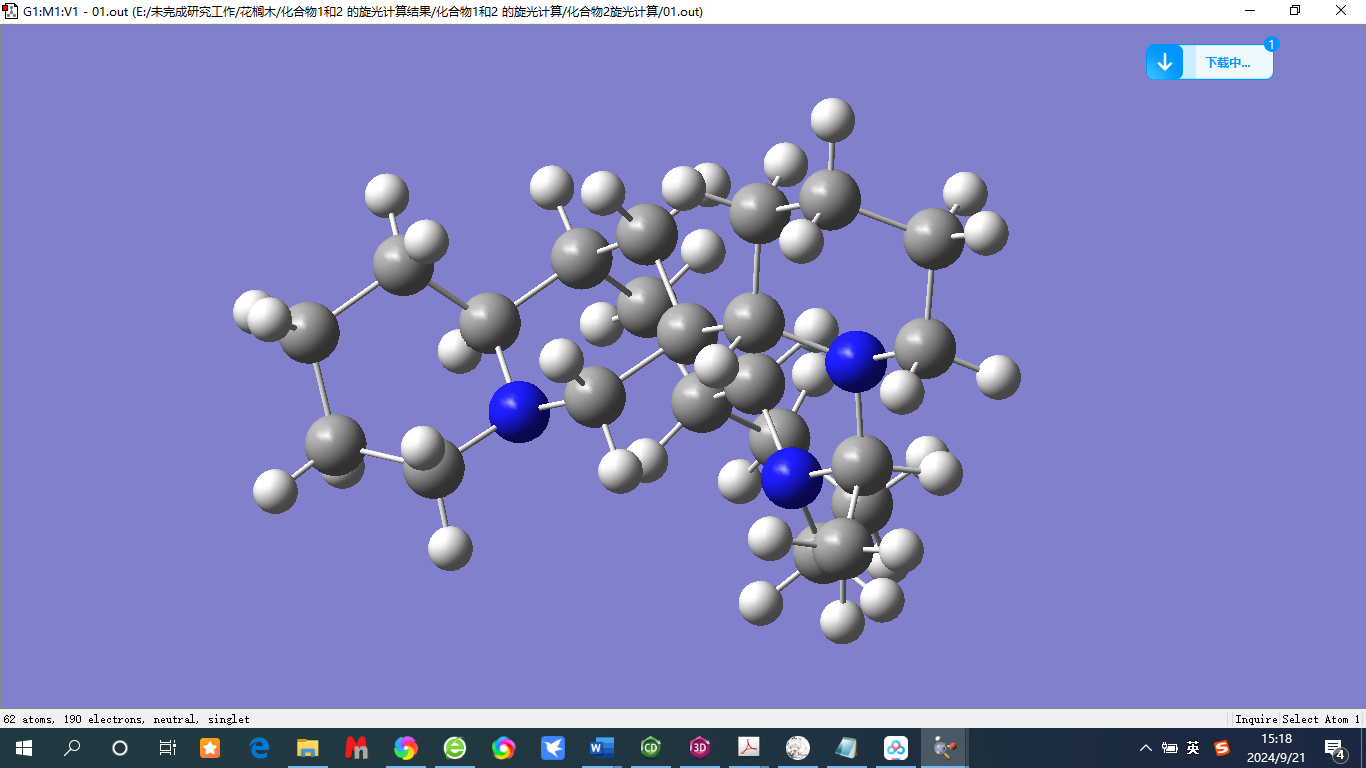


Conformer 2


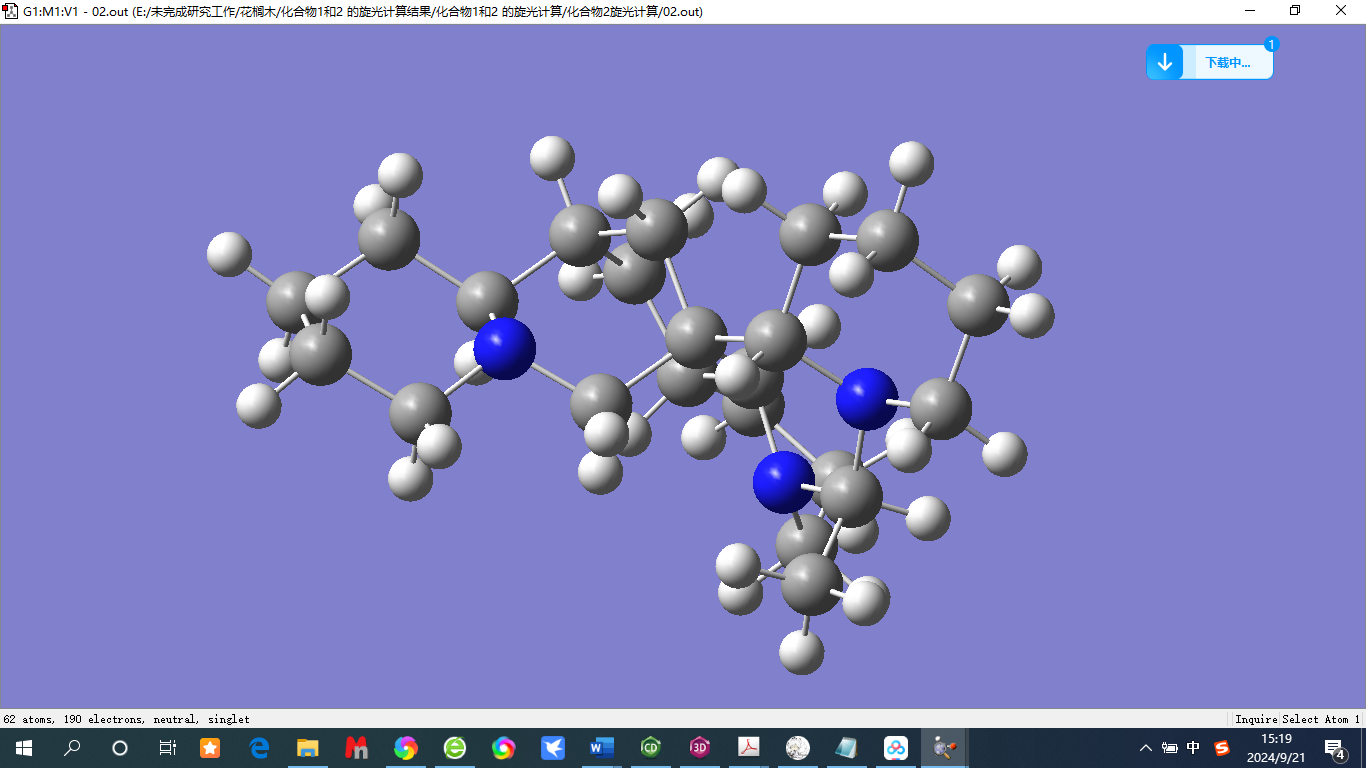


Conformer 3


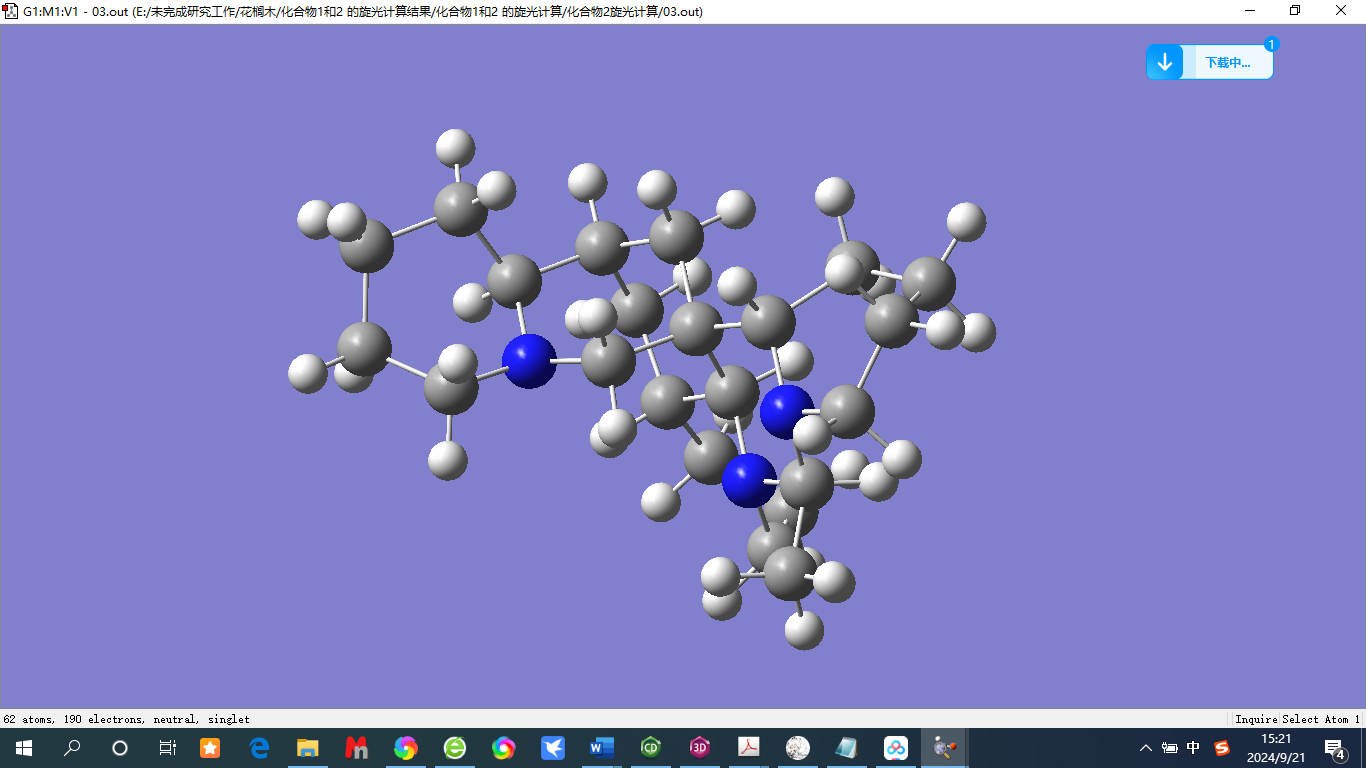


Conformer 4


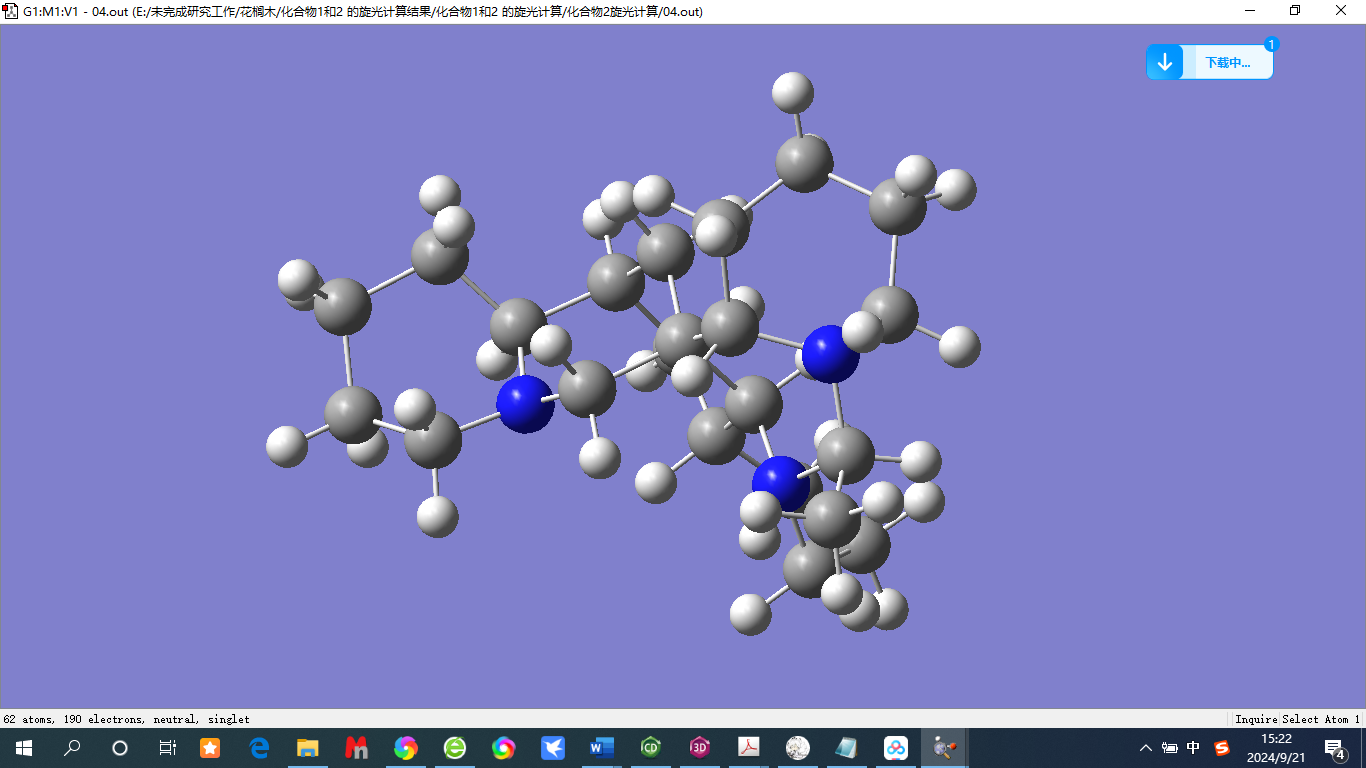


**ECD Calculations of 4, 7, and 8**

Conformational search for compounds was accomplished by the Spartan’s 14.0 (Wavefunction Inc., Irvine, CA, USA) software with the MMFF94 force field. And the suitable conformers were screened based on the energy window within 3 kcal mol^−1^. The selected conformers were then optimized at the B3LYP/6-31G(d, p) level in the *Gaussian 09* program package (Frisch et al. 2013). After that, ECD calculation was conducted using time-dependent density functional theory (TDDFT) at the B3LYP/6-31G(d, p) level in MeOH (IEPCM solvent model). Finally, the ECD curves of different conformers were simulated using SpecDis 1.51 based on the Boltzmann weighing of each conformer (Bruhn et al. 2013).

**Table S1. Energy analysis for 4.**

| conformer | Gibbs free energy (298.15 K) | | |
| --- | --- | --- | --- |
|  | G (Hartree) | ΔE (Hartree) | Population (%) |
| **4a** | -1022.6398 | 0 | 97.34 |
| **4b** | -1022.6312 | 0.0034 | 2.66 |
| **4c** | -1022.6278 | 0.0120 | 0 |


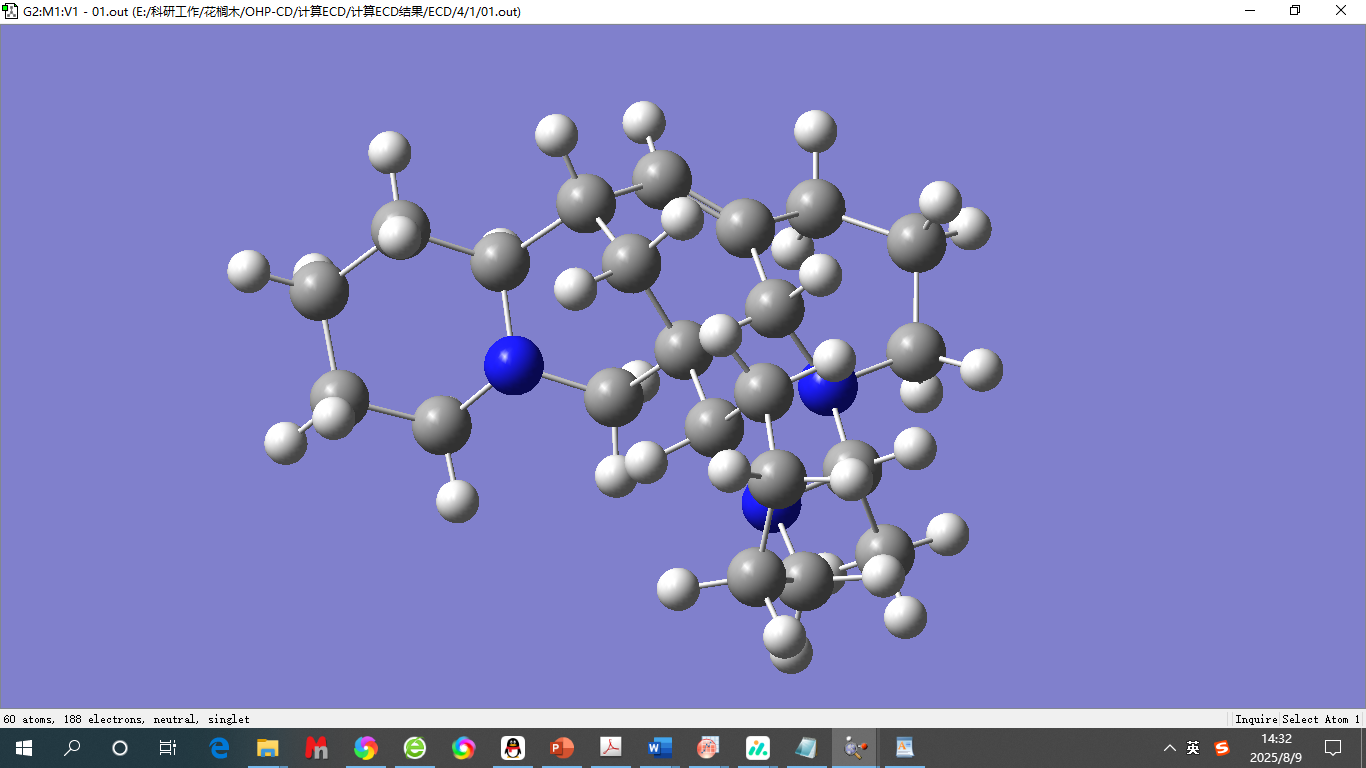

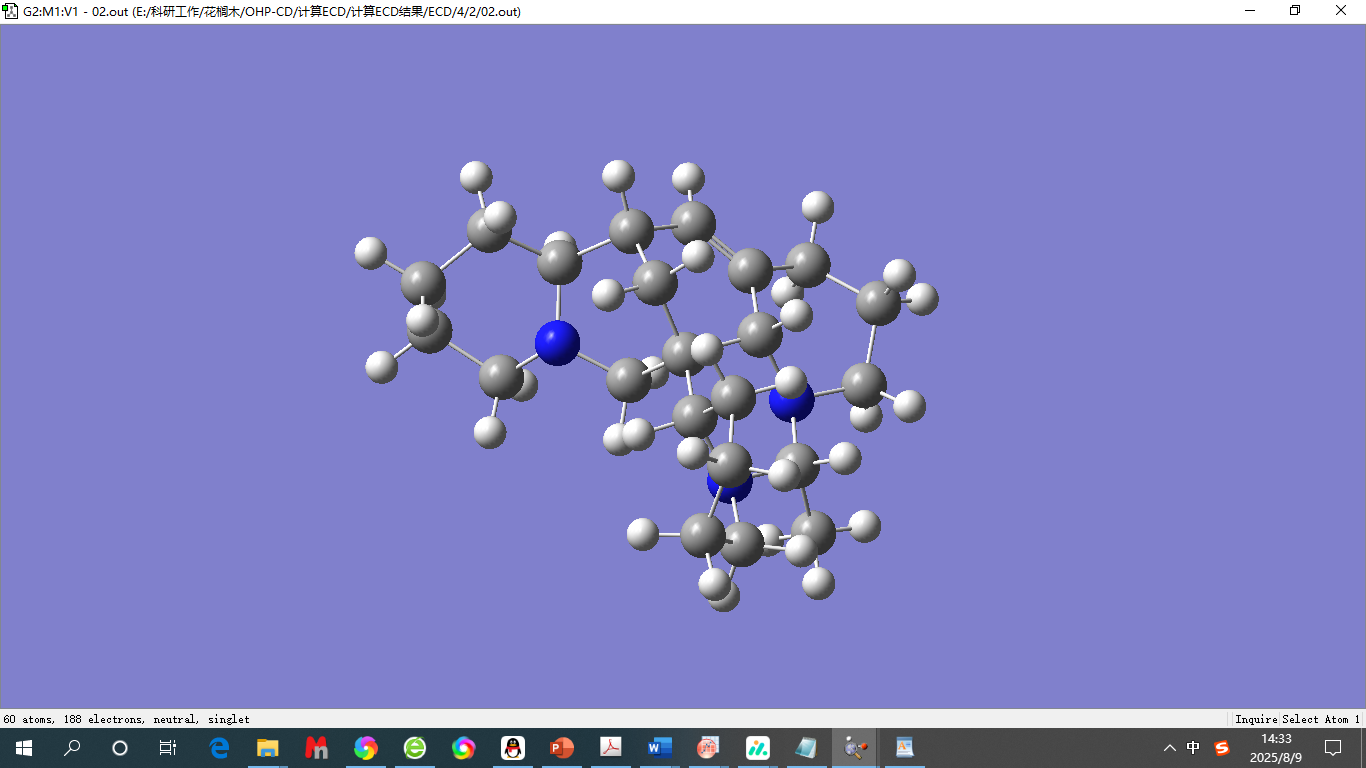

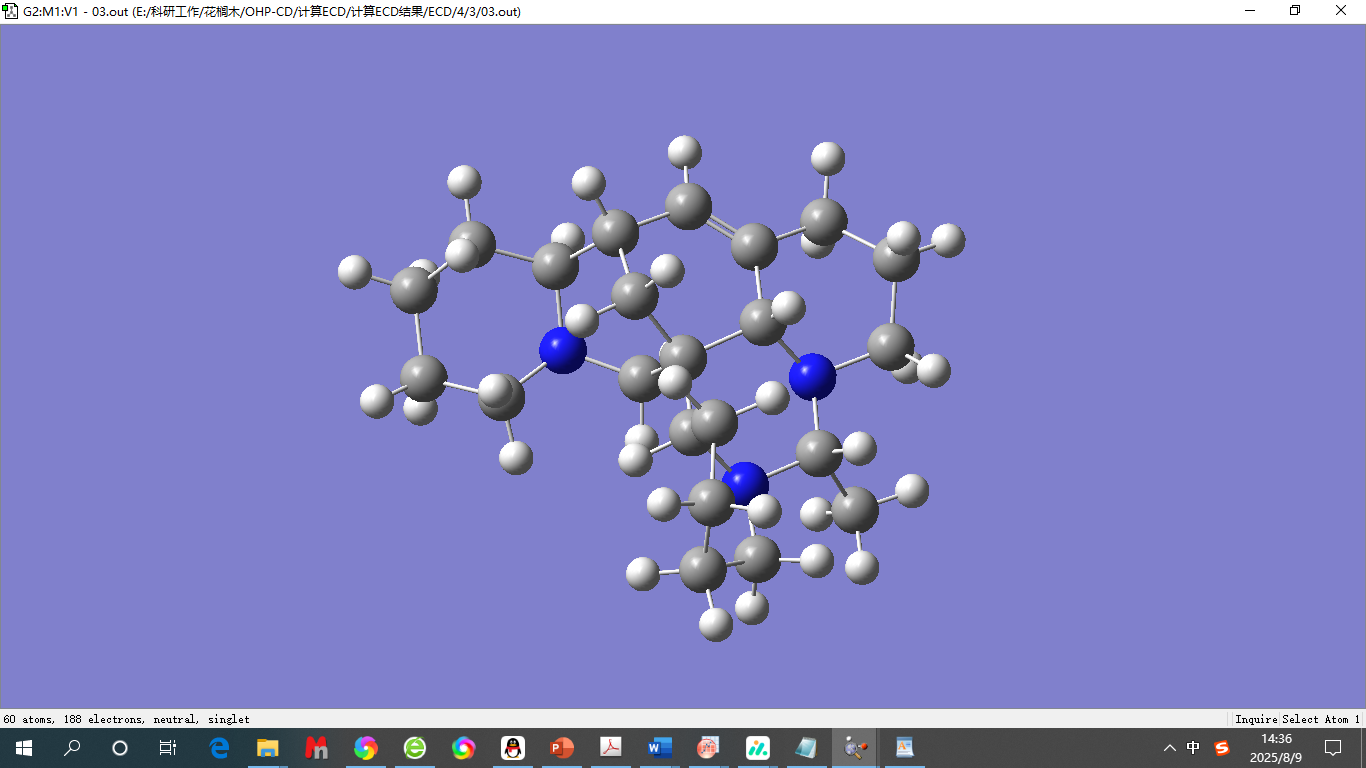


**4a 4b 4c**

**Figure Q1**. B3LYP (PCM, methanol)/6-31G(d,p) optimized lowest energy conformers for **4**.

**Table S2. Calculated ECD Data for 4.**

|  | **4a** | | **4b** | | **4c** | | |
| --- | --- | --- | --- | --- | --- | --- | --- |
| State | Excitation energies(eV) | Rotatory | Excitation energies(eV) | Rotatory | Excitation energies(eV) | Rotatory | |
|  |  | Strengths* |  | Strengths* |  | Strengths* | |
| 1 | 3.8107 | -2.4096 | 3.7595 | 5.7116 | 3.6121 | | 15.3390 |
| 2 | 3.9326 | 18.0311 | 3.8433 | 14.8524 | 3.6556 | | -6.7136 |
| 3 | 3.9420 | 2.1441 | 3.8861 | 3.9234 | 3.8667 | | 2.3185 |
| 4 | 4.0710 | -4.8951 | 3.9907 | -3.3109 | 3.9062 | | -2.1061 |
| 5 | 4.1457 | 20.1925 | 4.0933 | 12.5855 | 3.9451 | | 0.4018 |
| 6 | 4.2045 | -4.1282 | 4.1556 | 1.3474 | 3.9701 | | 0.7079 |
| 7 | 4.2361 | 5.8149 | 4.1950 | 1.9964 | 4.0620 | | -11.9998 |
| 8 | 4.2726 | -15.7559 | 4.2096 | -1.5582 | 4.1764 | | -2.4911 |
| 9 | 4.2943 | -2.7518 | 4.2405 | 15.3662 | 4.1955 | | 2.7904 |
| 10 | 4.3366 | 1.2992 | 4.2602 | -6.3881 | 4.2018 | | 3.4996 |
| 11 | 4.3701 | -1.7630 | 4.3201 | -1.8712 | 4.2101 | | 29.8355 |
| 12 | 4.4238 | -1.6276 | 4.3484 | 6.7201 | 4.2589 | | -0.0875 |
| 13 | 4.4520 | 8.2568 | 4.4073 | 2.4194 | 4.3098 | | 2.0510 |
| 14 | 4.5159 | -10.1389 | 4.4897 | -3.9882 | 4.3712 | | -2.1797 |
| 15 | 4.5633 | -9.9515 | 4.5174 | -5.8736 | 4.3844 | | 1.2310 |
| 16 | 4.5823 | -10.4219 | 4.5286 | -6.7139 | 4.4269 | | -1.0448 |
| 17 | 4.6017 | 1.0872 | 4.5515 | 6.6562 | 4.4822 | | -7.2122 |
| 18 | 4.6286 | -9.2114 | 4.5789 | -7.3529 | 4.4863 | | -0.4730 |
| 19 | 4.6464 | 0.5565 | 4.6015 | 5.0174 | 4.5149 | | -16.5515 |
| 20 | 4.7111 | 4.3010 | 4.6608 | 5.0174 | 4.5332 | | 0.3978 |
| 21 | 4.7156 | -0.5203 | 4.6649 | 5.0391 | 4.6004 | | 1.9531 |
| 22 | 4.7329 | 1.2446 | 4.6679 | -2.5459 | 4.6199 | | 2.0976 |
| 23 | 4.7818 | -4.9069 | 4.7294 | -8.3348 | 4.6909 | | -1.4356 |
| 24 | 4.8428 | 4.5219 | 4.7753 | 0.2845 | 4.7104 | | 6.8761 |
| 25 | 4.8729 | 14.1176 | 4.8355 | -0.4002 | 4.7592 | | 1.7090 |
| 26 | 4.9126 | -2.2501 | 4.8398 | 8.4420 | 4.7720 | | 6.6764 |
| 27 | 4.9346 | 3.7184 | 4.9189 | 4.5943 | 4.7720 | | -7.5613 |
| 28 | 4.9652 | -5.1942 | 4.9213 | -2.2943 | 4.8295 | | 0.4236 |
| 29 | 5.0217 | -6.6933 | 4.9734 | -2.3056 | 4.8437 | | 3.1866 |
| 30 | 5.0249 | 1.9749 | 4.9826 | -6.5067 | 4.8826 | | 4.1017 |

* R(velocity) 10**-40 erg-esu-cm/Gauss

**Table S3. Energy analysis for 7.**

| conformer | Gibbs free energy (298.15 K) | | |
| --- | --- | --- | --- |
|  | G (Hartree) | ΔE (Hartree) | Population (%) |
| **7a** | -768.8706 | 0 | 99.998 |
| **7b** | -768.8604 | 0.0102 | 0.002 |


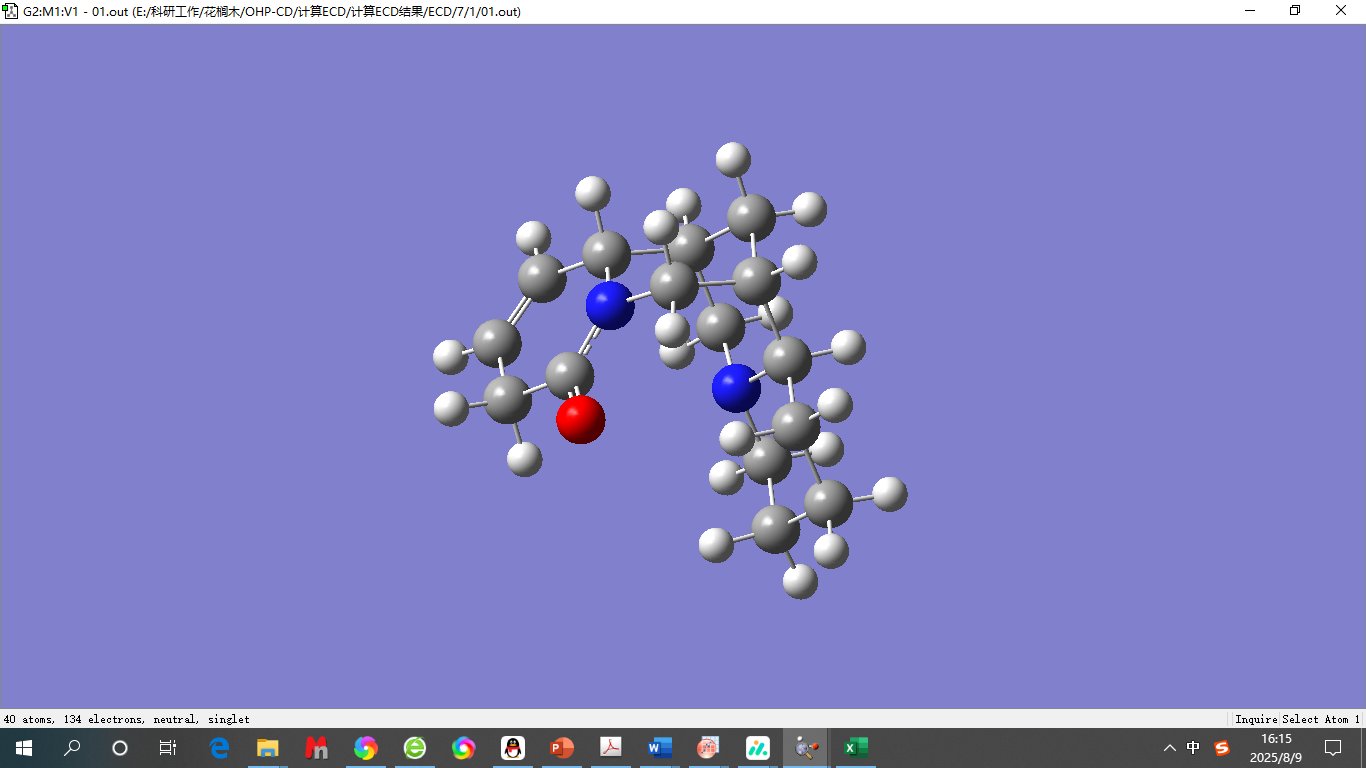

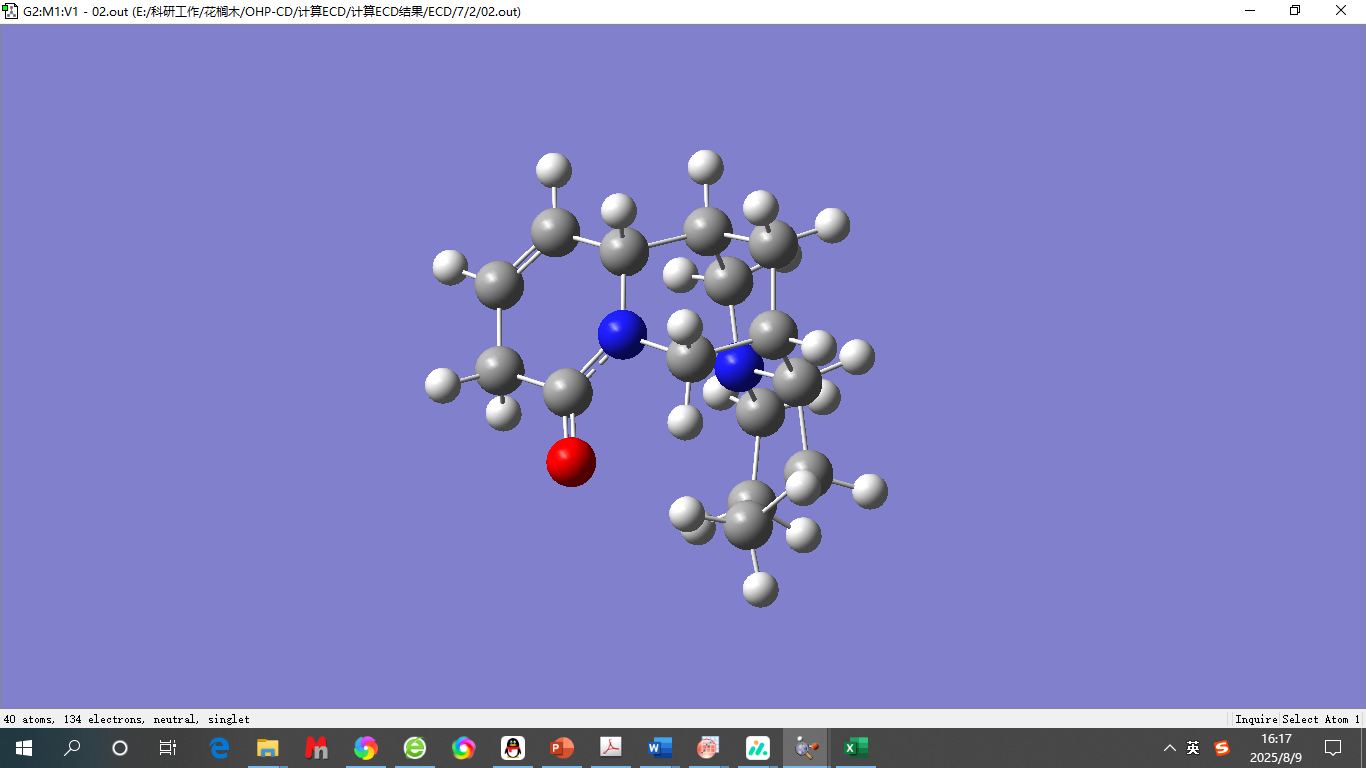


**7a 7b**

**Figure Q2**. B3LYP (PCM, methanol)/6-31G(d,p) optimized lowest energy conformers for **7**.

**Table S4. Calculated ECD Data for 7.**

|  | **7a** | | **7b** | |
| --- | --- | --- | --- | --- |
| State | Excitation energies(eV) | Rotatory | Excitation energies(eV) | Rotatory |
|  |  | Strengths* |  | Strengths* |
| 1 | 3.8727 | 2.6925 | 3.7923 | 1.3731 |
| 2 | 4.0602 | 7.0646 | 3.9650 | 3.5678 |
| 3 | 4.3534 | 9.9249 | 4.2648 | 2.0090 |
| 4 | 4.4468 | 1.9402 | 4.3736 | -9.6069 |
| 5 | 4.4929 | -0.8690 | 4.4043 | 13.1223 |
| 6 | 4.5989 | -3.2433 | 4.5401 | 0.5265 |
| 7 | 4.9578 | -10.9840 | 4.8946 | -14.1057 |
| 8 | 4.9698 | 0.3042 | 4.9066 | -4.4601 |
| 9 | 4.9799 | -8.8719 | 4.9643 | 6.4542 |
| 10 | 5.0285 | 0.9863 | 4.9868 | -3.6019 |
| 11 | 5.0868 | -9.6290 | 5.0743 | 3.9870 |
| 12 | 5.1059 | -9.0104 | 5.0891 | -20.0537 |
| 13 | 5.1906 | 4.5003 | 5.1121 | -7.7514 |
| 14 | 5.2129 | -0.3615 | 5.1230 | 37.8759 |
| 15 | 5.2324 | 19.3931 | 5.1745 | -8.8104 |
| 16 | 5.4396 | -19.1692 | 5.4393 | -15.2470 |
| 17 | 5.4742 | 7.9308 | 5.4681 | -6.7809 |
| 18 | 5.5170 | 0.1547 | 5.4983 | -2.2954 |
| 19 | 5.5210 | -7.9525 | 5.5131 | -0.9888 |
| 20 | 5.5546 | -11.1714 | 5.5397 | -2.3967 |
| 21 | 5.5753 | 16.8492 | 5.5499 | 14.3642 |
| 22 | 5.6167 | -19.1435 | 5.5593 | 16.6956 |
| 23 | 5.6281 | 4.4562 | 5.5879 | -7.3203 |
| 24 | 5.6323 | -5.8108 | 5.6194 | -0.4601 |
| 25 | 5.6677 | 35.7855 | 5.6502 | 18.8438 |
| 26 | 5.6995 | -7.3745 | 5.6730 | -3.2823 |
| 27 | 5.7374 | -0.0158 | 5.6925 | -11.6765 |
| 28 | 5.7706 | -1.5099 | 5.6969 | -0.4807 |
| 29 | 5.8158 | -0.2518 | 5.7251 | -4.5800 |
| 30 | 5.9377 | -17.8635 | 5.8418 | -2.6648 |

**Table S5. Energy analysis for 8.**

| conformer | Gibbs free energy (298.15 K) | | |
| --- | --- | --- | --- |
|  | G (Hartree) | ΔE (Hartree) | Population (%) |
| **8a** | -768.8706 | 0 | 100 |


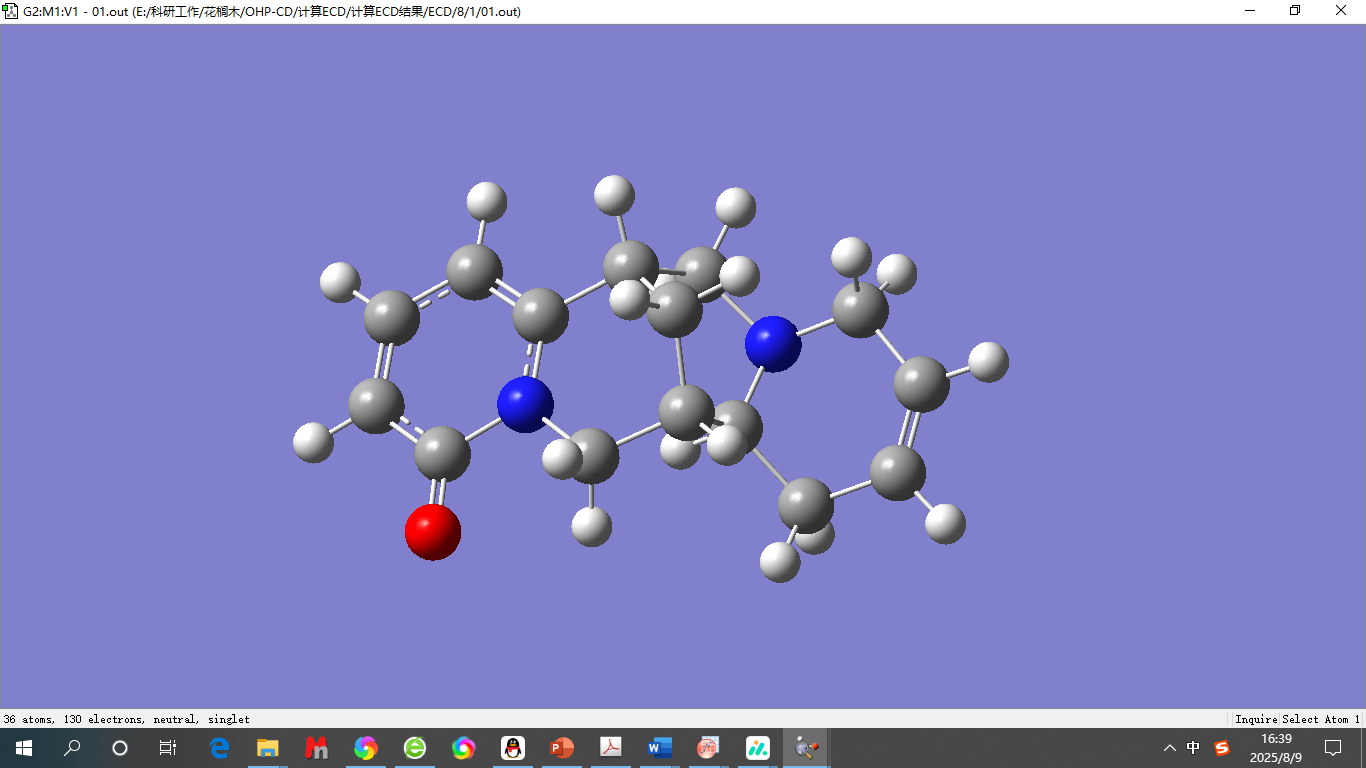


**8a**

**Figure Q3**. B3LYP (PCM, methanol)/6-31G(d,p) optimized lowest energy conformers for **8**.

**Table S6. Calculated ECD Data for 8.**

|  | **8a** | |
| --- | --- | --- |
| State | Excitation energies(eV) | Rotatory |
|  |  | Strengths* |
| 1 | 2.8911 | 2.5858 |
| 2 | 3.9088 | -13.3001 |
| 3 | 4.0362 | -6.8887 |
| 4 | 4.0604 | 3.9270 |
| 5 | 4.0809 | 0.4211 |
| 6 | 4.1405 | 5.6101 |
| 7 | 4.2488 | 7.9692 |
| 8 | 4.5027 | -3.1984 |
| 9 | 4.5800 | -5.9019 |
| 10 | 4.6305 | -1.9614 |
| 11 | 4.6406 | 2.2709 |
| 12 | 4.8238 | 2.4220 |
| 13 | 4.8839 | -2.2868 |
| 14 | 5.0514 | 4.4010 |
| 15 | 5.0884 | -7.8941 |
| 16 | 5.1327 | 10.3975 |
| 17 | 5.1619 | 10.3652 |
| 18 | 5.1865 | 8.3119 |
| 19 | 5.2317 | -33.5637 |
| 20 | 5.2484 | -1.9019 |
| 21 | 5.2484 | 3.8204 |
| 22 | 5.2792 | 2.8950 |
| 23 | 5.3220 | 9.6836 |
| 24 | 5.3446 | -10.8576 |
| 25 | 5.4130 | -4.3001 |
| 26 | 5.4702 | -3.9051 |
| 27 | 5.4743 | -3.0549 |
| 28 | 5.4936 | -1.8897 |
| 29 | 5.4968 | -5.3475 |
| 30 | 5.5290 | -1.0760 |

**REFERENCES:**

[1] Shao Y, Molnar LF, Jung Y, Kussmann J, Ochsenfeld C, Brown ST, et al. Advances in methods and algorithms in a modern quantum chemistry program package. Phys Chem Chem Phys. 2006, 8(27):3172-91. doi: 10.1039/b517914a.

[2] Schlegel H, Scuseria G, Robb M, Cheeseman J, Scalmani G, Barone V, et al. Gaussian 09, Revision A.02. Chemical Science, 2016.

[3] Frisch MJ, Trucks GW, Schlegel HB, Scuseria GE, Robb MA, Cheeseman JR, et al. Gaussian 09, Revision D.01, Gaussian, Inc., Wallingford CT, 2013.

[4] Bruhn T, Schaumlöffel A, Hemberger Y, Bringmann G. SpecDis: quantifying the comparison of calculated and experimental electronic circular dichroism spectra. Chirality, 2013, 25: 243-249.

*AChE Inhibitory activity Assay*

The inhibitory activity on acetylcholinesterase (AChE) of compounds **1**−**12** were evaluated by improved Ellman's method. The compounds were prepared in DMSO and diluted in buffer solution to different concentrations. In a 96-well plate, 140 μL of PBS (pH = 8) buffer, 20 μL of all the sample solutions were transferred in each well, and 20 μL of 0.2 U/mL AChE solution were added. The mixture was incubated at 37°C for 10 minutes. Subsequently, 10 μL of 3 mM 5,5'-dithiobis-(2-nitrobenzoic acid) (DTNB) and 10 μL of 15 mM acetylthiocholine (ATCl) were added, followed by reaction at 37 °C for 20 minutes. The absorbance was measured at 405 nm. Each experimental group was set up with three replicate wells, and huperzine A was used as the positive control. The inhibition rate (%) was calculated as follows: Inhibition rate(%)=[(A1-A2)-(A3-A4)]/(A1-A2)×100%

A1: Blank control group (with enzyme, no inhibitor)

A2: Blank background group (no enzyme, no inhibitor)

A3: Experimental group (with enzyme and inhibitor)

A4: Experimental background group (no enzyme, with inhibitor)

*Neuroprotective activity* *Assay*

*Cell culture.* PC12 cells were cultured in RPMI 1640 medium supplemented with 10% fetal bovine serum and 1% penicillin/streptomycin. The cells were maintained at 37°C in a humidified atmosphere containing 5% CO_2_. The culture medium was replaced every three days, and the cells were passaged when they reached 70% confluency.

*Preparation of Aβ_25-35_***_._** A*β*_25-35_ was dissolved in sterile double-distilled water to prepare a 2 mM stock solution. The solution was then incubated at 37°C for 7 days to induce aggregation.

*MTT assay for cell viability.* PC12 cells were divided into four groups: normal control, model, positive control (Resveratrol), and isolates intervention groups (1, 10, 20, 40, 60, 80, 100 μmol/L). The cells were seeded in 96-well plates at an appropriate density with 200 μL medium per well and were cultured for 24 hours. The positive control and intervention groups were pretreated with different concentrations of drug-containing medium for 2 hours, after which 2 μL of Aβ_25-35_ was added to each well of the model, positive control, and intervention groups for 24 hours. After the treatment, 20 μL of 5 mg/L MTT solution was added to each well and incubated for 4 hours. The medium was then removed, and 150 μL of DMSO was added to each well. The plate was shaken until the formazan product was completely dissolved. The absorbance was measured at 570 nm using a spectrophotometer.

The viability of PC12 cells and Aβ_25-35_-induced PC12 cells treated with resveratrol and twelve compounds are presented as below.

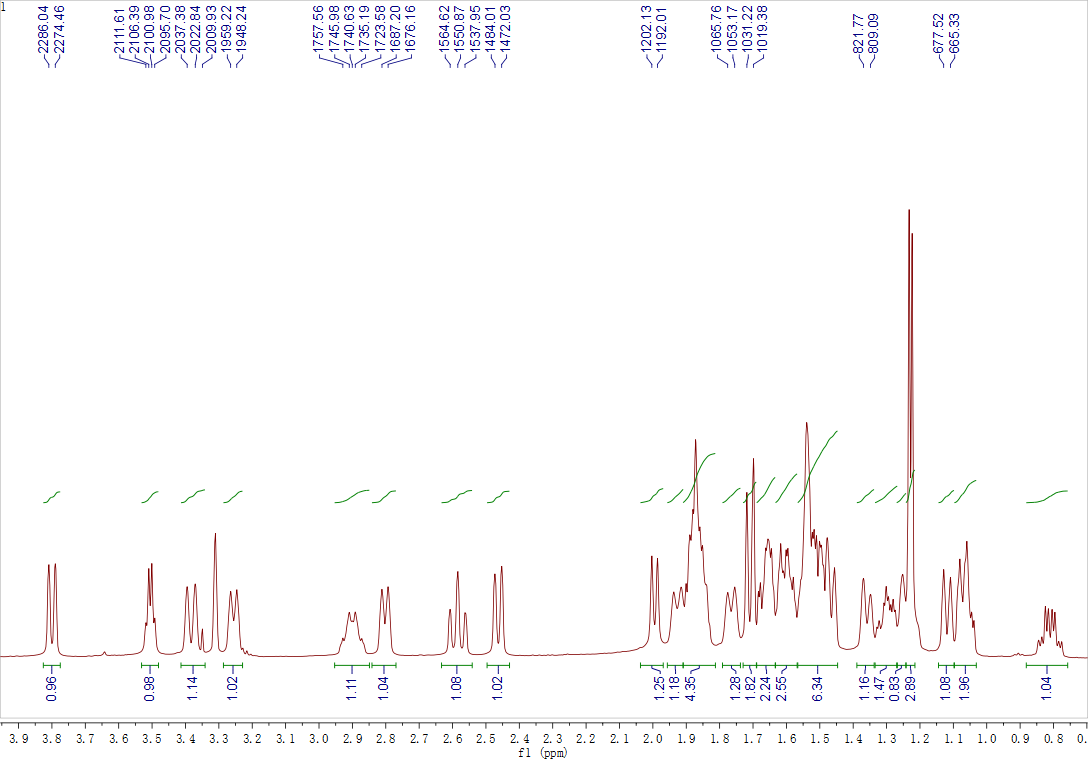


Figure S1. The ^1^H NMR spectrum of **1** in methanol-*d*_4_.


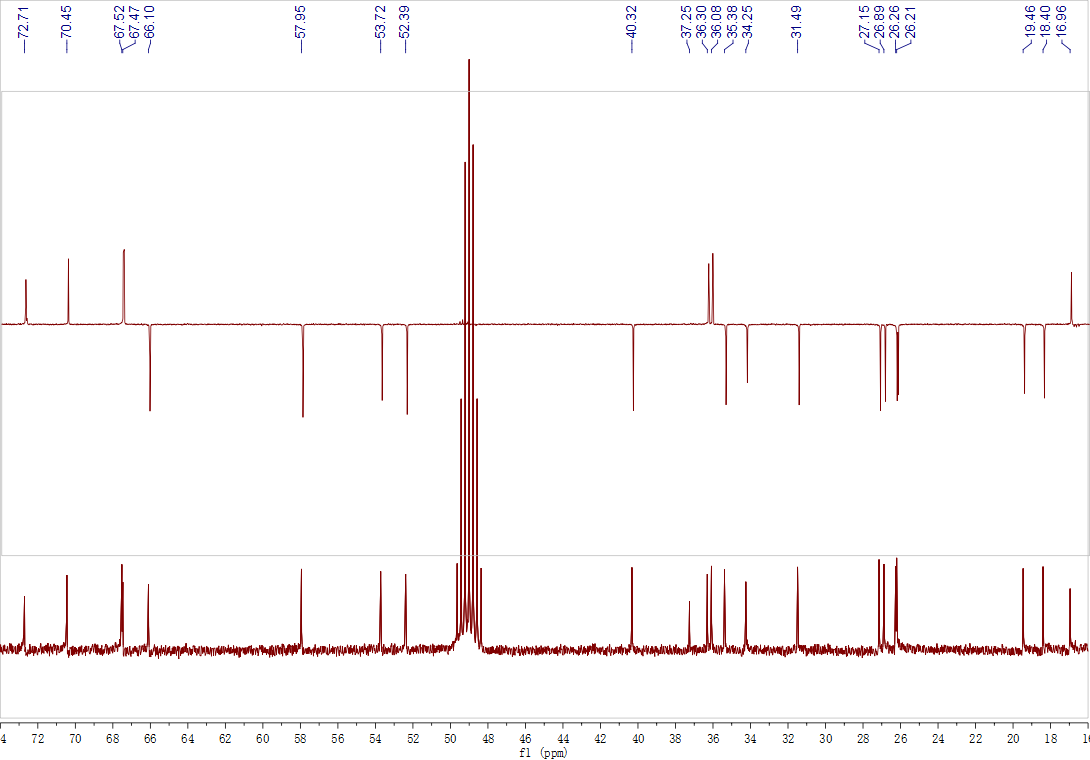


Figure S2. The ^13^C NMR spectrum of **1** in methanol-*d*_4_.


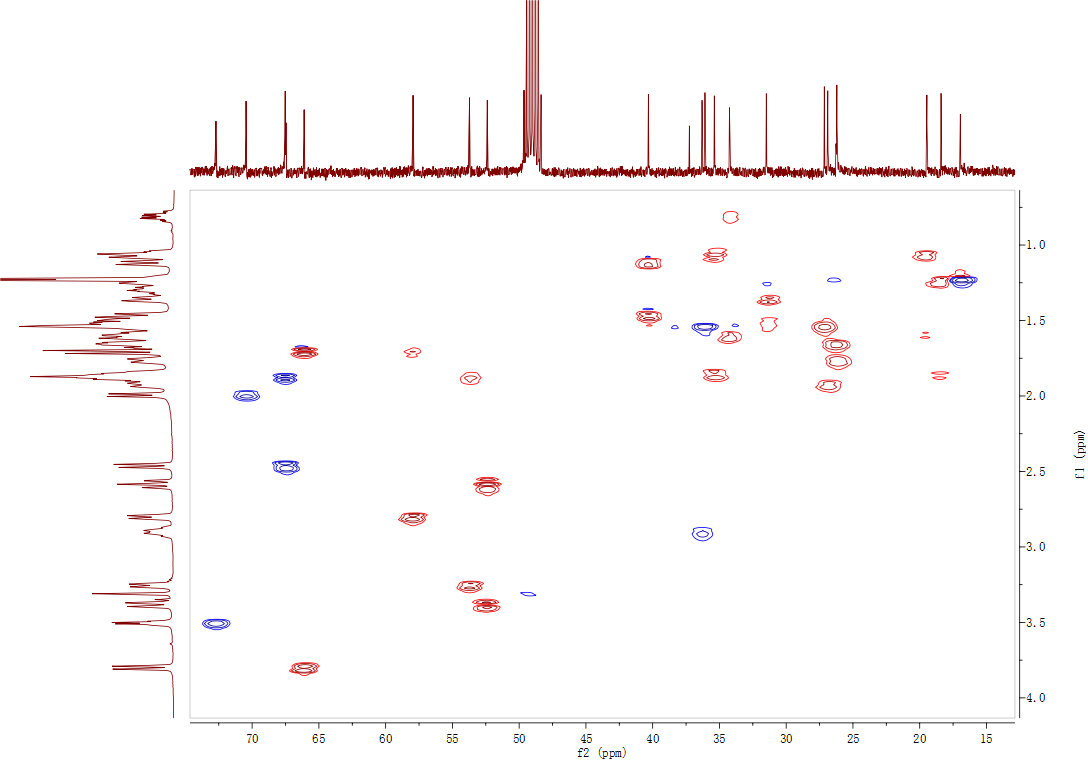


Figure S3. The HSQC spectrum of **1** in methanol-*d*_4_.


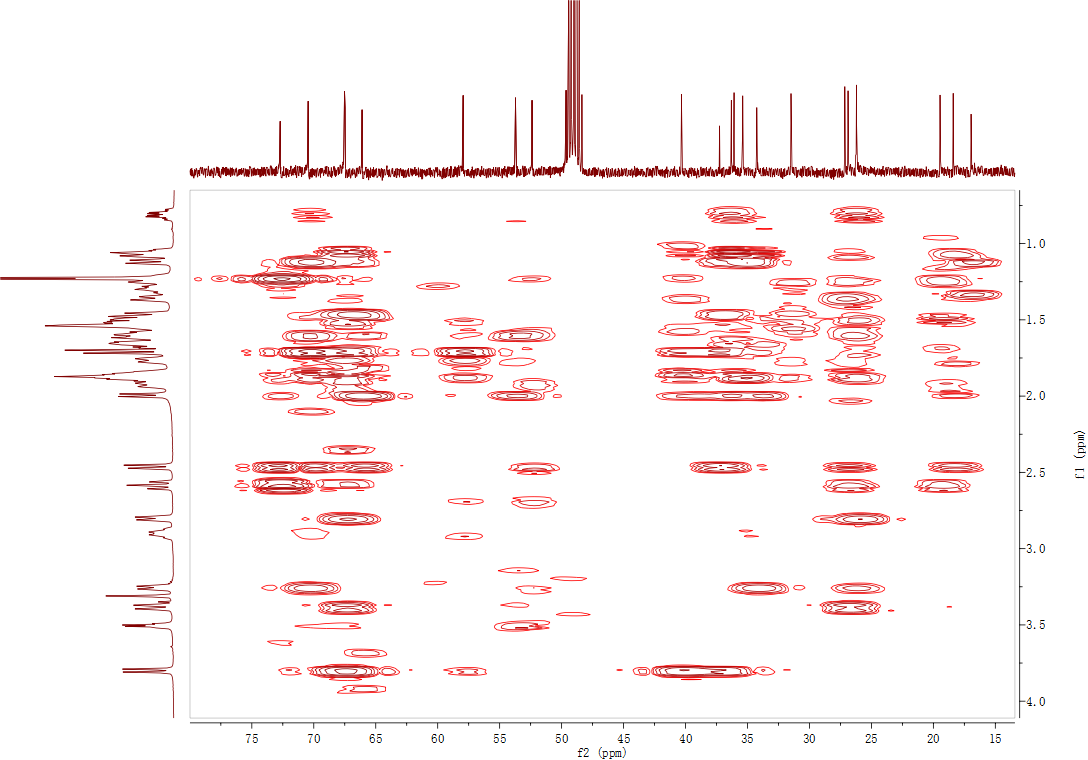


Figure S4. The HMBC spectrum of **1** in methanol-*d*_4_.


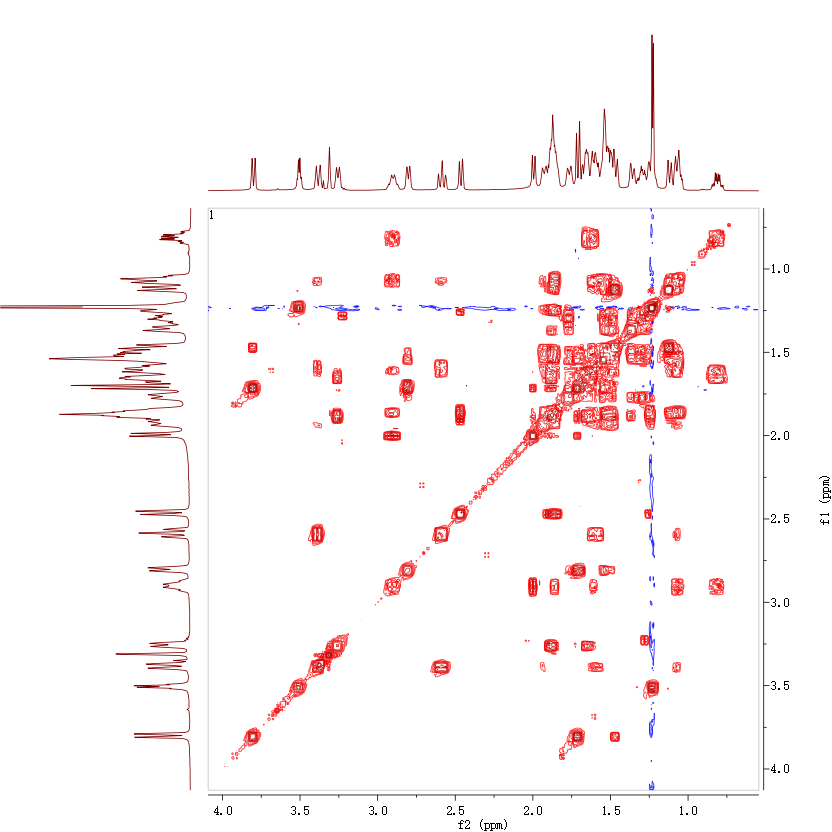


Figure S5. The ^1^H-^1^H COSY spectrum of **1** in methanol-*d*_4_.


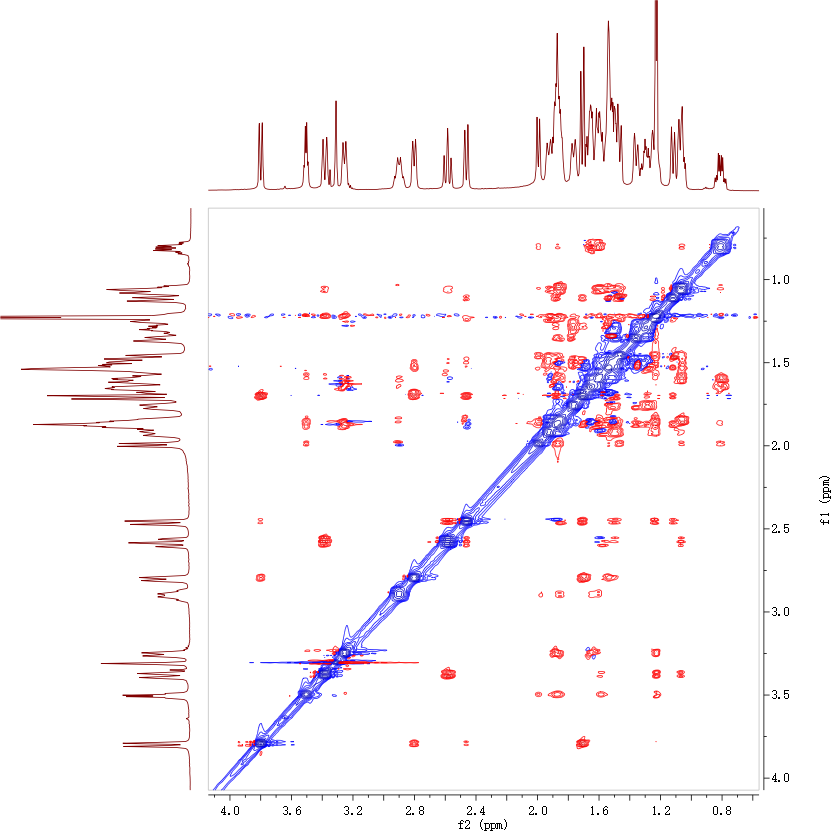


Figure S6. The ROESY spectrum of **1** in methanol-*d*_4_.


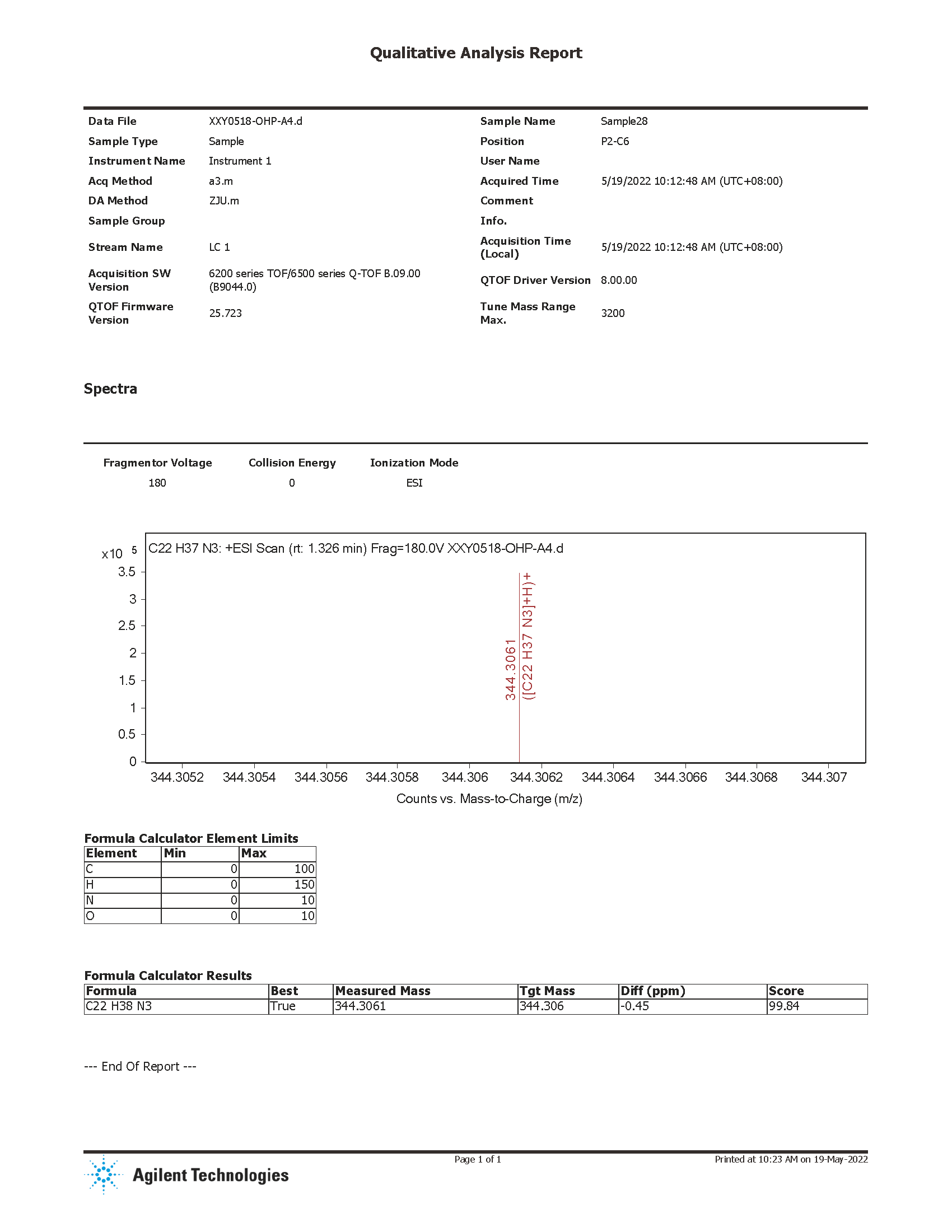


Figure S7. The HRESIMS data of **1**.


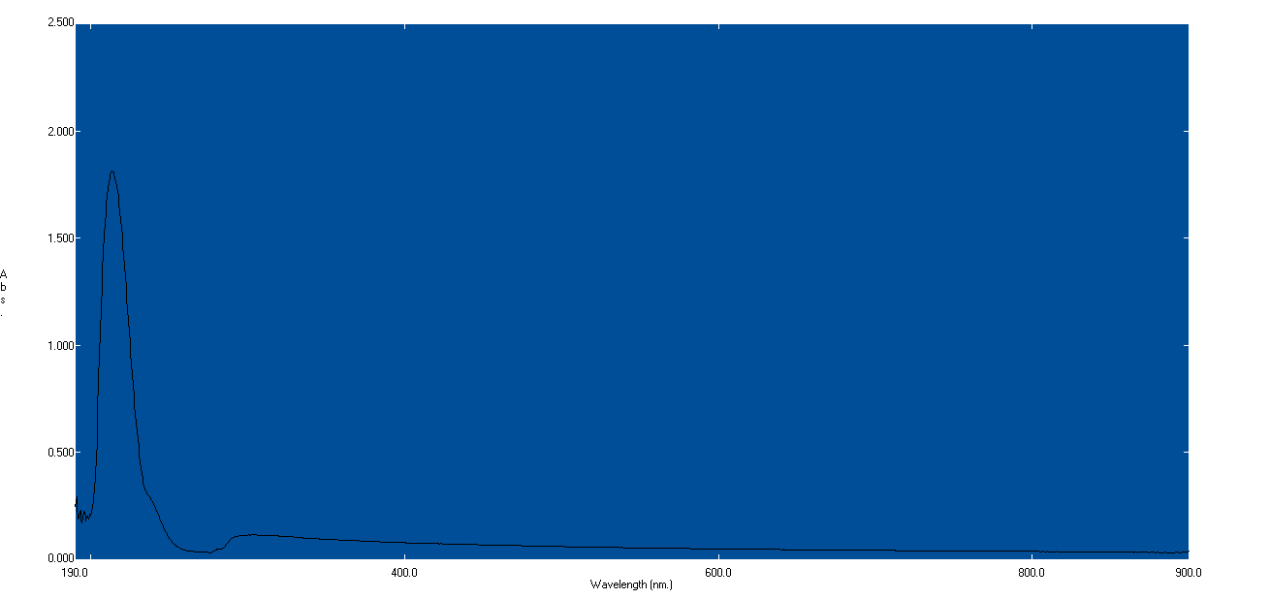


Figure S8. The UV spectrum of **1**.


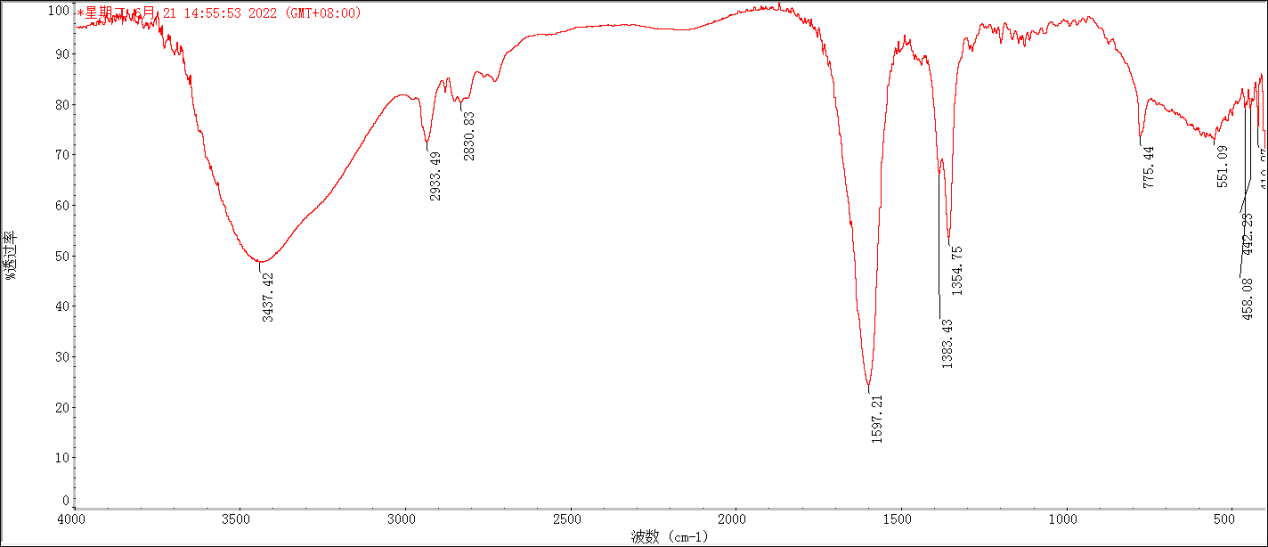


Figure S9. The IR spectrum of **1**.


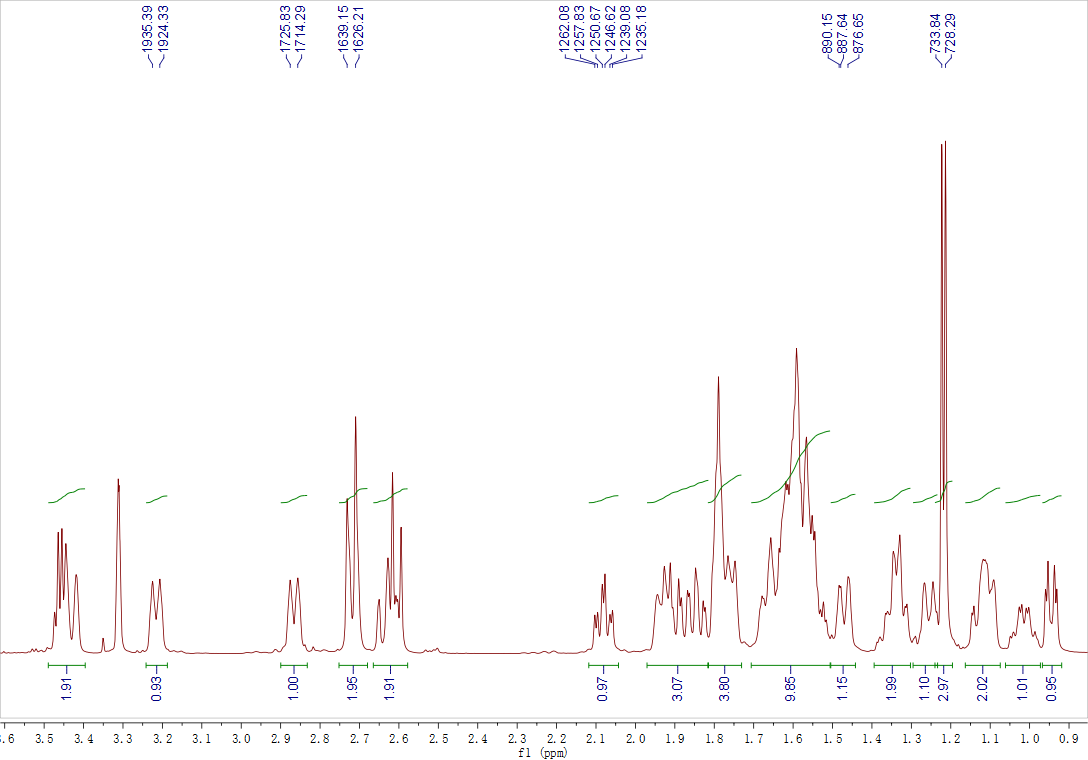


Figure S10. The ^1^H NMR spectrum of **2** in methanol-*d*_4_.


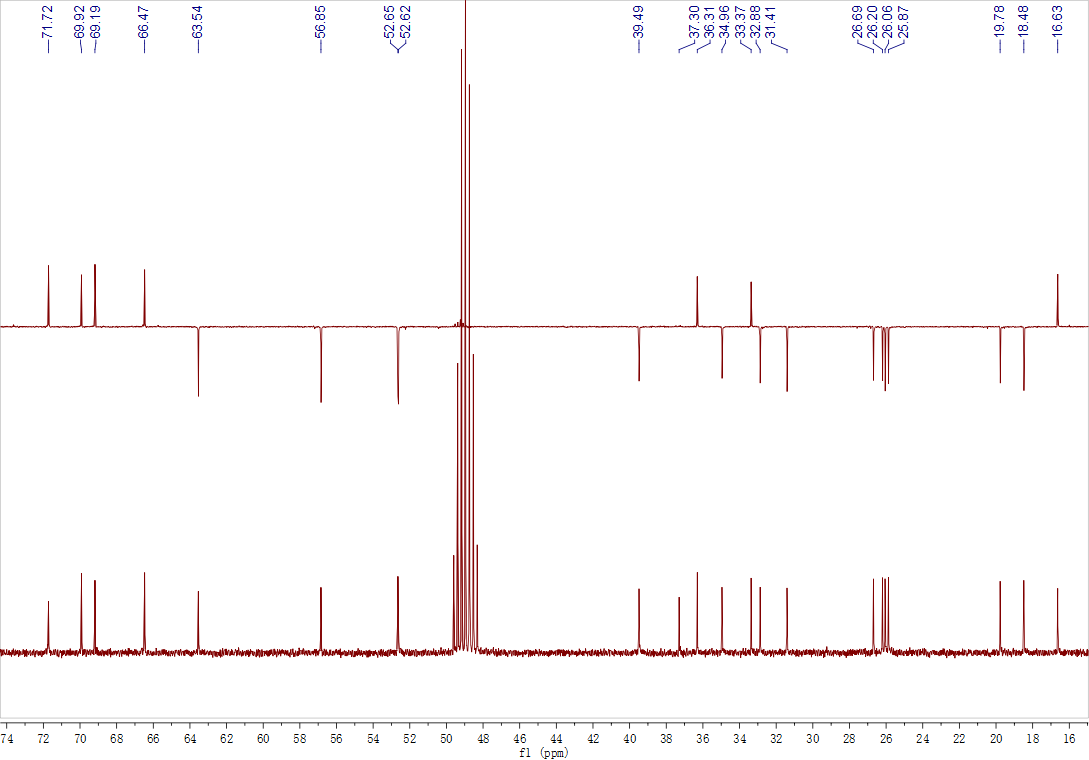


Figure S11. The ^13^C NMR spectrum of **2** in methanol-*d*_4_.


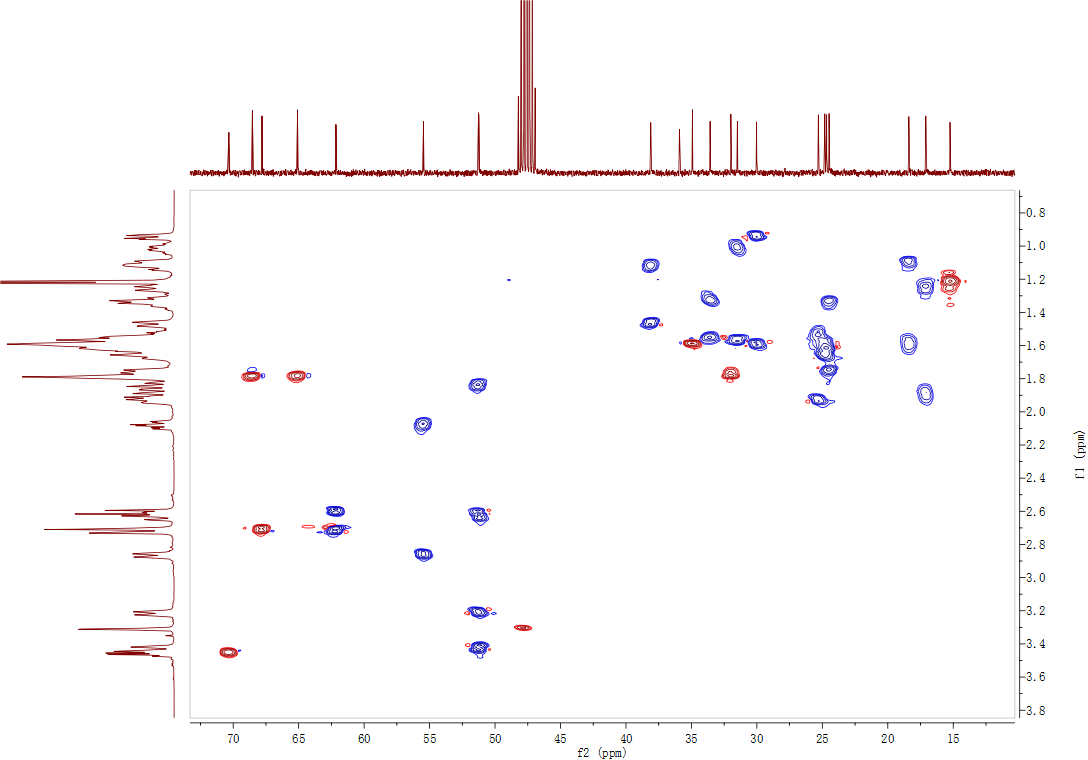


Figure S12. The HSQC spectrum of **2** in methanol-*d*_4_.


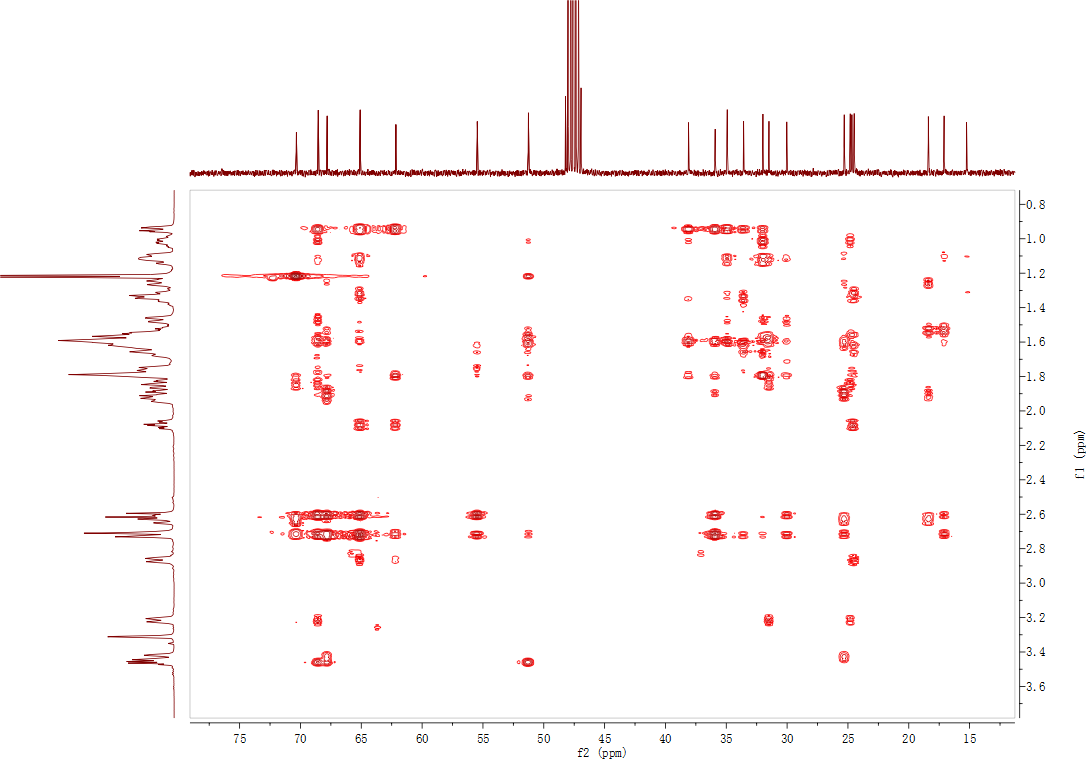


Figure S13. The HMBC spectrum of **2** in methanol-*d*_4_.


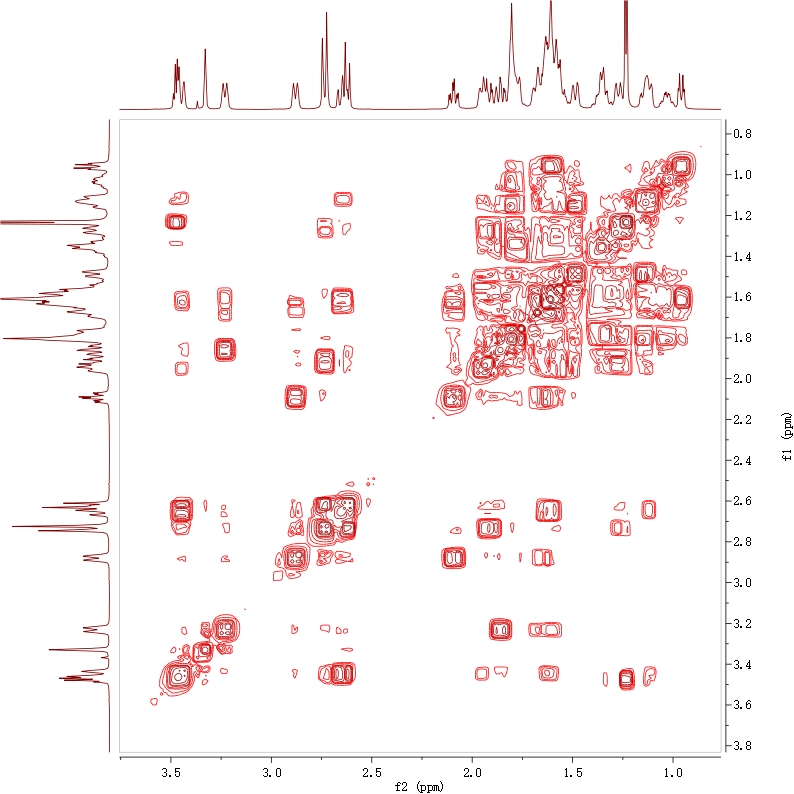


Figure S14. ^1^H-^1^H COSY spectrum of **2** in methanol-*d*_4_


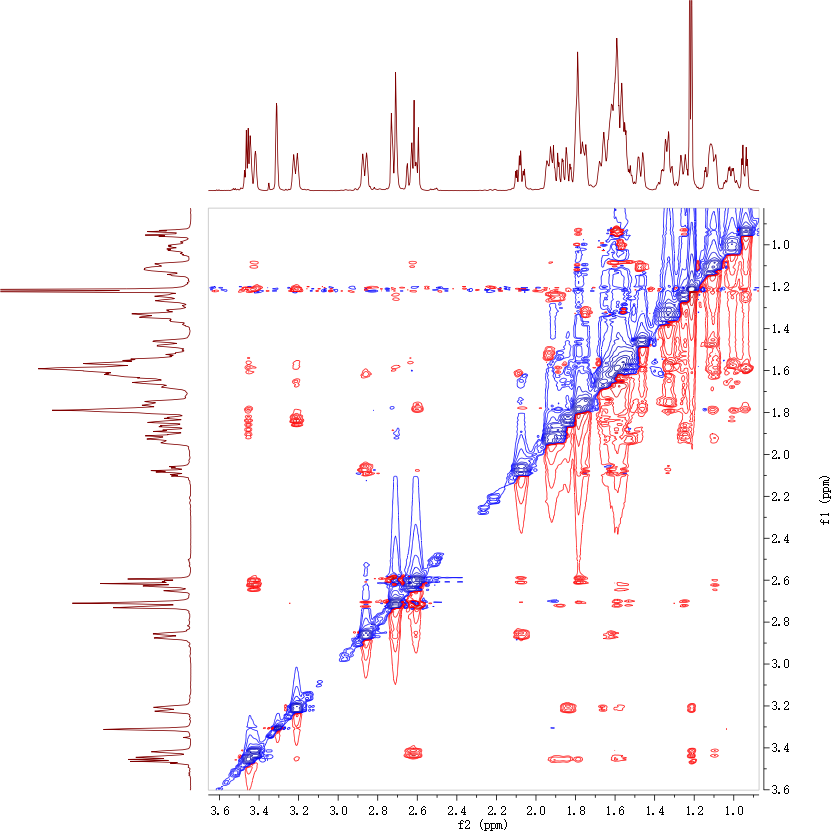


Figure S15. The ROESY spectrum of **2** in methanol-*d*_4_.


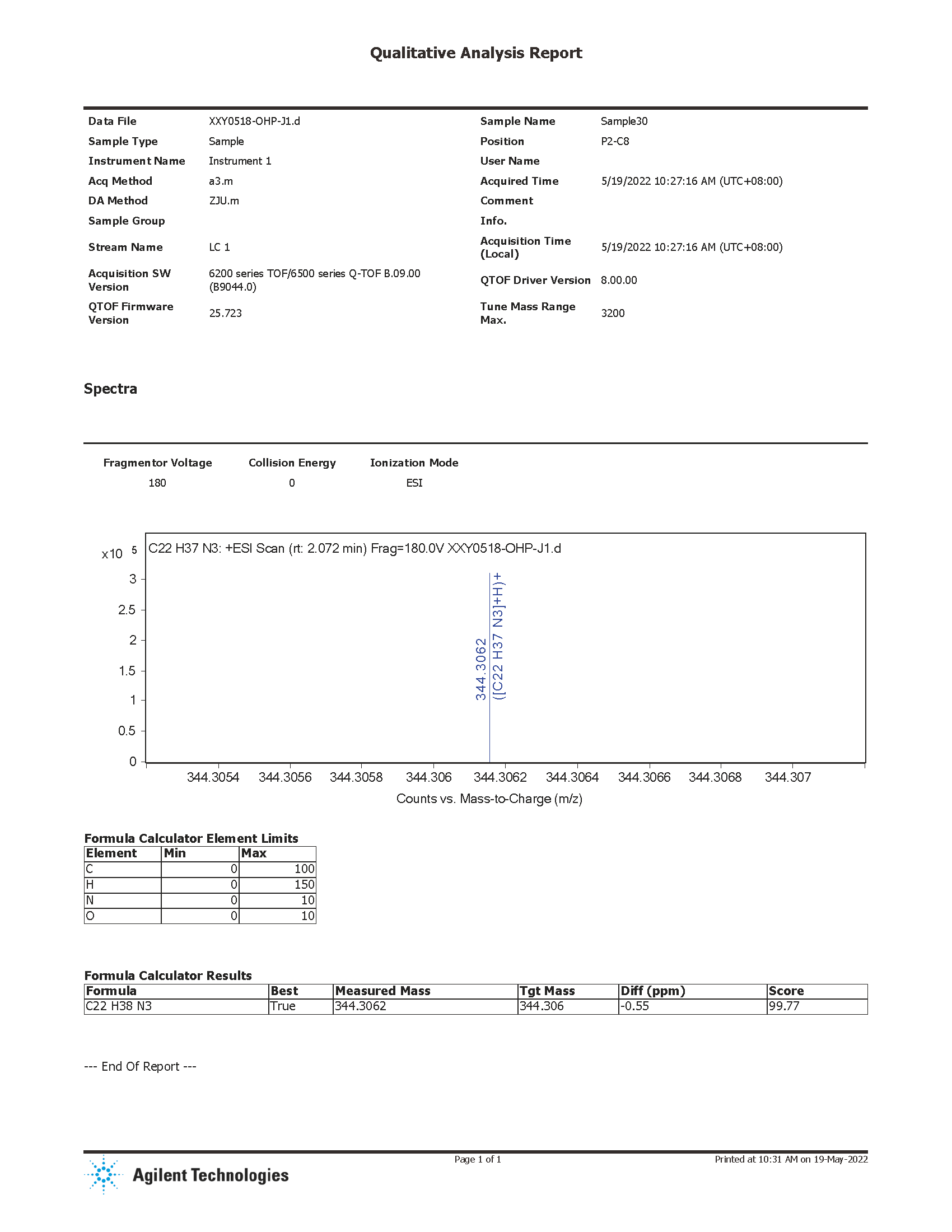


Figure S16. The HRESIMS data of **2**.


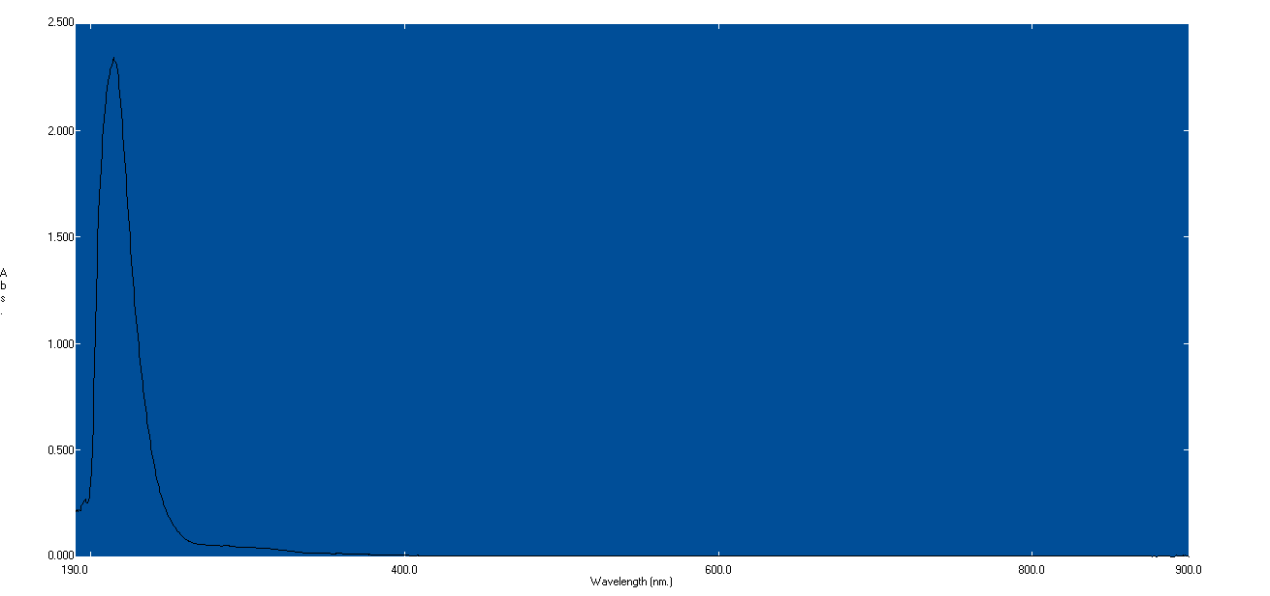


Figure S17. The UV spectrum of **2**.


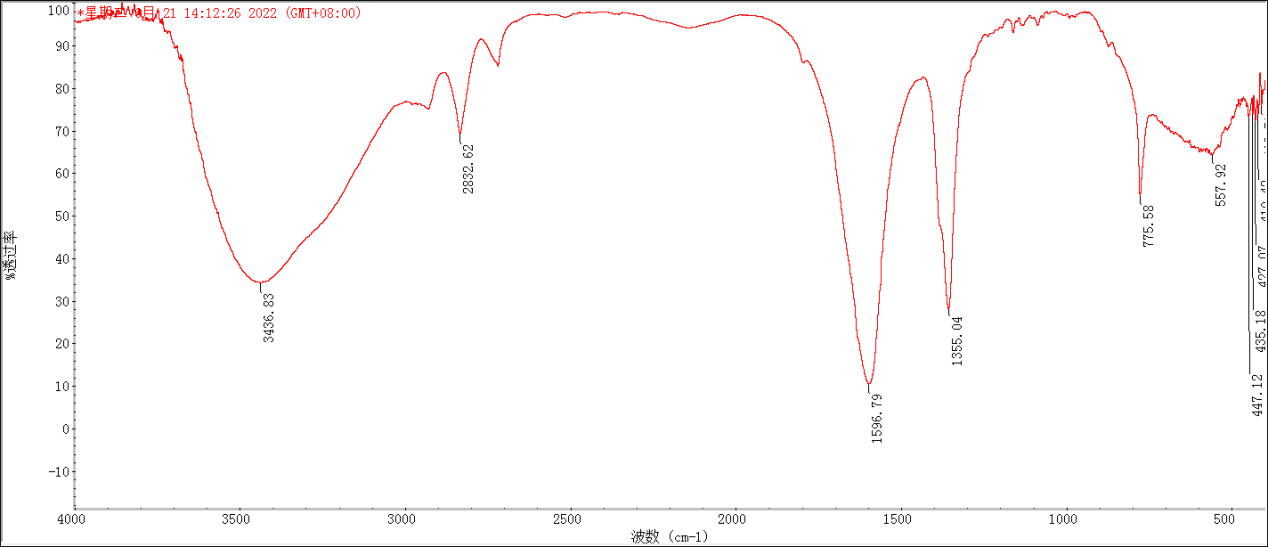


Figure S18. The IR spectrum of **2**.


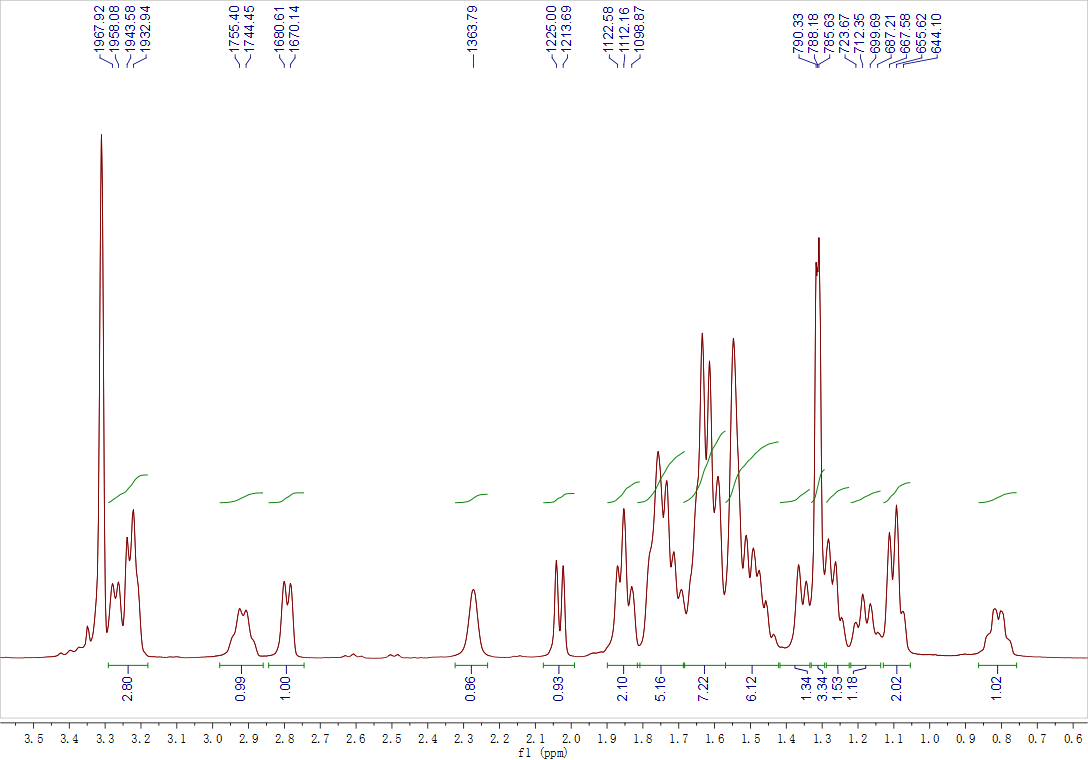


Figure S19. The ^1^H NMR spectrum of **3** in methanol-*d*_4_.


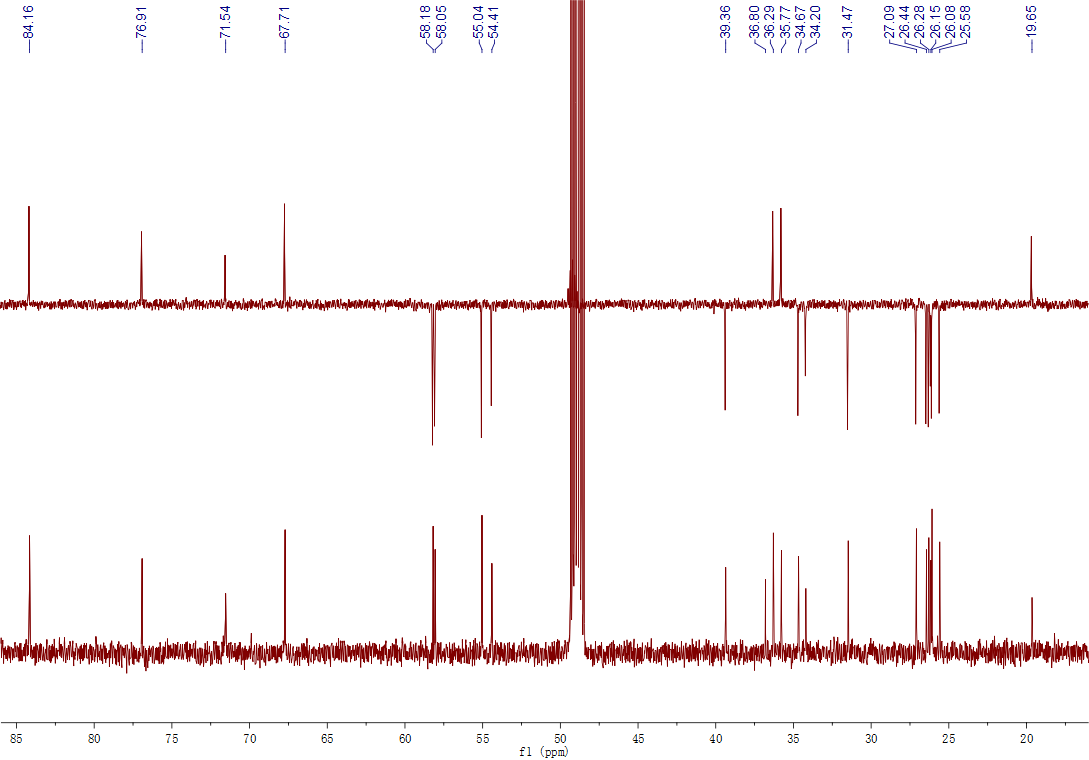


Figure S20. The ^13^C NMR spectrum of **3** in methanol-*d*_4_.


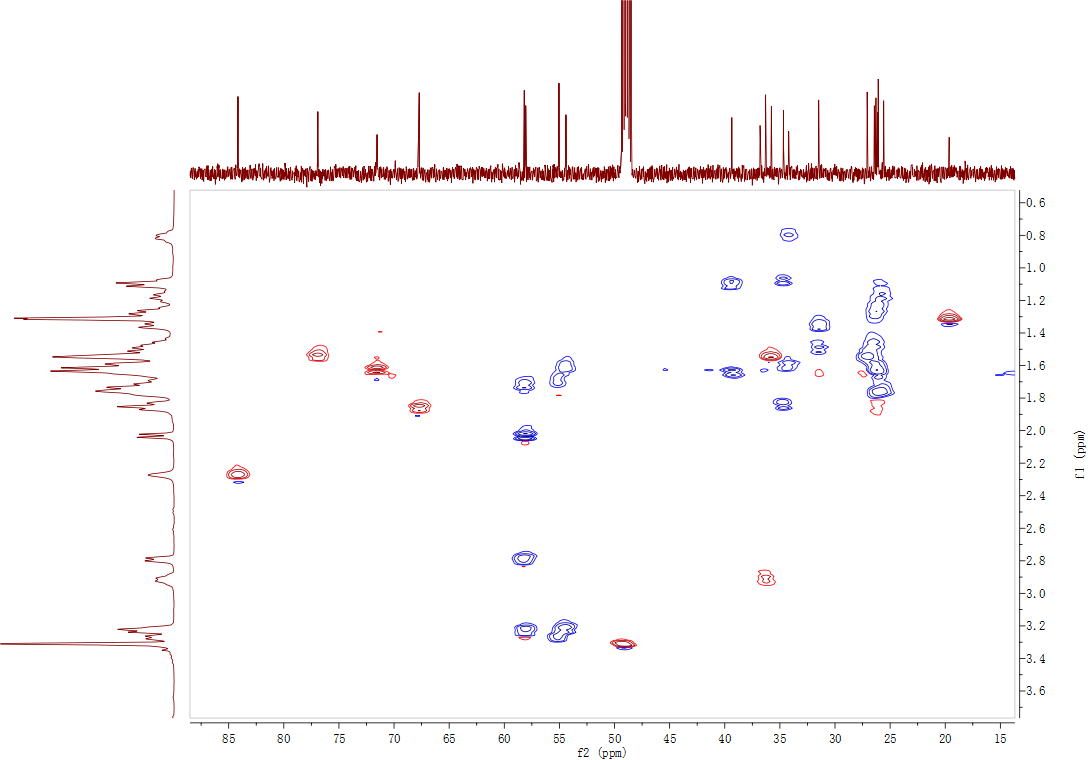


Figure S21. The HSQC spectrum of **3** in methanol-*d*_4_.


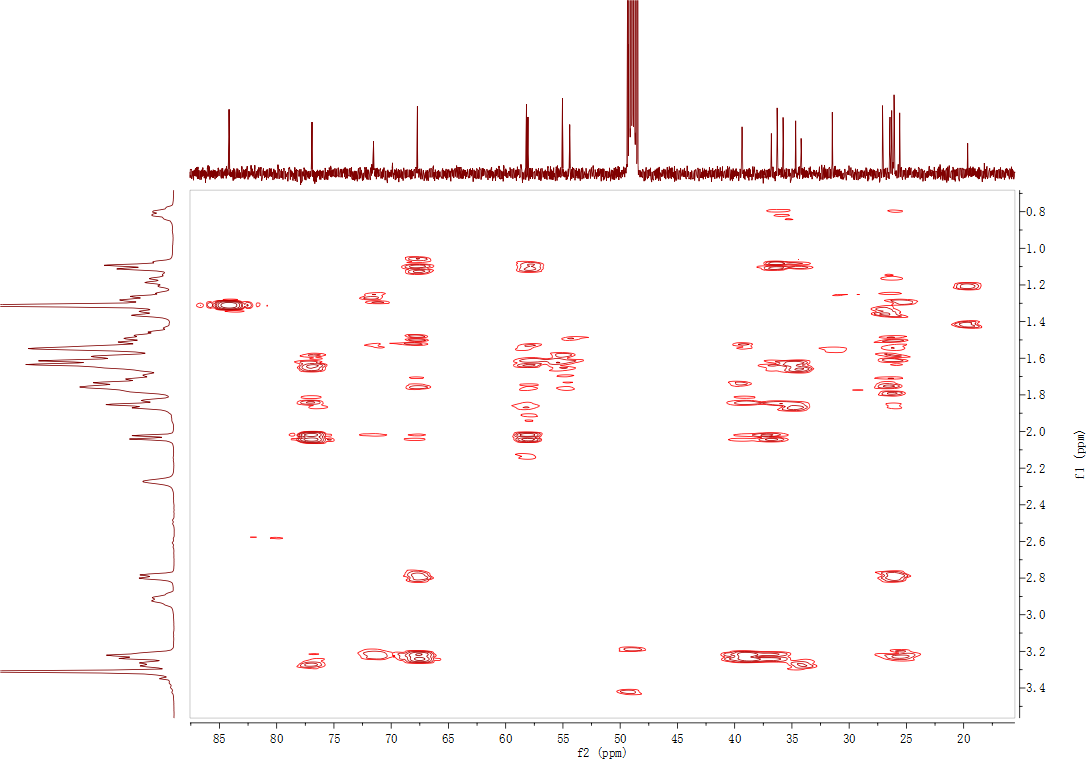


Figure S22. The HMBC spectrum of **3** in methanol-*d*_4_.


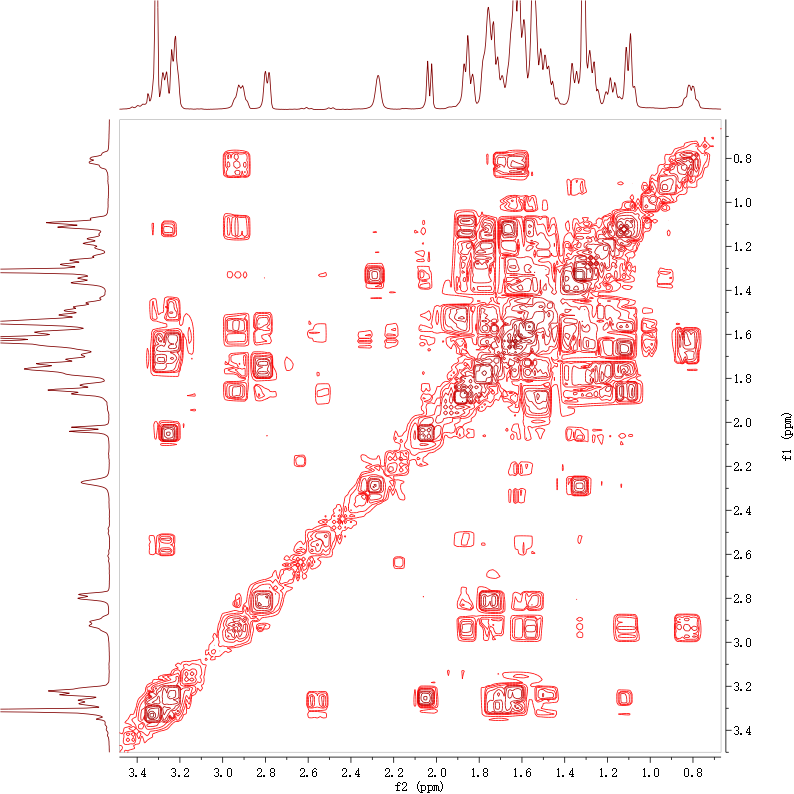


Figure S23. ^1^H-^1^H COSY spectrum of **3** in methanol-*d*_4_


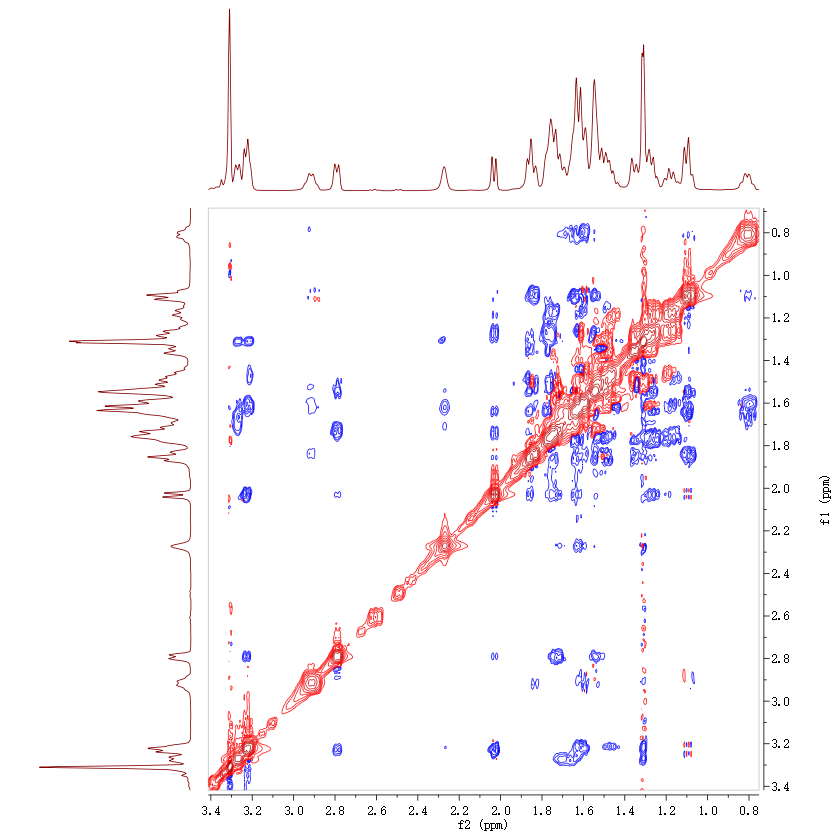


Figure S24. The ROESY spectrum of **3** in methanol-*d*_4_.


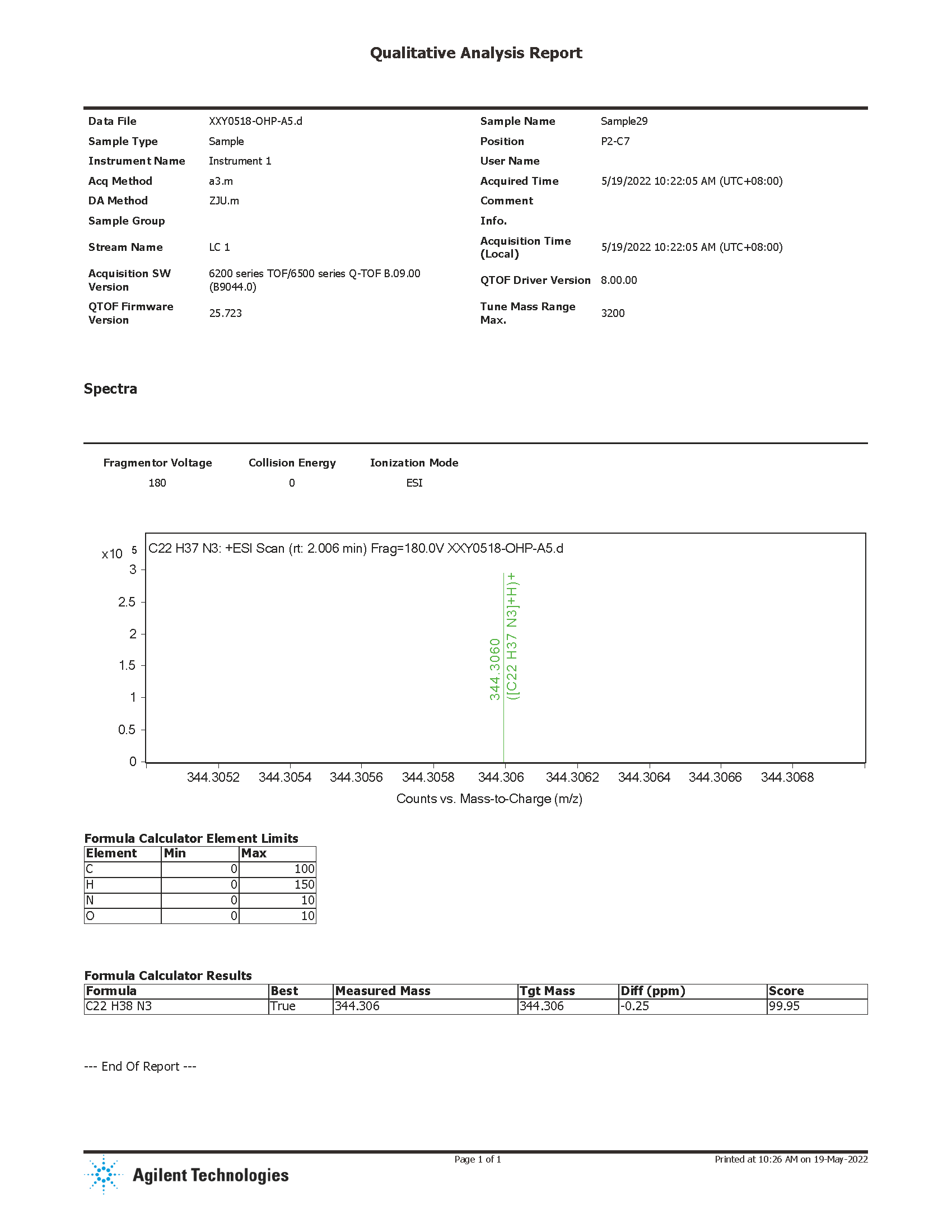


Figure S25. The HRESIMS data of **3**.


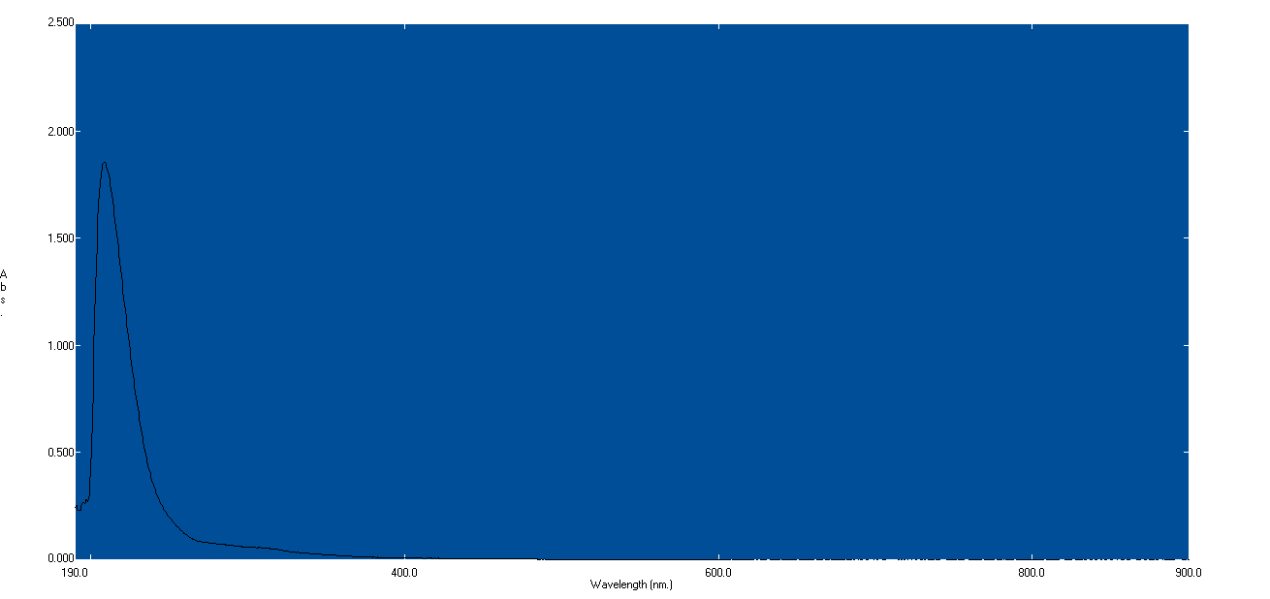


Figure S26. The UV spectrum of **3**.


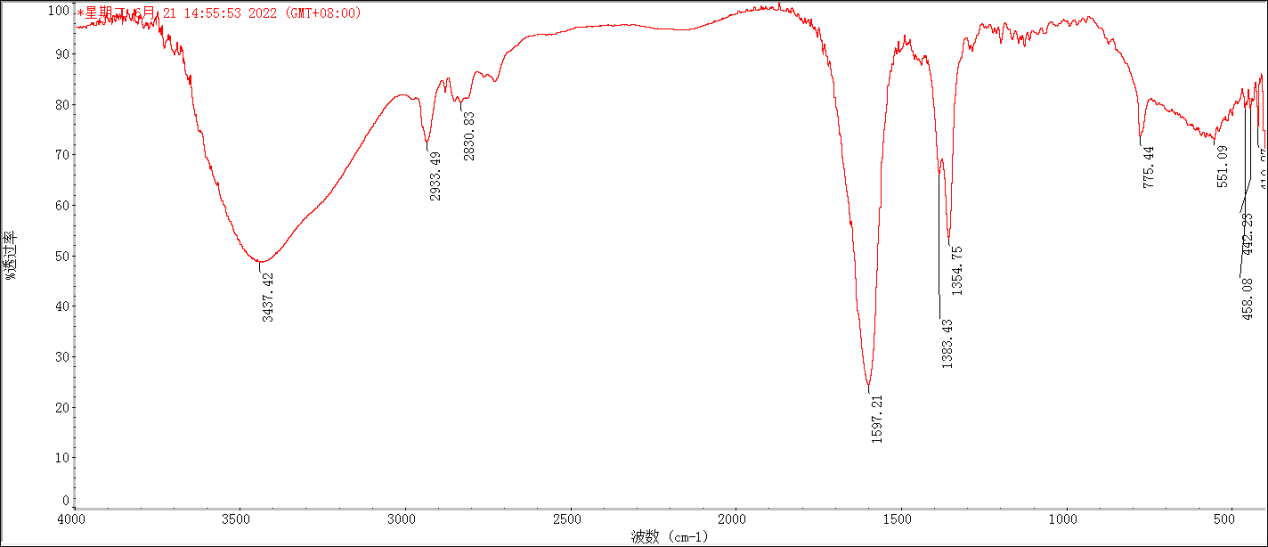


Figure S27. The IR spectrum of **3**.


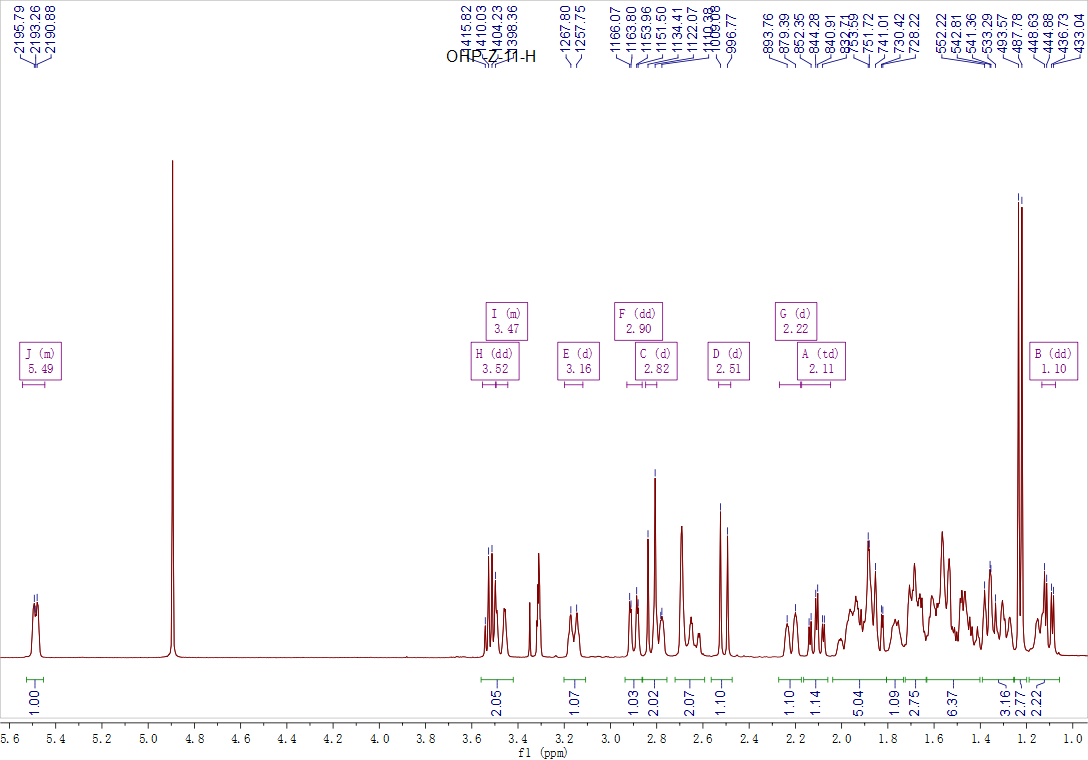


Figure S28. The ^1^H NMR spectrum of **4** in methanol-*d*_4_.


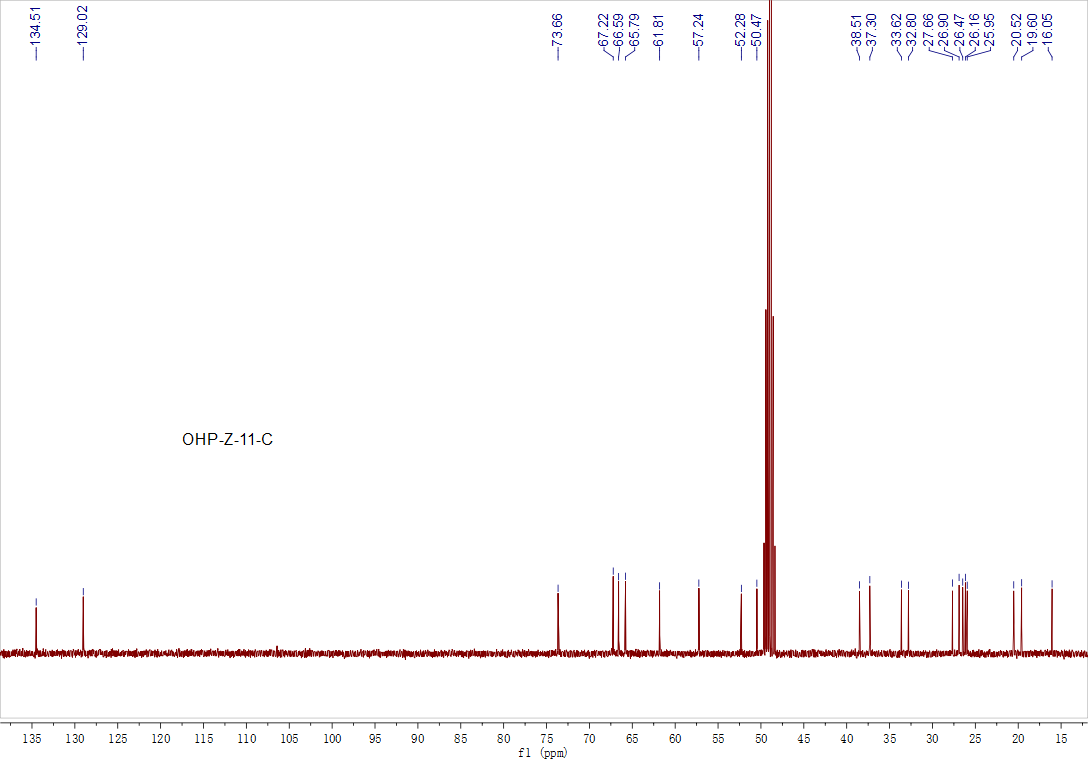


Figure S29. The ^13^C NMR spectrum of **4** in methanol-*d*_4_


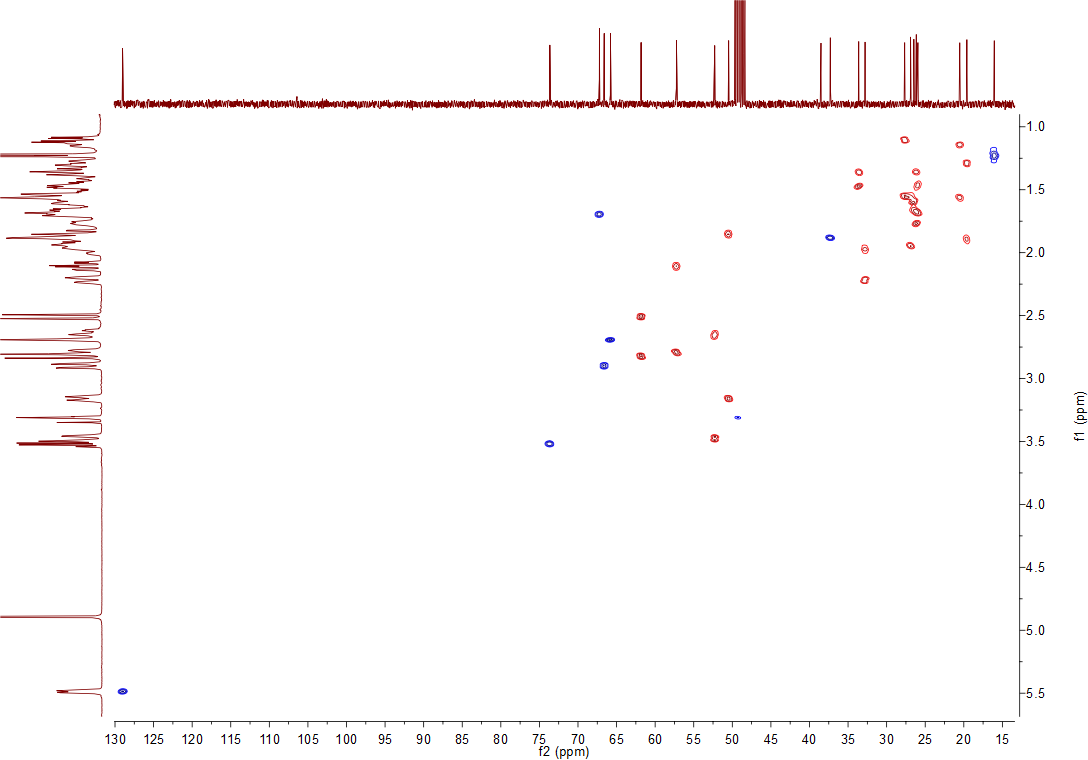


Figure S30. The HSQC spectrum of **4** in methanol-*d*_4_


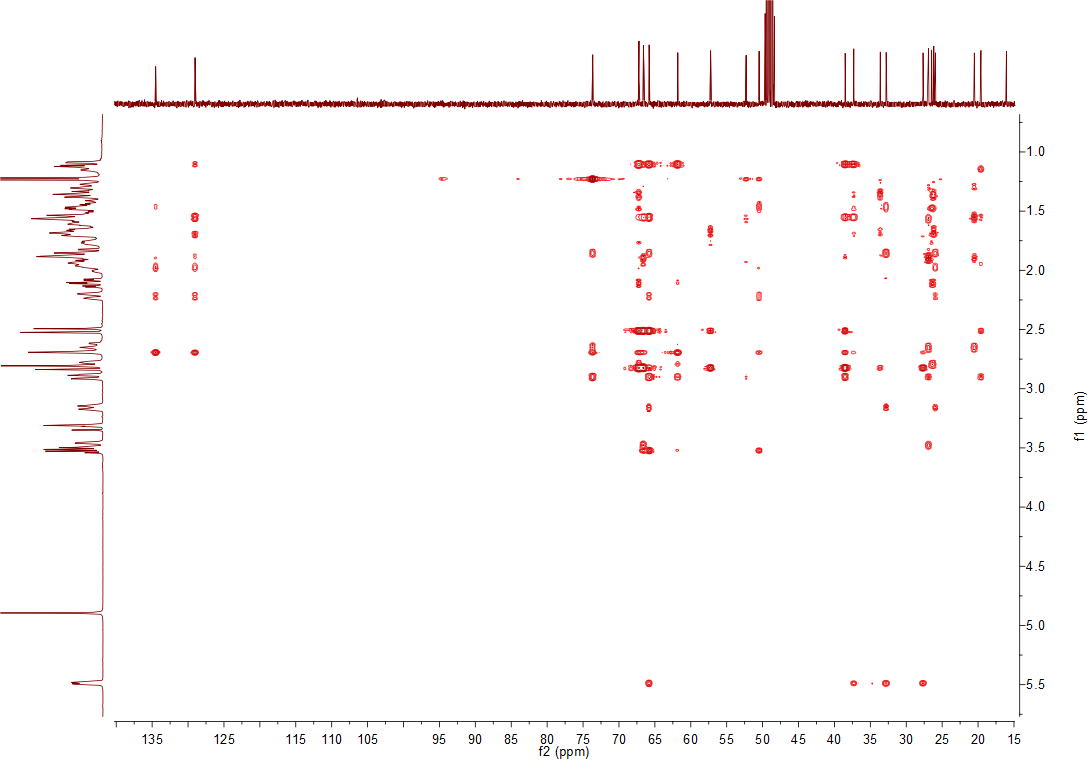


Figure S31. The HMBC spectrum of **4** in methanol-*d*_4_


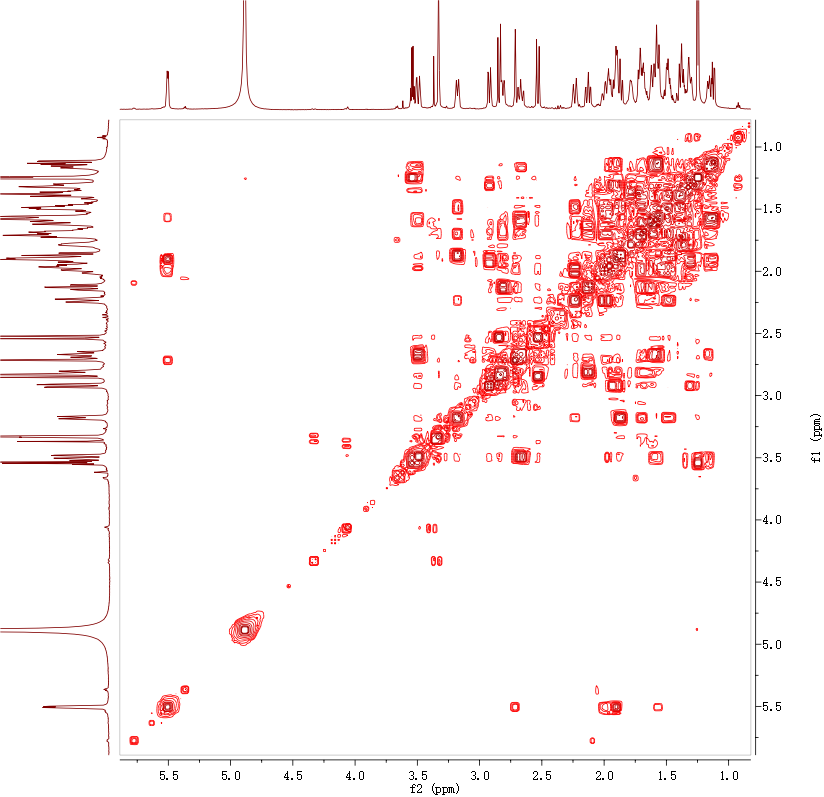


Figure S32. ^1^H-^1^H COSY spectrum of **4** in methanol-*d*_4_


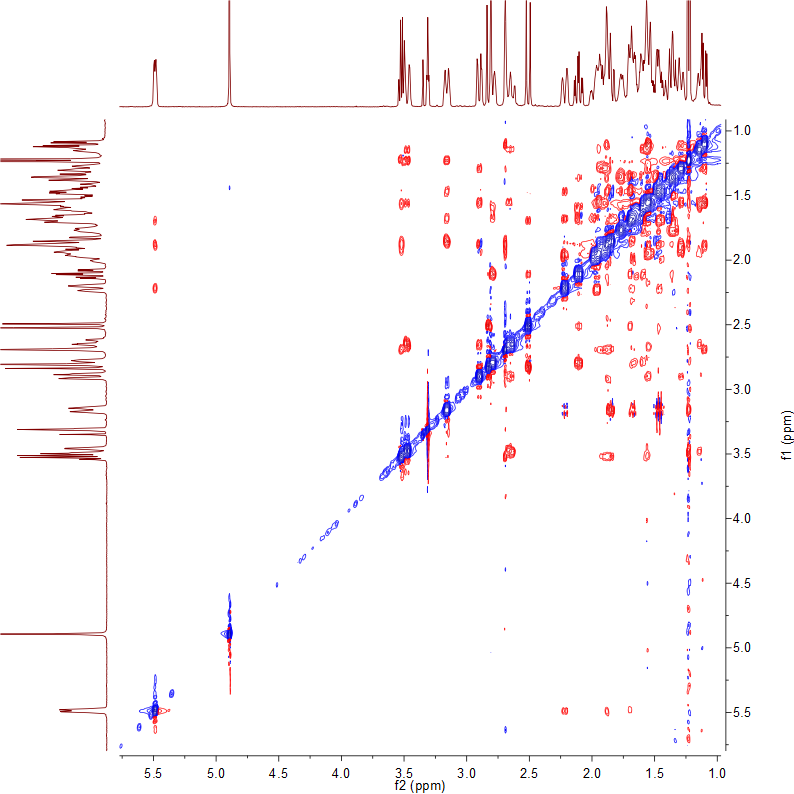


Figure S33. The ROESY spectrum of **4** in methanol-*d*_4_





Figure S34. HRESIMS spectrum of **4**


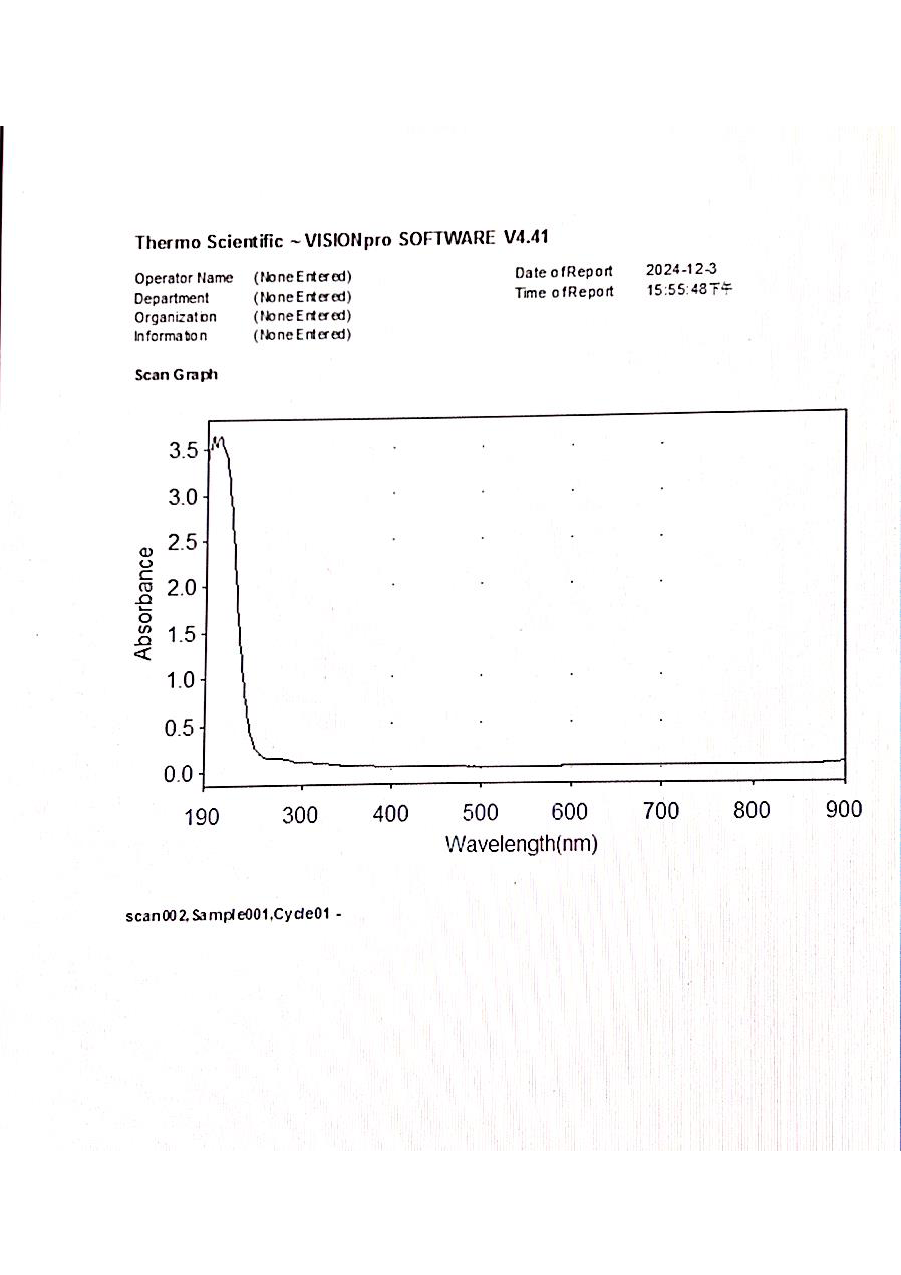


Figure S35. UV spectrum of **4**


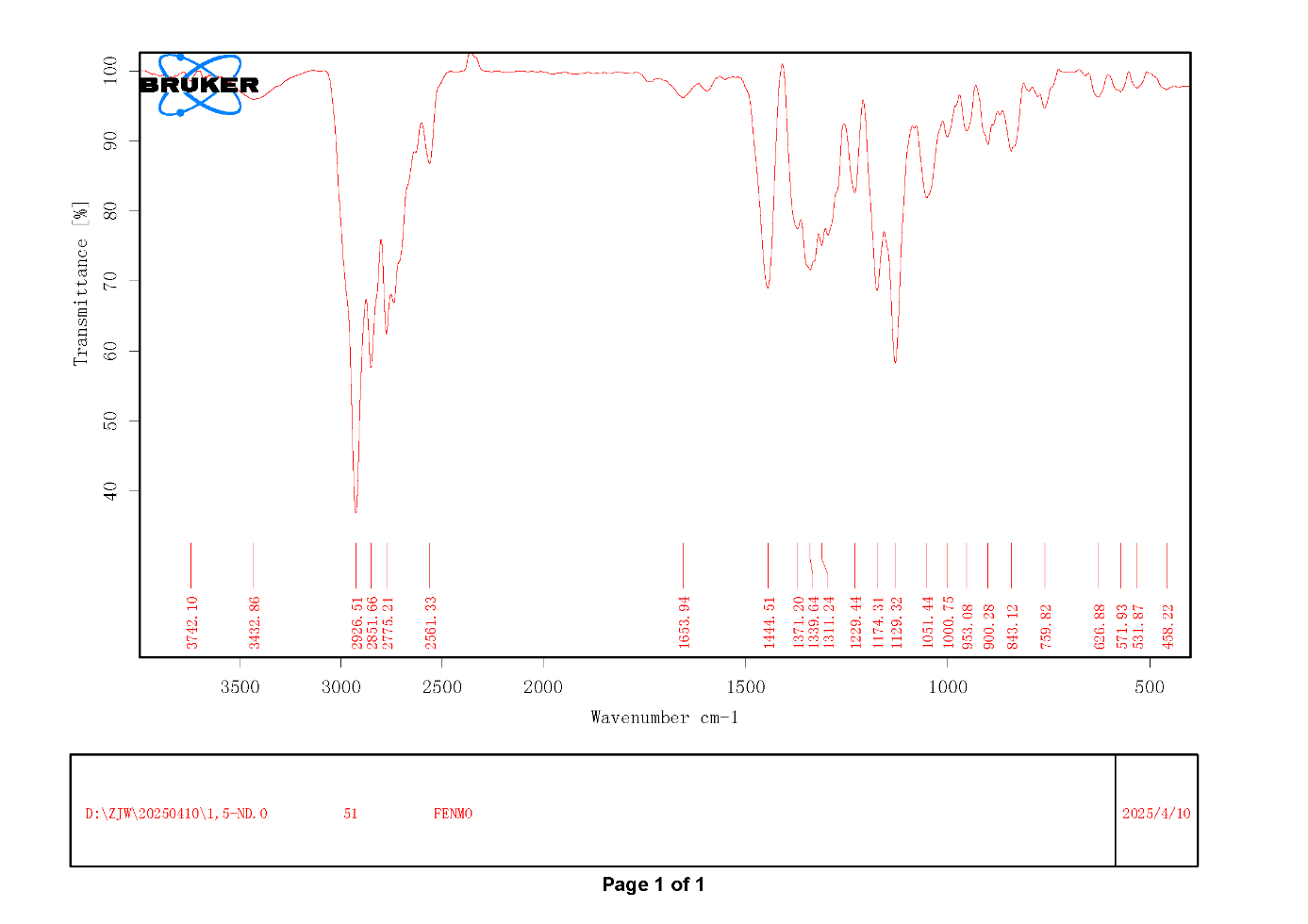


Figure S36. IR spectrum of **4**


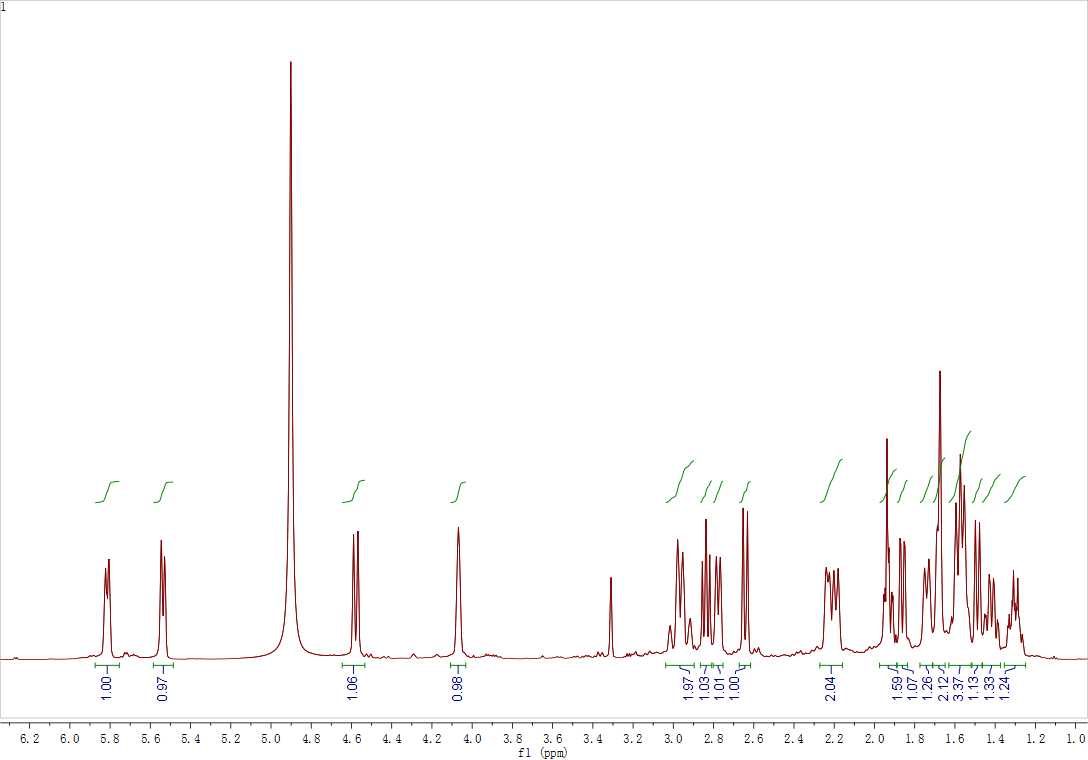


Figure S37. The ^1^H NMR spectrum of **7** in methanol-*d*_4_.


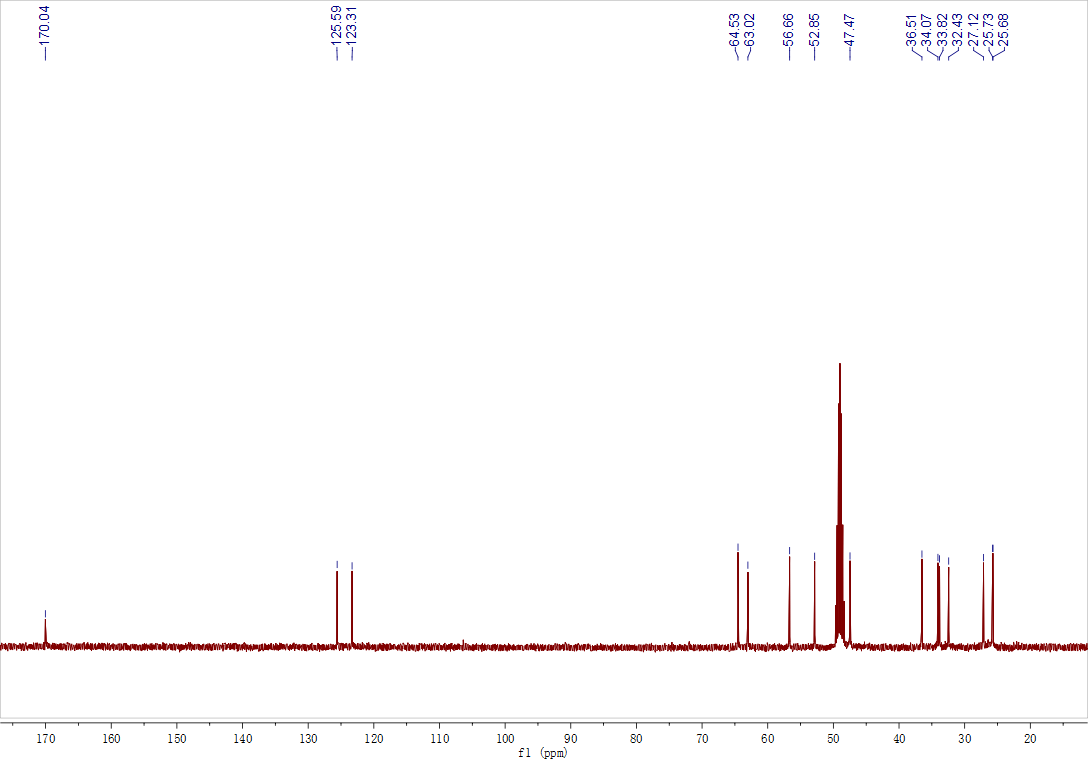


Figure S38. The ^13^C NMR spectrum of **7** in methanol-*d*_4_.


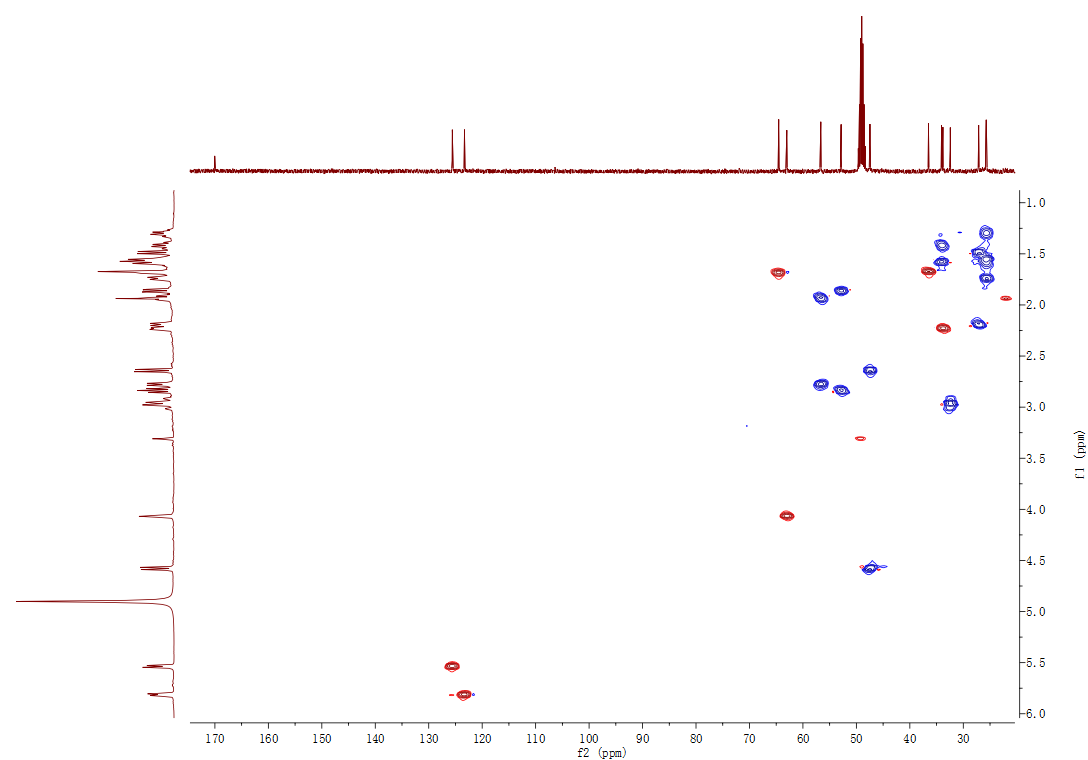


Figure S39. The HSQC spectrum of **7** in methanol-*d*_4_.


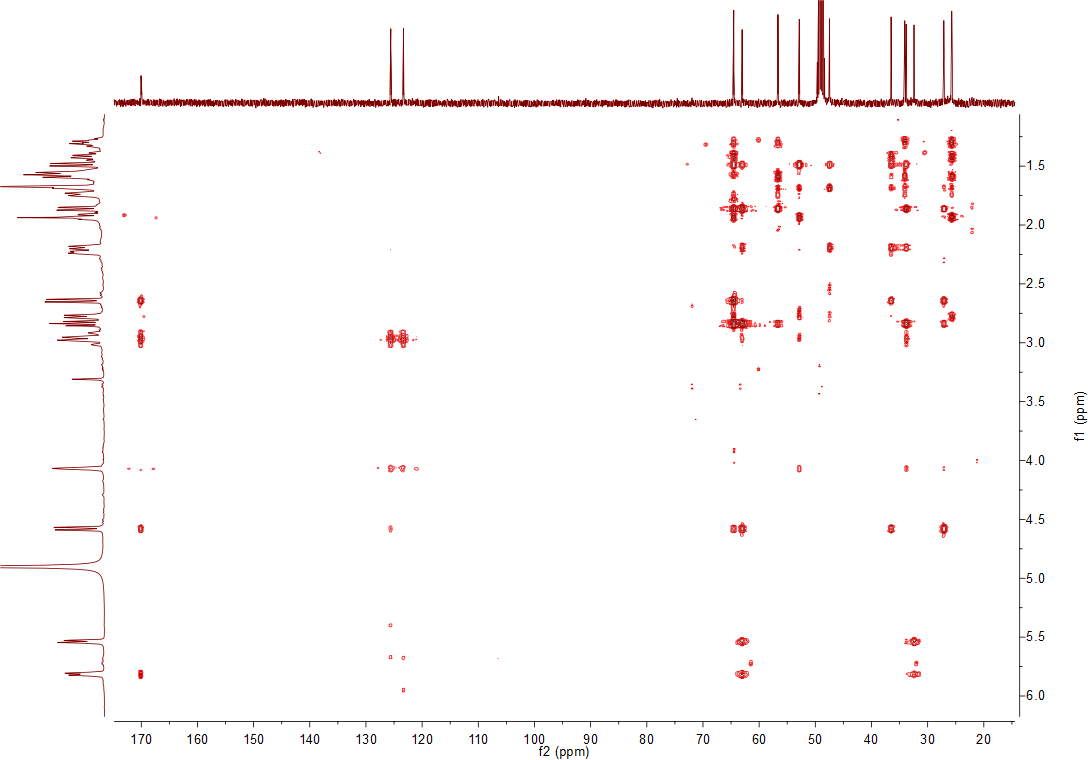


Figure S40. The HMBC spectrum of **7** in methanol-*d*_4_.


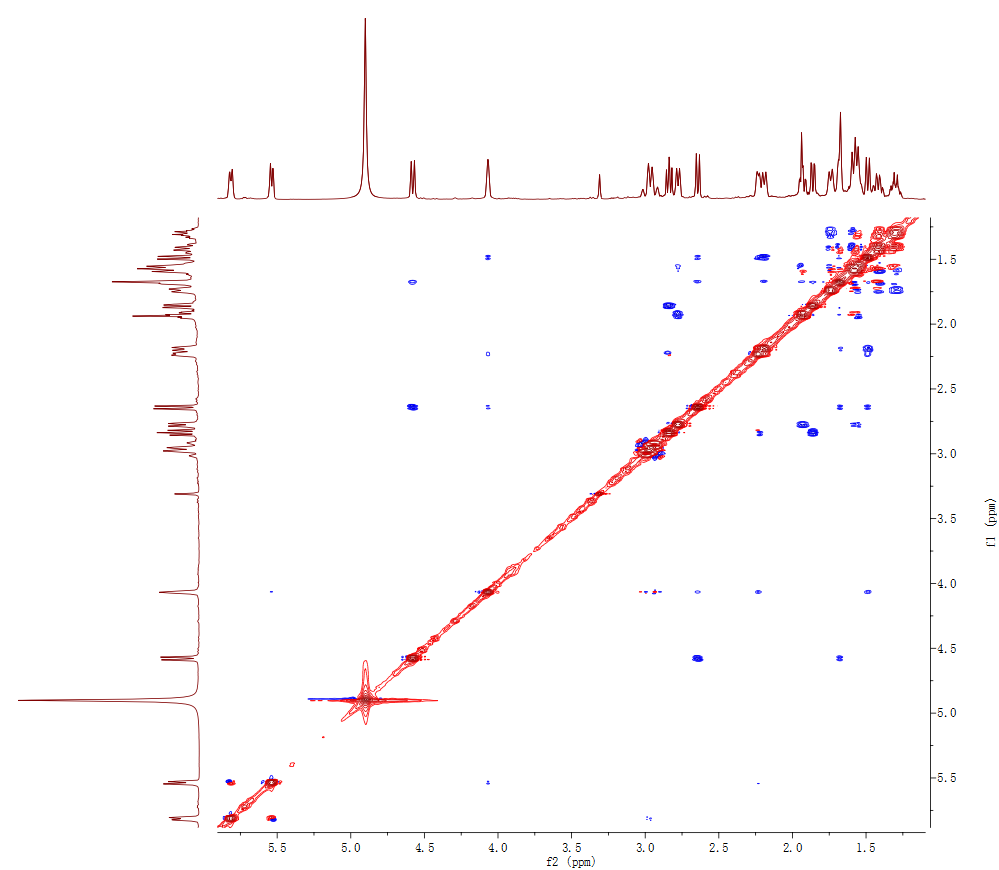


Figure S41. The ^1^H-^1^H COSY spectrum of **7** in methanol-*d*_4_.


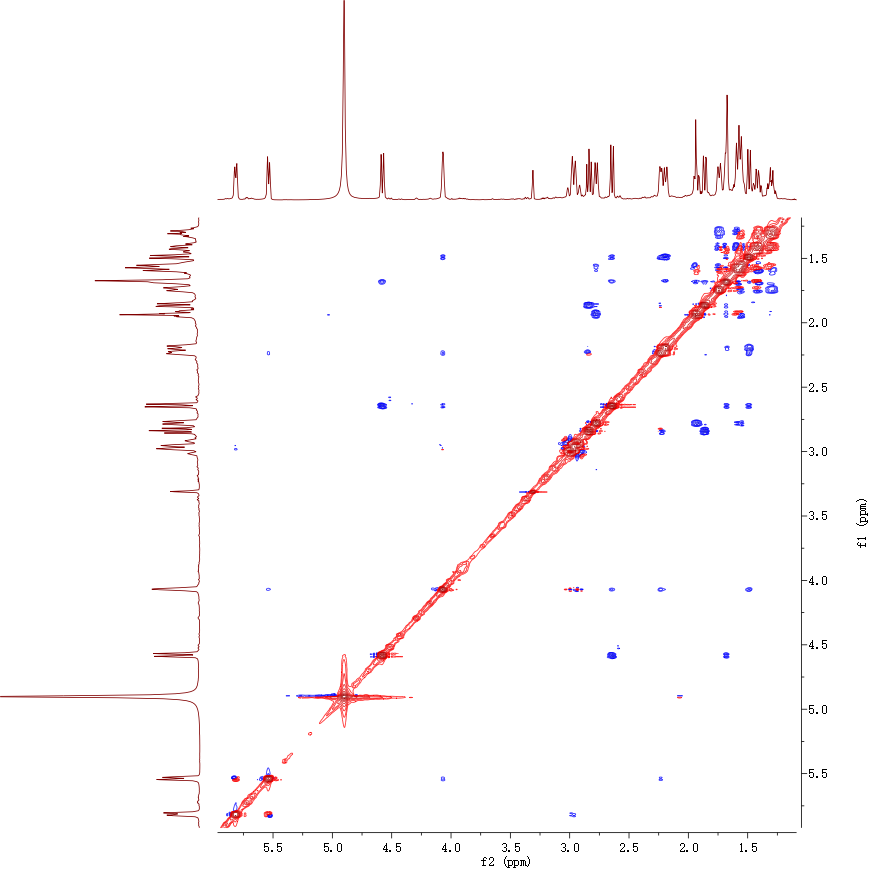


Figure S42. The ROESY spectrum of **7** in methanol-*d*_4_.





Figure S43. The HRESIMS spectrum of of **7**.


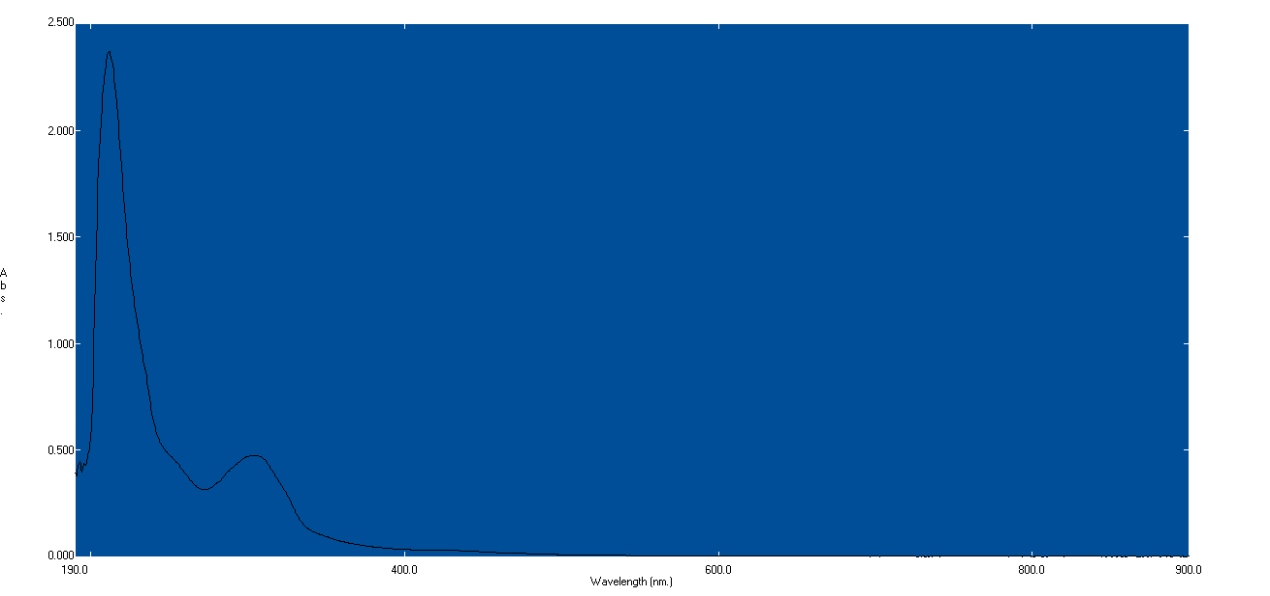


Figure S44. The UV spectrum of **7**.


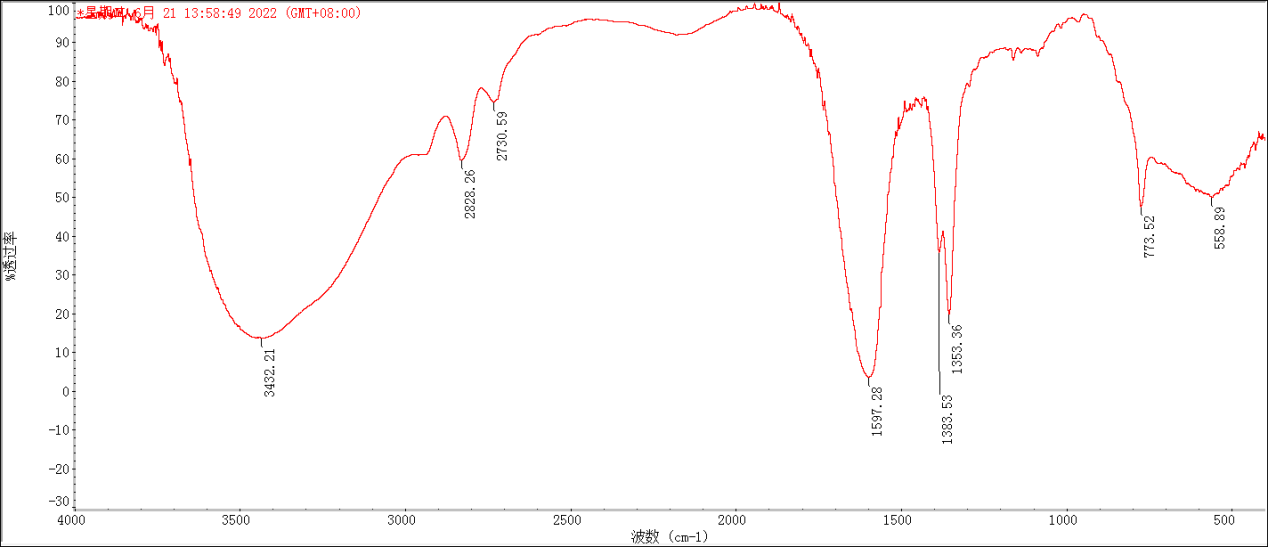


Figure S45. The IR spectrum of **7**.


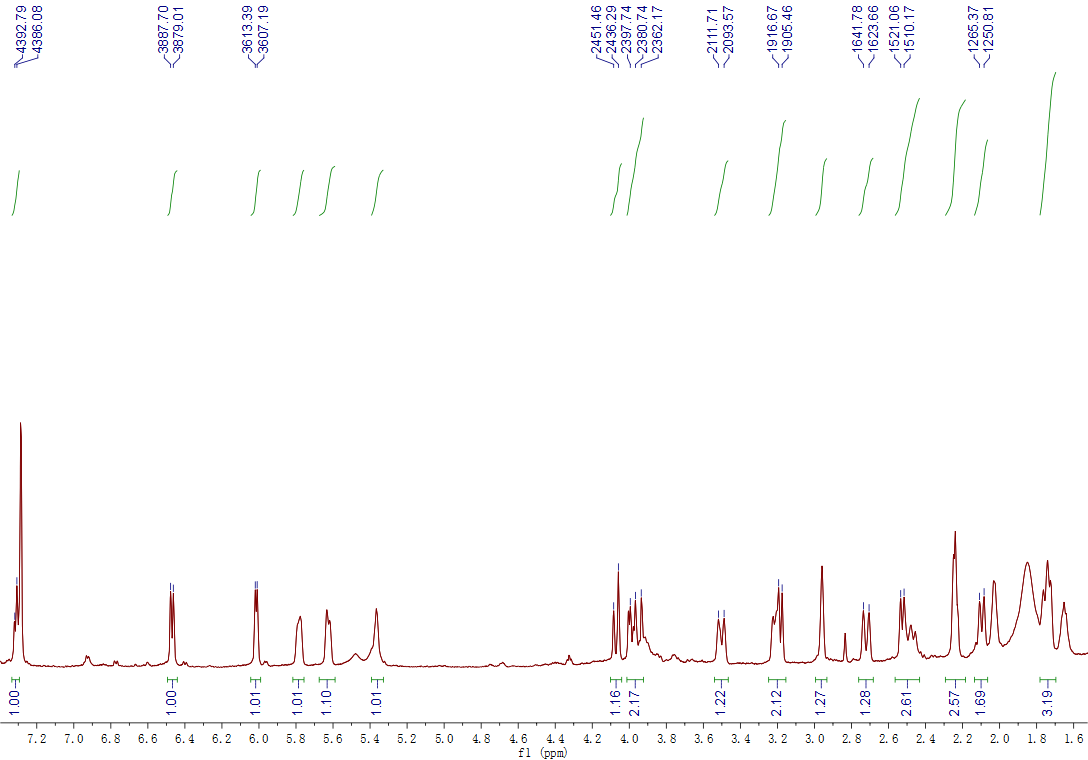


Figure S46. The ^1^H NMR spectrum of **8** in CDCl_3_.


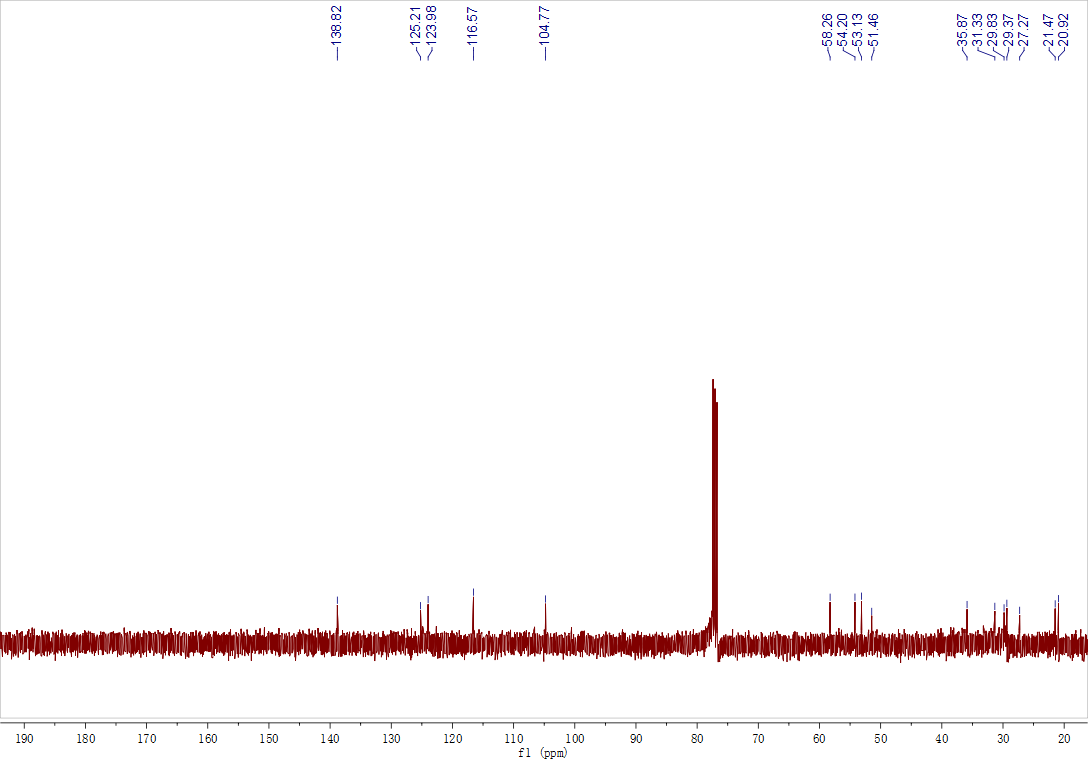


Figure S47. The ^13^C NMR spectrum of **8** in CDCl_3_.


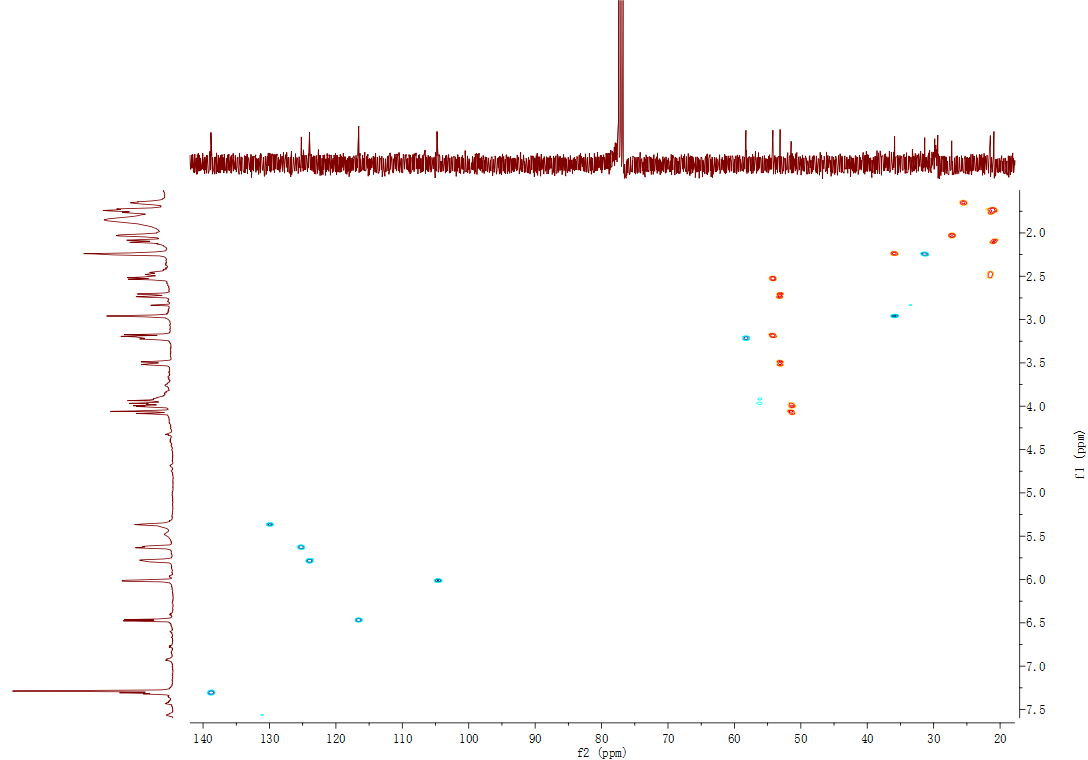


Figure S48. The HSQC spectrum of **8** in CDCl_3_.


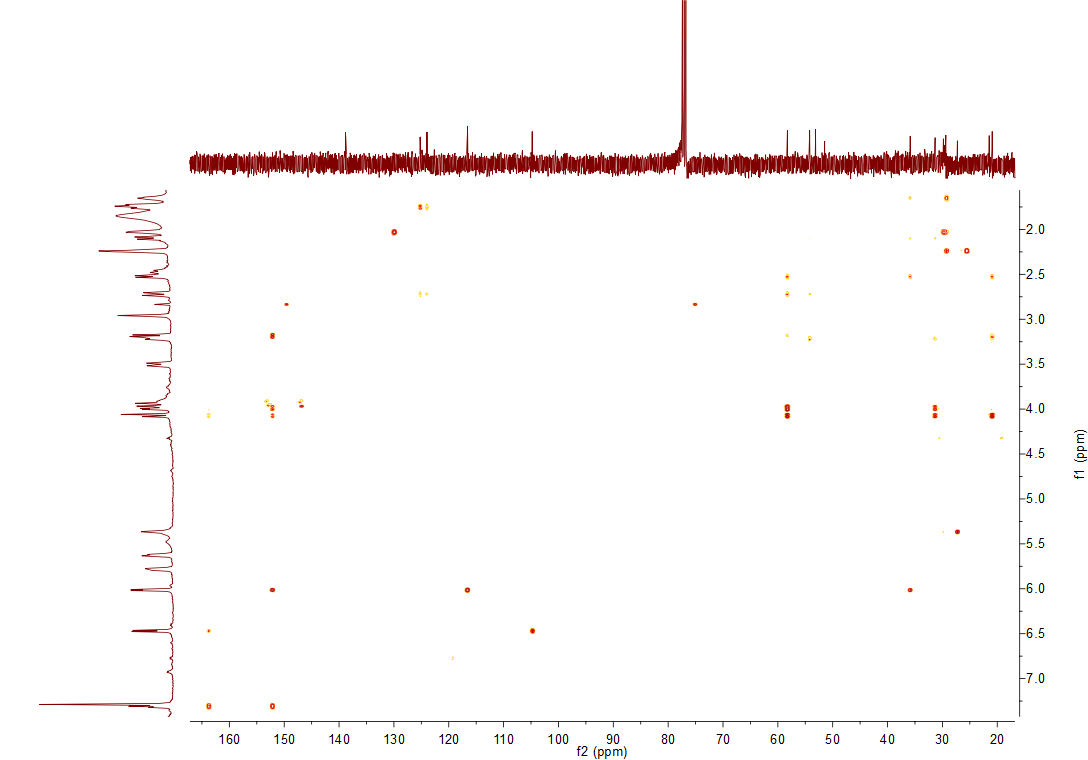


Figure S49. The HMBC spectrum of **8** in CDCl_3_.


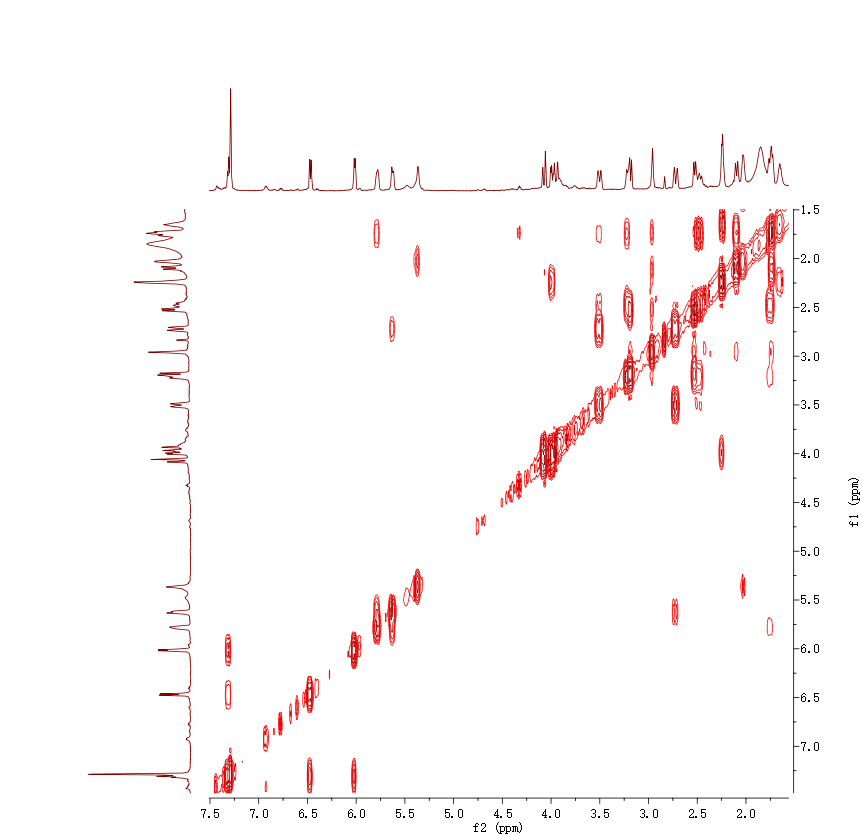


Figure S50. The ^1^H-^1^H COSY spectrum of **8** in CDCl_3_.


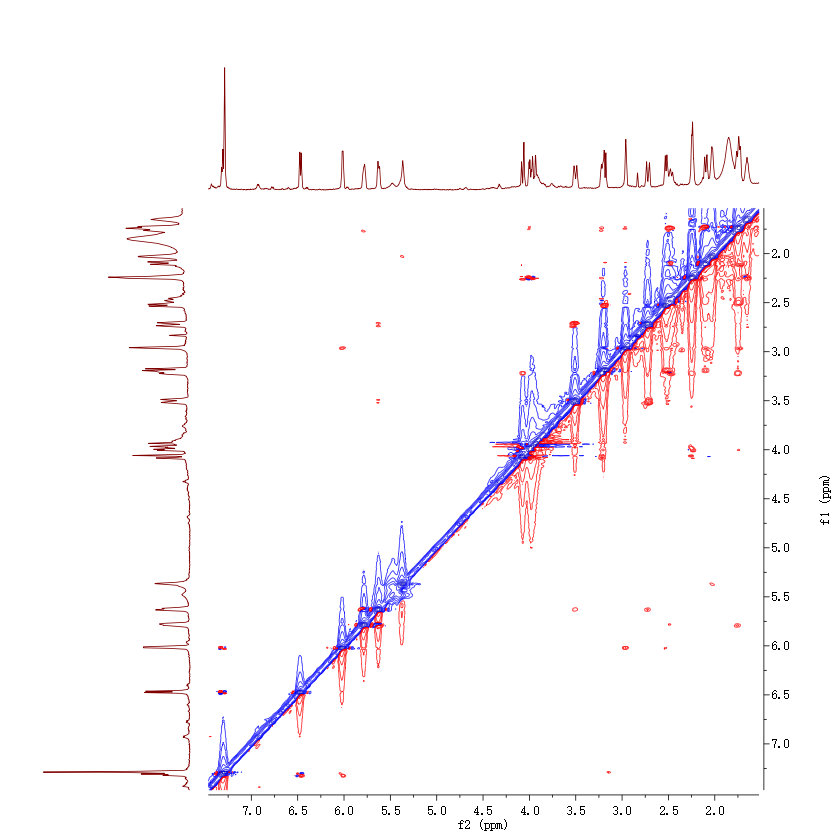


Figure S51. The ROESY spectrum of **8** in CDCl_3_.

**
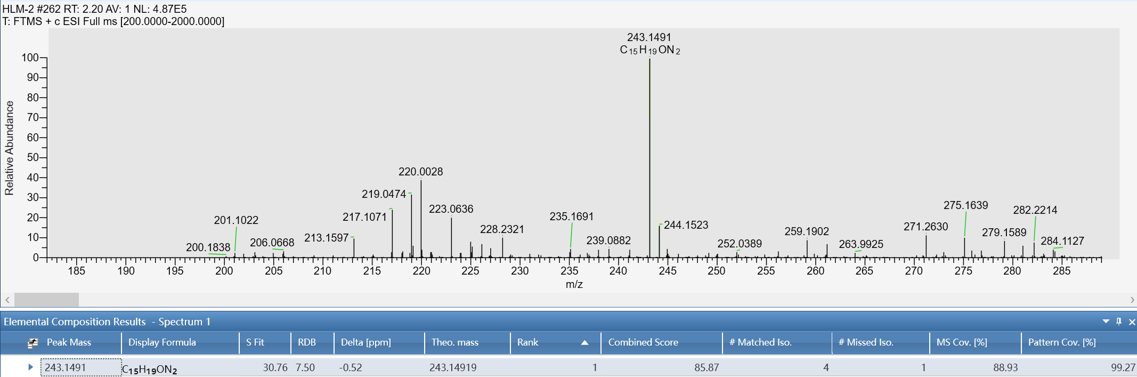
**

Figure S52. HRESIMS spectrum of **8**


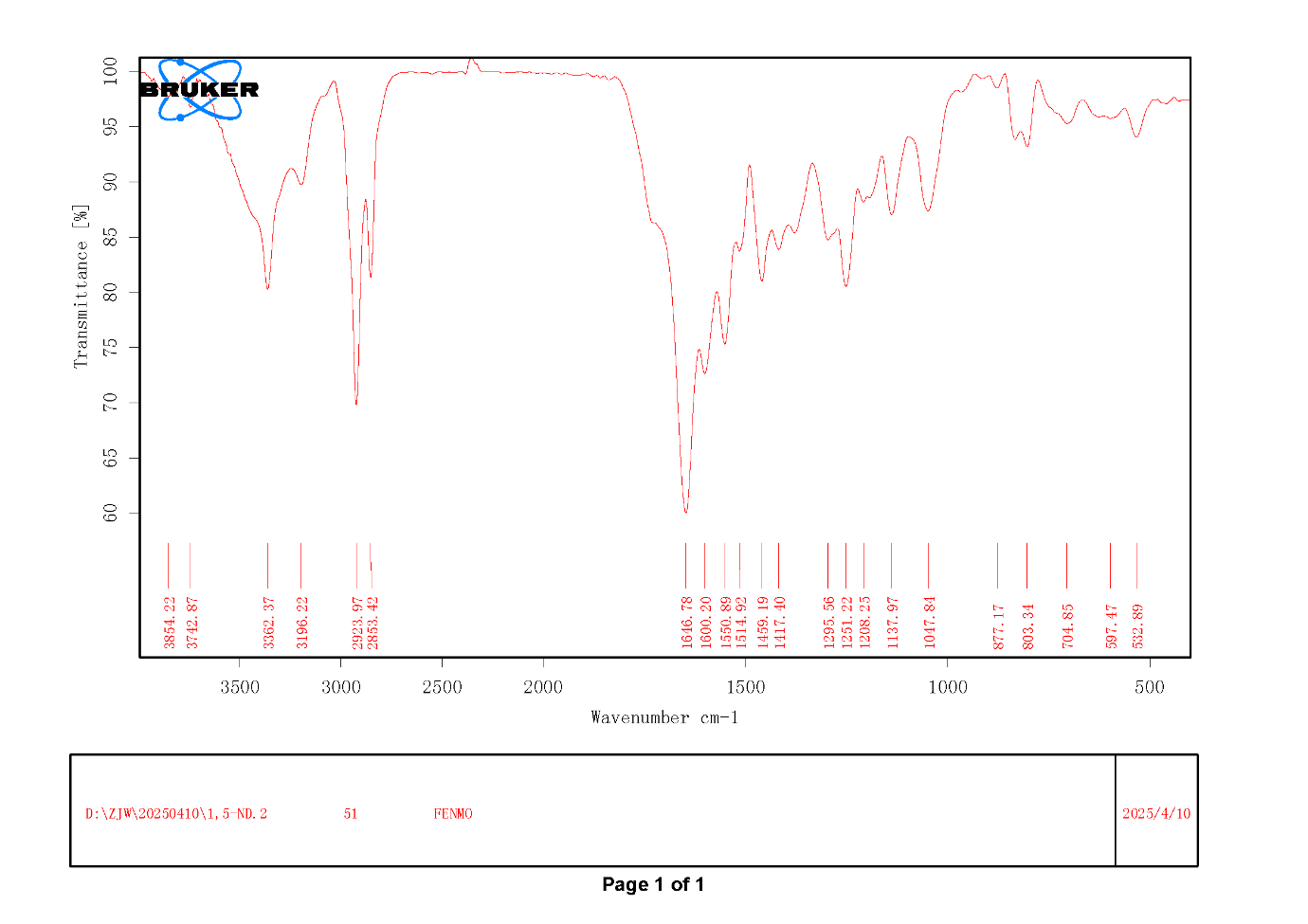


Figure S53. IR spectrum of **8**


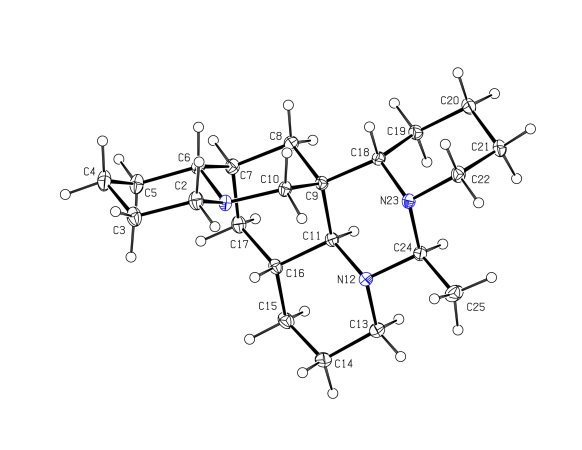


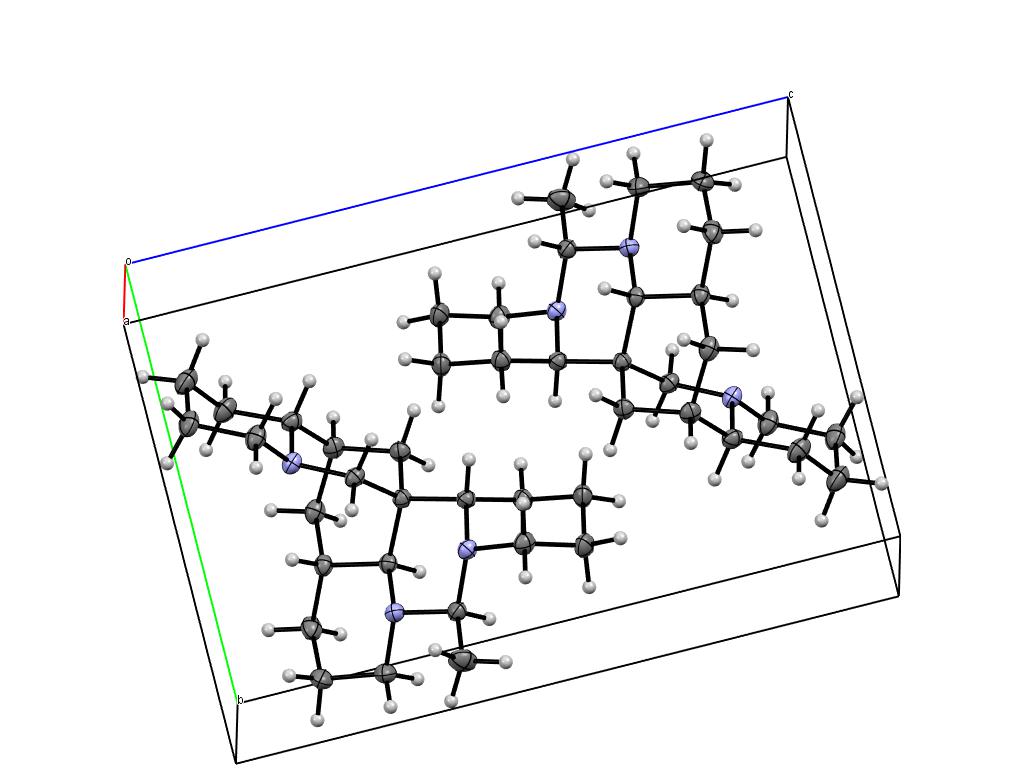


Figure S54. X-ray structure of **1**.

Table S9 Crystal data and structure refinement for **1**

| Identification code | Compound **1** |
| --- | --- |
| Empirical formula | C_22_H_37_N_3_ |
| Formula weight | 343.54 |
| Temperature/K | 170.0 |
| Crystal system | triclinic |
| Space group | P-1 |
| a/Å | 6.8445(4) |
| b/Å | 9.8999(5) |
| c/Å | 14.9406(8) |
| α/° | 88.184(2) |
| β/° | 83.162(2) |
| γ/° | 69.941(2) |
| Volume/Å^3^ | 944.15(9) |
| Z | 2 |
| ρ_calc_g/cm^3^ | 1.208 |
| μ/mm^‑1^ | 0.534 |
| F(000) | 380.0 |
| Crystal size/mm^3^ | 0.49 × 0.48 × 0.35 |
| Radiation | CuKα (λ = 1.54178) |
| 2Θ range for data collection/° | 5.958 to 137.378 |
| Index ranges | -8 ≤ h ≤ 8, -11 ≤ k ≤ 11, -18 ≤ l ≤ 18 |
| Reflections collected | 26269 |
| Independent reflections | 3461 [R_int_ = 0.0299, R_sigma_ = 0.0223] |
| Data/restraints/parameters | 3461/0/227 |
| Goodness-of-fit on F^2^ | 1.063 |
| Final R indexes [I>=2σ (I)] | R_1_ = 0.0389, wR_2_ = 0.1017 |
| Final R indexes [all data] | R_1_ = 0.0400, wR_2_ = 0.1026 |
| Largest diff. peak/hole / e Å^-3^ | 0.25/-0.21 |


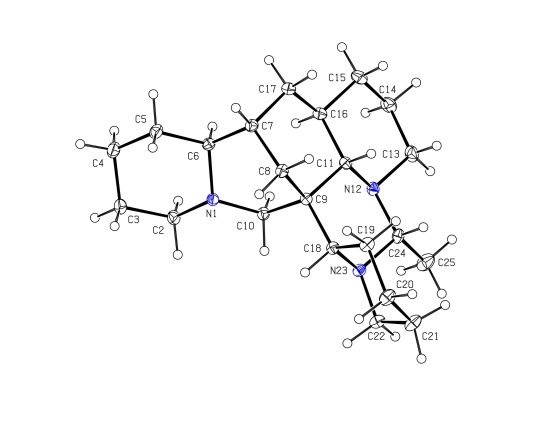


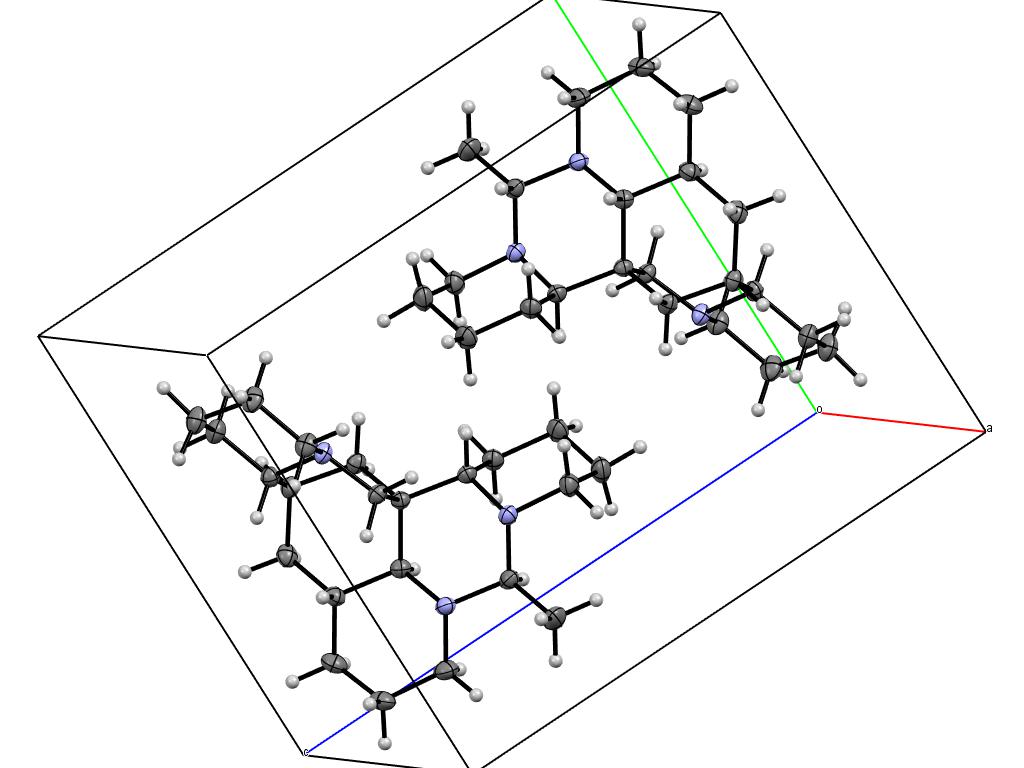


Figure S55. X-ray structure of **2**.

Table S10 Crystal data and structure refinement for **2**

| Identification code | Compound **2** |
| --- | --- |
| Empirical formula | C_22_H_37_N_3_ |
| Formula weight | 343.54 |
| Temperature/K | 170.0 |
| Crystal system | triclinic |
| Space group | P-1 |
| a/Å | 6.8085(2) |
| b/Å | 10.6845(4) |
| c/Å | 13.8387(5) |
| α/° | 90.243(2) |
| β/° | 98.8520(10) |
| γ/° | 106.3930(10) |
| Volume/Å^3^ | 953.07(6) |
| Z | 2 |
| ρ_calc_g/cm^3^ | 1.197 |
| μ/mm^‑1^ | 0.070 |
| F(000) | 380.0 |
| Crystal size/mm^3^ | 0.45 × 0.26 × 0.18 |
| Radiation | MoKα (λ = 0.71073) |
| 2Θ range for data collection/° | 4.852 to 67.15 |
| Index ranges | -9 ≤ h ≤ 10, -16 ≤ k ≤ 15, -19 ≤ l ≤ 21 |
| Reflections collected | 24182 |
| Independent reflections | 6459 [R_int_ = 0.0308, R_sigma_ = 0.0337] |
| Data/restraints/parameters | 6459/0/227 |
| Goodness-of-fit on F^2^ | 1.075 |
| Final R indexes [I>=2σ (I)] | R_1_ = 0.0533, wR_2_ = 0.1376 |
| Final R indexes [all data] | R_1_ = 0.0680, wR_2_ = 0.1452 |
| Largest diff. peak/hole / e Å^-3^ | 0.32/-0.23 |

**
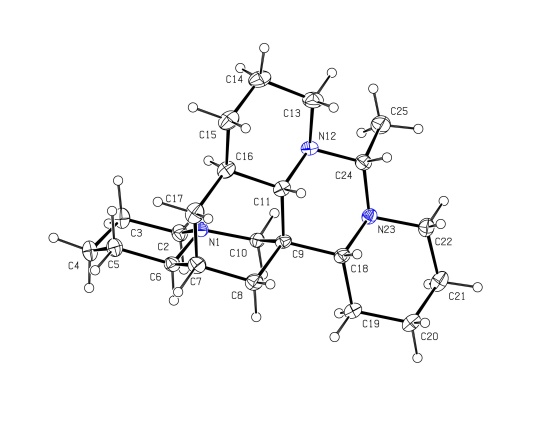
**


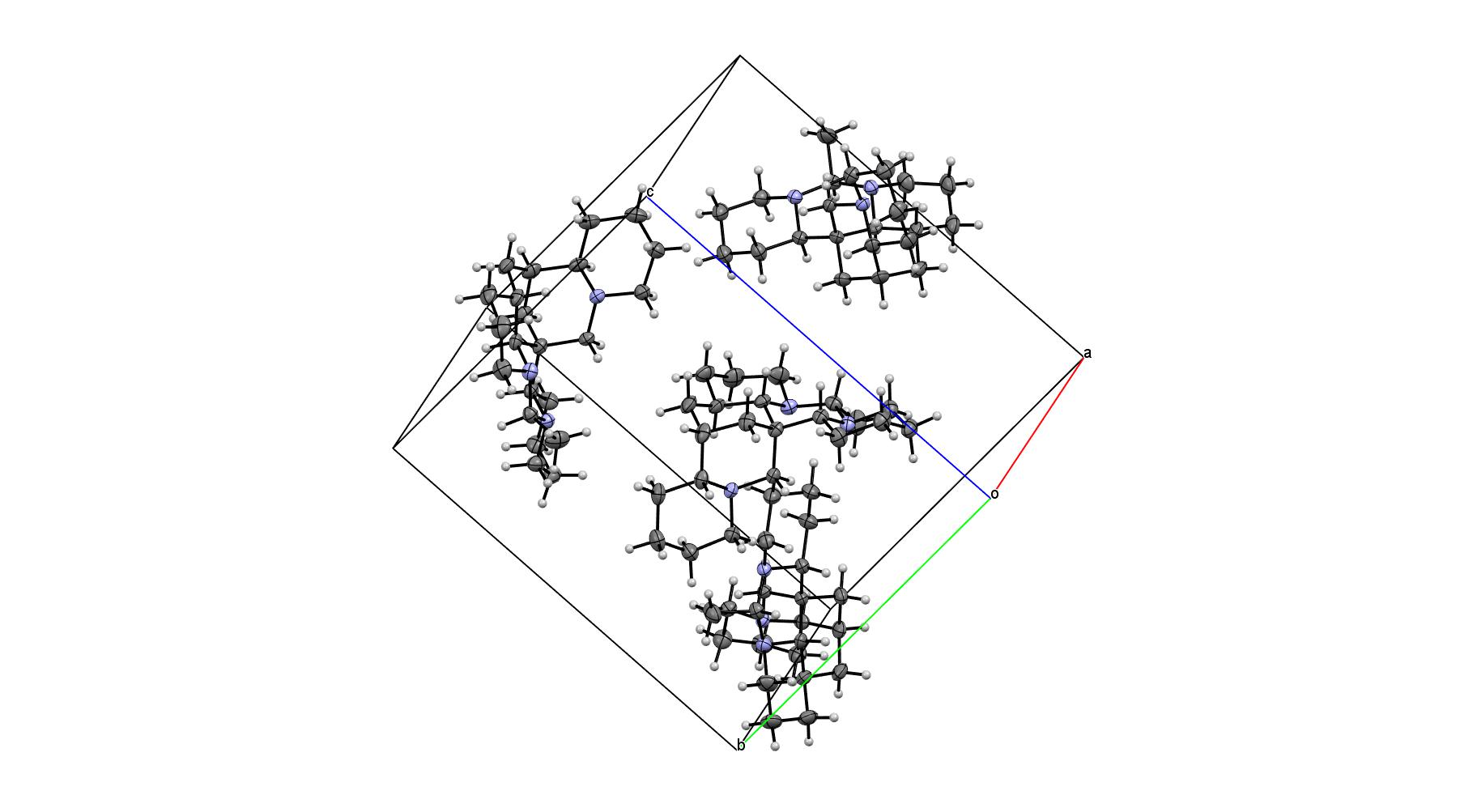


Figure S56. X-ray structure of **3**.

Table S11 Crystal data and structure refinement for **3**

| Identification code | Compound **3** |
| --- | --- |
| Empirical formula | C_22_H_37_N_3_ |
| Formula weight | 343.54 |
| Temperature/K | 170.0 |
| Crystal system | orthorhombic |
| Space group | P2_1_2_1_2_1_ |
| a/Å | 9.3517(2) |
| b/Å | 14.1492(3) |
| c/Å | 14.8653(3) |
| α/° | 90 |
| β/° | 90 |
| γ/° | 90 |
| Volume/Å^3^ | 1966.96(7) |
| Z | 4 |
| ρ_calc_g/cm^3^ | 1.160 |
| μ/mm^‑1^ | 0.513 |
| F(000) | 760.0 |
| Crystal size/mm^3^ | 0.47 × 0.36 × 0.31 |
| Radiation | CuKα (λ = 1.54178) |
| 2Θ range for data collection/° | 8.628 to 136.442 |
| Index ranges | -11 ≤ h ≤ 11, -17 ≤ k ≤ 17, -17 ≤ l ≤ 14 |
| Reflections collected | 20384 |
| Independent reflections | 3585 [R_int_ = 0.0243, R_sigma_ = 0.0174] |
| Data/restraints/parameters | 3585/0/227 |
| Goodness-of-fit on F^2^ | 1.069 |
| Final R indexes [I>=2σ (I)] | R_1_ = 0.0317, wR_2_ = 0.0838 |
| Final R indexes [all data] | R_1_ = 0.0318, wR_2_ = 0.0838 |
| Largest diff. peak/hole / e Å^-3^ | 0.12/-0.16 |
| Flack parameter | 0.04(6) |
